# Sweet-Tasting Ionic Conjugates of Local Anesthetics and Vasoconstrictors

**DOI:** 10.3390/molecules26040983

**Published:** 2021-02-12

**Authors:** John K. Neubert, Alexander A. Oliferenko, Polina V. Oliferenko, Sergey V. Emets, David A. Ostrov, Gary I. Altschuler, Joe Calkins, Jay Wickersham, Robert Hromas, Iryna O. Lebedyeva

**Affiliations:** 1 Department of Orthodontics, College of Dentistry, University of Florida, Gainesville, FL 32610, USA; jneubert@dental.ufl.edu (J.K.N.); jWickersham@gmail.com (J.W.); 2 EigenChem Technologies Inc., Alachua, FL 32615, USA; a.oliferenko@eigenchem.com (A.A.O.); p.oliferenko@eigenchem.com (P.V.O.); s.emets@eigenchem.com (S.V.E.); 3 Department of Pathology, Immunology and Laboratory Medicine, College of Medicine, University of Florida, Gainesville, FL 32610, USA; ostroda@pathology.ufl.edu; 4 Altschuler Periodontic and Implant Center, Gainesville, FL 32606, USA; gary.altschuler@ufl.edu; 5 Department of Chemistry and Physics, Augusta University, Augusta, GA 30912, USA; jcalkins3@gmail.com; 6 Department of Medicine, College of Medicine, University of Florida & Shands, Gainesville, FL 32610, USA; rhromas@ufl.edu

**Keywords:** anesthesia, local anesthetic, vasoconstrictor, sweetener, injectable anesthetic, lidocaine, local anesthesia, dental, epinephrine

## Abstract

Local anesthetics are widely utilized in dentistry, cosmetology, and medicine. Local anesthesia is essential to providing a pain-free experience during dental and local surgeries as well as cosmetic procedures. However, the injection itself may produce discomfort and be a source of aversion. A novel approach toward the taste modulation of local anesthetics is proposed, in which the anesthetics of the “-caine” family serve as cations and are coupled with anionic sweeteners such as saccharinate and acesulfamate. Ionic conjugates of vasoconstrictor epinephrine such as epinephrine saccharinate and epinephrine acesulfamate have also been synthesized. Novel ionic conjugates were developed using anion exchange techniques. Reported compounds are sweet-tasting and are safe to use both topically and as injections.

## 1. Introduction

Local anesthesia is essential for suppressing pain during medical [1,2], cosmetic [3,4,5], or dental procedures [6]. However, patients often perceive the receipt of local anesthesia as the most painful and sometimes only objectionable part of these procedures [7]. Therefore, patients may avoid obtaining cosmetic [8], medical [9], or dental care [10]. The bitter taste of the commonly used hydrochloride salts of local anesthetics [11,12] adds to the general displeasure that patients experience prior to dental procedures [13]. Further, hydrochloride salts that are commonly used in compositions formulated for local anesthesia, e.g., compositions comprising hydrochloride salt of a local anesthetic and epinephrine hydrochloride, have an acidic pH of between 3.5 and 5.5 [14] and, consequently, can cause additional pain and tissue damage upon injection [15,16]. As a result, there is an existing need in compositions for local anesthetics with higher pH that do not taste bitter or create an objectionable feeling upon injection.

**Figure 1:**
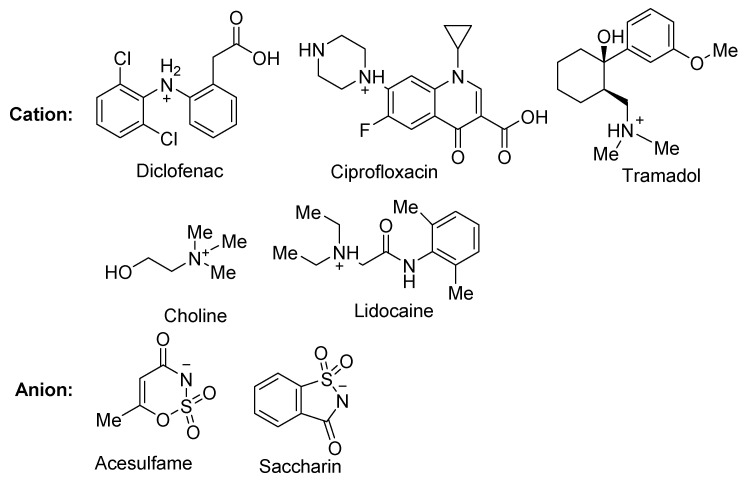
Pharmaceuticals with saccharin and acesulfame as counter ions, which have been reported in the literature.

The use of saccharinate as a counter anion in ciprofloxacin saccharinate and other fluoroquinolone antimicrobials due to its sweet taste, high aqueous solubility, and capability to form salts and co-crystals has been reported (Figure 1) [17]. Substitution of the conventional chloride anion with saccharinate in tramadol hydrochloride (Figure 1) improved the release profile and stability of the active ingredient [18]. Moreira et al. reported the synthesis of acesulfame, saccharin, and docusate salts for pharmaceuticals such as local anesthetics, as well as salicylic acid, flurbiprofen, diclofenac, and flufenamic acid (Figure 1). Saccharinate and acesulfamate have also been employed to synthesize choline salts with the sweeteners [19]. Rogers et al. mentioned saccharin and acesulfame as counter ions for active pharmaceutical, biological, and nutritional ingredients [20], as well as provided several examples of the discussed ionic liquids. In our previously reported work, injectable salts of lidocaine, such as lidocaine acesulfamate, saccharinate, and gluconate, were developed and studied for their taste, efficacy, and toxicity [21].

The bitter taste of hydrochloride salts of local anesthetics [11,22] has been hidden by glutamate salts [23], dexmedetomidine as an oral premedication [24], or a formulation of a sweet-tasting composition containing a local anesthetic and a sweetener [25]. The problem of painful injections has been addressed through the creation of high-pH buffer solutions for procedures that require injections [26] as well as through the development of new procedures that would minimize the pain resulting from the injection [7].

In this work, we have solved two problems: the bitter taste of local anesthetics and the low pH values of local anesthetic composition solutions, which results in a stinging effect upon injection. The synthesis of novel ionic conjugates has also been expanded to major representatives of injectable local anesthetic cations such as articaine, bupivacaine, mepivacaine, oxybuprocaine, prilocaine, and a vasoconstrictor epinephrine. Sweetener anions were represented by saccharin and acesulfame. Hydrochloride salts of these widely used local anesthetics have been subjected to the ion exchange reaction with metal salts of ionic sweeteners, which resulted in novel, highly soluble, and intrinsically sweet chemical compounds. The exchange of epinephrine hydrochloride for sweetener anions such as saccharin and acesulfame was also demonstrated, as epinephrine is a vasoconstrictor and an ingredient in a wide range of injectable formulations. Aqueous solutions of the synthesized compounds have been studied for their pH values, palatability, and in vivo thermal pain analgesic properties.

## 2. Results and Discussion

### 2.1. Synthesis of Novel Acesulfame and Saccharin Salts of Local Anesthetics

**Figure 2:**
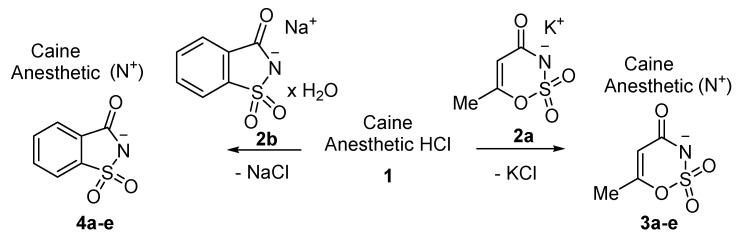
General scheme for the synthesis of saccharin and acesulfame salts of local anesthetics of the caine family.

Acesulfame salts of mepivacaine **3a**, bupivacaine **3b**, prilocaine **3c**, articaine **3d**, and oxybuprocaine **3e** were synthesized in 96–98% yields. Products were formed as a white semisolid (**3a**), colorless oil (**3b**), a yellowish oil (**3e**), and white solids (**3c**, **3d**). Saccharin salts of mepivacaine **4a**, bupivacaine **4b**, prilocaine **4c**, articaine **4d**, and oxybuprocaine **4e** were synthesized in 95–99% yields and products were isolated as a colorless oil (**4a**), a white solid (**4b** and **4c**), and a yellow oil (**4d**, **4e**). To synthesize a library of novel sweet anesthetics of the “-caine” family, the caine hydrochlorides **1** such as mepivacaine HCl, bupivacaine HCl, prilocaine HCl, and articaine HCl were mixed with either potassium acesulfame **2a** or sodium saccharin hydrate **2b** in dry MeCN for 4 h at 50 °C. The reaction mixture was then filtered to isolate the sodium or potassium chloride byproduct. After the solvent was evaporated, products **3a**–**d** and **4a**–**d** were isolated in quantitative yields. Oxybuprocaine salts **3e** and **4e** were synthesized by mixing equimolar quantities of oxybuprocaine hydrochloride and potassium acesulfame **2a** or sodium saccharinate **2b** in EtOH at room temperature overnight, thereby leading to the formation of **3e** and **4e** in 98 and 97%, respectively (Figure 2). The straightforward nature of the counter ion exchange from the chloride to the anion of a sweetener confirms that either method would be suitable for the synthesis of stable caine saccharinates or acesulfamates in high yields (Table 1).

**Figure 3:**
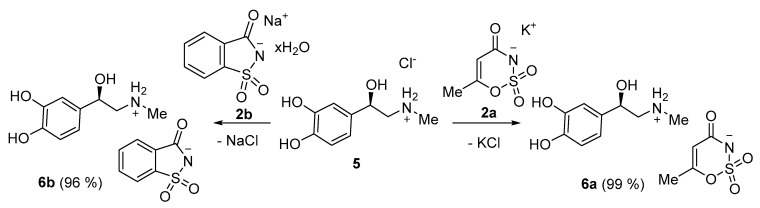
Synthesis of epinephrine acesulfamate **6a** and epinephrine saccharinate **6b**.

Epinephrine acesulfame **6a** and epinephrine saccharinate **6b** were synthesized in a similar manner to compounds **3a**–**d** and **4a**–**d**. Equimolar quantities of epinephrine hydrochloride **5** and a sweetener **2a** or **2b** were stirred in acetonitrile for 4 h at 50 °C. After the reaction mixture was filtered and the solvent had evaporated, products **6a** and **6b** were isolated in 99% and 96%, respectively (Figure 3).

^1^H NMR signals for salts **3**, **4**, and **6** revealed signals of the anesthetic moieties with the substituted aromatic signals in the weak field and also of the aliphatic protons in the strong field at 1–4.5 ppm. Acesulfame moiety in the salts was characterized by the singlet of the CH at around 5.5 ppm and the singlet of the methyl group at around 1.9 ppm. ^1^H signals for saccharin moiety were revealed as a multiplet signal of four protons at around 6.90–7.20 ppm. ^13^C signals for the acesulfame moiety of **3**, **4**, and **6** were revealed at around 18–20 ppm for the methyl group; carbon of the CO group gave a signal at around 170 ppm, while the characteristic peak of the CH group was found at around 102 ppm. Saccharin salts of **3**, **4**, and **6** gave characteristic peaks at around 171–173 ppm for the CO signal, four signals of aromatic moiety at around 128–130 ppm, and two signals at around 137 and 145 ppm for the C-C carbons of the aromatic ring. Signals of aromatic and aliphatic protons for the anesthetic cations were also found for all the compounds **3a–e** and **4a**–**e**. Mass peaks for the novel salts were identified at positive and negative ionization modes using ESI/MS. Mass signals under positive ionization were as follows: 247.1800 for **3a** and 247.1799 for **4a**, which correspond with mepivacaine residue. Signals 289.2262 for **3b** and 279.2266 for **4b** correspond with ions of positively charged bupivacaine. Positively charged signals of 221.1645 for **3c** and 221.1641 for **4c** represent prilocaine moiety. Articaine cation was detected at 285.1261 for **3d** and 285.1261 for **4d**. Epinephrine cation was detected at 184.0964 for **6a** and 184.0966 for **6b**. Acesulfame anions were detected at around *m*/*z* 161 for **3a**–**3e** and **6a** while saccharin anions were observed at around m/z 181 for **4a**–**4e** and **6b**. NMR and MS data for all products has been summarized in the Supplementary Materials section.

### 2.2. pH Study of the Aqueous Solutions of Novel Salts

The pH values of the aqueous solutions of **3a**–**e**, **4a**–**e**, and **6a**–**b** were measured and compared to the pH values of original salts **1**, **2**, and **5**. pH values of all compound solutions in deionized water were measured at 10%, 5%, and 2% concentrations. Thus, sodium saccharin monohydrate **2a** and potassium acesulfame **2b** revealed pH values of between 5.75 (10%) and 5.98 (2%) for 2a, and 5.63 (10%) and 6.19 (2%) for **2b**. From this data, it can be concluded that the use of saccharin and acesulfame as counter ions as part of the local anesthetic molecule will increase the pH of the latter, leading to a reduction in the stinging effect upon injection. The pH values of the solution of mepivacaine acesulfame **3a** and mepivacaine saccharinate **4a**, both at 10% concentration, were somewhat higher than the pH of mepivacaine hydrochloride 3.73 (10%). Bupivacaine hydrochloride revealed the pH of 4.23 at 10%, whereas bupivacaine acesulfamate **3b** and bupivacaine saccharinate **4b** showed pH values of 4.25 and 3.84 at 10%, respectively. Bupivacaine salts also showed solubility problems with hydrochloride starting materials as well as buvivacaine acesulfamate **3b** and bupivacaine saccharinate **4b** products. The pH values of prilocaine acesulfamate **3c** and prilocaine saccharinate **4c** at 10% were significantly higher than that of prilocaine hydrochloride, which was 2.10 at 10%. Articaine hydrochloride showed lower solubility in water than did the products **3d** and **4d**, which affected the measurement of pH values in a water solution. Thus, the pH of articaine hydrochloride at 10% was 4.22, whereas articaine acesulfamate **3d** and articaine saccharinate **4d** showed pH levels of 3.50 and 3.83 at 10%, respectively. Oxybuprocaine hydrochloride revealed a pH of 4.42 at 10% water concentration, while its salts showed pH levels of 4.68 and 4.70 at 10% for **3e** and **4e**, respectively. Vasoconstrictor epinephrine hydrochloride **5** showed a pH value of 3.57 at 10% water concentration, whereas epinephrine acesulfamate **6a** and epinephrine saccharinate **6b** had pH values of 4.01 and 3.57 at 10% concentration, respectively. All concentrations were measured weight-by-volume. pH values for all product concentrations have been summarized in the Supplementary Materials section.

### 2.3. Thermal Pain Testing

Response to hindpaw heat pain was determined by placing unrestrained animals on a clear glass platform under a small plastic cage and habituating the animals for 5 min. A radiant heat source was aimed directly under the ventral hindpaw surface and the time to paw withdrawal was recorded as described previously [27]. Baseline responses were obtained under naïve conditions (e.g., no injection), while post-treatment effects of caine salts **3** and **4**, and vehicle (water), were assessed for 10 min following plantar injection (100 μL). A cutoff of 32 sec was used to prevent tissue damage.

### 2.4. Statistical Analyses

An analysis of variance was used to evaluate the effects of treatment on hindpaw withdrawal latency (SPSS Inc). When significant differences were found, post hoc comparisons were made using the Tukey post hoc test. * *p* < 0.05 was considered significant in all instances.

**Figure 4:**
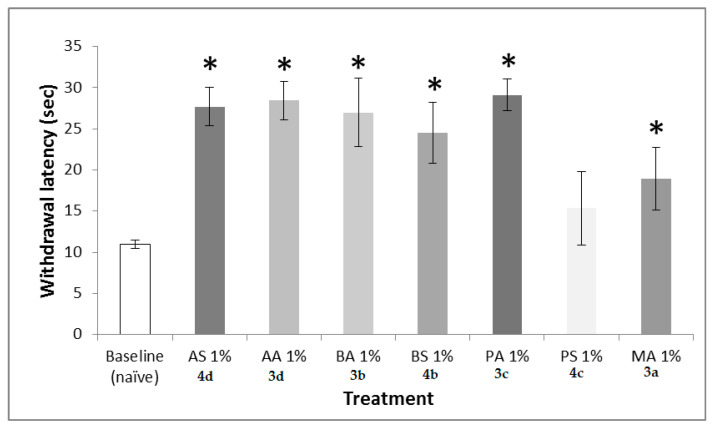
Thermal hindpaw latency following local anesthetic injection. One-percent solutions were injected into the hindpaws of animals and withdrawal latency to a thermal stimulus was recorded 10 min post-injection. All caine salts (N=10 animals/group) except PS (prilocaine saccharinate, **4c**) produced a significant increase in latency time as compared to that of non-injected naïve animals, indicating that the caine salts were effective at inhibiting pain. * *p* < 0.05

There was a significant treatment effect on hindpaw latency (F7,63 = 8.238, *p* < 0.001), with articaine saccharinate (AS) **4d**, articaine acesulfamate (AA) **3d**, bupivacaine acesulfamate (BA) **3b**, bupivacaine saccharinate (BS) **4b**, prilocaine acesulfamate (PA) **3c**, prilocaine saccharinate (PS) **4c**, and mepivacaine acesulfamate (MA) **3a** producing significantly higher latencies as compared to the baseline (naïve) (Figure 4).

### 2.5. Palatability Assessment

**Figure 5:**
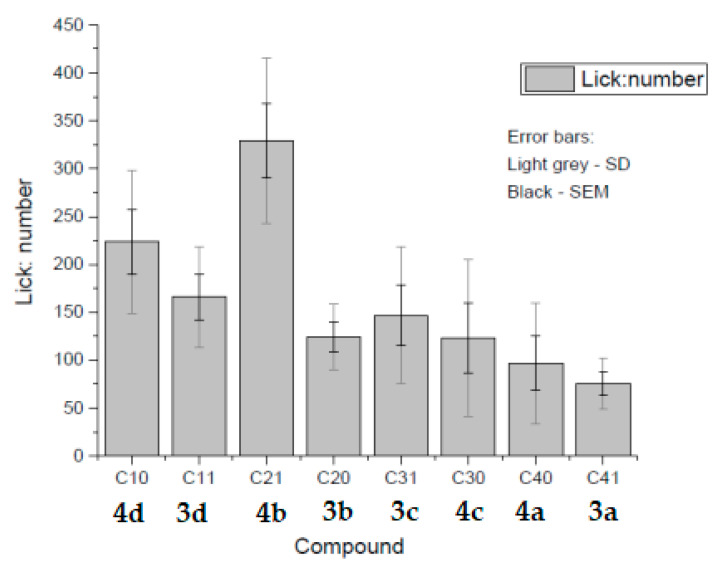
Relative palatability of each compound. A palatability test was conducted for the following anesthetics: articaine saccharinate (C10) **4d**, articaine acesulfamate (C11) **3d**, bupivacaine saccharinate (C20) **4b**, bupivacaine acesulfamate (C21) **3b**, prilocaine saccharinate (C30) **4c**, prilocaine acesulfamate (C31) **3c**, mepivacaine saccharinate (C40) **4a**, and mepivacaine acesulfamate (C41) **3a**.

Animals were food-fasted (12–15 h) prior to testing. Animals were then placed in the Orofacial Pain Assessment Device (OPAD, Stoelting Company, Wood Dale, IL, USA), which consisted of a holding chamber and a bottle containing various test solutions. Animals (N = 5/solution) were placed in the holding chamber and allowed access to the solution for 7 min. The number of solution licking events was automatically recorded (Figure 5).

Rats successfully consumed the different caine salt solutions. Note that our previous study [28] demonstrated that rats given a 0.1% saccharin solution had 109 ± 40 licks (in a 20-min test session).

## 3. Materials and Methods

Melting points were determined on a capillary point apparatus with a digital thermometer and are uncorrected. ^1^H NMR spectra were recorded at 500 MHz while ^13^C NMR spectra were recorded at 125 MHz on a Bruker spectrometer (Bruker, Billerica, MA, USA) at room temperature. Chemical shifts are reported in ppm relative to TMS as the internal standard (^1^HNMR) or to the residual solvent peak (^13^C NMR). HRMS were recorded on a QTof-USA (Thermo Fisher Scientific, Waltham, MA, USA) spectrometer operating in the ESI mode. Elemental analysis for CHN elements was carried out using 2400 CHNS Organic Elemental Analyzer 100V. All commercially available substrates were used as received, without further purification.

### 3.1. Animal Studies

Male Sprague Dawley rats (200–300g, Charles River Laboratories, Wilmington, MA, USA) were housed in groups of two and were maintained in a standard 12-h light/dark cycle. Testing was completed during the light portion of the cycle, between 09:00–12:00. Animals were placed in the behavioral procedure room 30 min prior to testing, to acclimate. When the animals were not in testing sessions, food and water were made available to them ad libitum. Animal testing procedures complied with the ethical guidelines and standards established by the University of Florida’s Institutional Animal Care and Use Committee and with the Guide for Care and Use of Laboratory Animals (National Research Council Guide for the Care and Use of Laboratory Animals. Washington, D.C., National Academy Press; 1996). Animal Use Approval: The University of Florida’s Institutional Animal Care and Use Committee (IACUC) protocol #201207690; date of approval: 12/11/2012.

### 3.2. Synthesis of Acesulfamates and Saccharinates for the Caines ***3a***–***d***, and ***4a***–***d***

To the solution of a hydrochloride salt of a corresponding caine **1** (1.0 mmol) in MeCN (15 mL), the equimolar quantity of sweetener sodium salt (sodium 6-methyl-1,2,3-oxathiazin-4-olate 2,2-dioxide 0.185 g for **2a** or sodium benzo[*d*]isothiazol-3-olate 1,1-dioxide 0.205 g for **2b**) was added. The mixture was then stirred for 4 h at 50 °C. After the reaction was completed, the reaction mixture was filtered through the 22-micron membrane filter and the filtrate was taken to dryness. Next, diethyl ether (3 × 25 mL) was added to the product and was evaporated to give compounds **3a**–**d** and **4a**–**d** in quantitative yields. All final products, **3a**–**d** and **4a**–**d**, are water-soluble ionic conjugates consisting of the anion of a sweetener (acesulfame or saccharine) and a representative of a caine family (mepivacaine, bupivacaine, prilocaine, articaine) as a cation.

### 3.3. Synthesis of Oxybuprocaine Acesulfame ***3e*** and Saccharinate ***4e***

To a solution of oxybuprocaine hydrochloride (0.048 g, 0.139 mmol) in 2 mL of EtOH was added a suspension of acesulfame potassium (0.028 g, 0.139 mg) or sodium saccharinate (0.028 g, 0.139 mg) in 2 mL of EtOH. The reaction mixture was stirred at room temperature overnight. This reaction mixture was then filtered, and the solvent evaporated. The residue was dissolved in 3 mL of MeCN. After filtration, the products were obtained as yellow oils in a quantitative yield.

### 3.4. Synthesis of Epinephrine Acesulfamate ***6a*** and Epinephrine Saccharinate ***6b***

To the solution of epinephrine hydrochloride **5** (1.0 mmol, 0.22 g) in MeCN (15 mL), the equimolar quantity of sweetener sodium salt (sodium 6-methyl-1,2,3-oxathiazin-4-olate 2,2-dioxide 0.185 g for **2a** and sodium benzo[*d*]isothiazol-3-olate 1,1-dioxide 0.205 g for **2b**) was added. The mixture was then stirred for 4 h at 50 °C. After the reaction was completed, the reaction mixture was filtered through the 22-micron membrane filter and the filtrate was taken to dryness. Next, diethyl ether (3 × 25 mL) was added to the product and it was evaporated to give the compounds **6a**,**b** in quantitative yields.

## 4. Conclusions

Novel sweet-tasting salts of anesthetics of the “-caine” family, such as articaine, bupivacaine, mepivacaine, oxybuprocaine, and prilocaine, as well as salts of vasoconstrictor epinephrine, have been synthesized. Ionic conjugates have been developed by coupling hydrochloride salts of local anesthetics with sodium saccharinate and potassium acesulfamate, using either in MeCN or EtOH as a solvent. An analogous approach was employed to synthesize epinephrine salts with the saccharin and acesulfame sweetener counter ions. Chloride anion, which is largely responsible for the bitterness of hydrochloride salts, has been removed from the ionic liquids in the form of sodium or potassium chloride. This chemical approach toward making ionic conjugates differs greatly from reported attempts to mask the bitterness of the hydrochloride salts of anesthetics by mixing them with sweeteners or taste modifiers. Such an anion exchange results in the formation of novel, highly water-soluble, and sweet-tasting stable salts with zero osmolarity and a higher bioavailability due to the use of more lipophilic anions [29]. Animal studies of palatability of the novel salts have proved that novel caine saccharinates and acesulfamates possess a more pleasant taste than do their hydrochloride analogs. During the thermal pain testing, there was a significant treatment effect on hindpaw withdraw latency (F7,63 = 8.238, *p* < 0.001), with novel ionic conjugates producing significantly higher latencies as compared to the baseline (naïve). The pH study of the aqueous solutions of all synthesized salts demonstrated that acesulfame and saccharin salts of local anesthetics showed higher pH values of water solutions of 10%, 5%, and 2% concentrations for articaine, bupivacaine, mepivacaine, oxybuprocaine, and prilocaine saccharinates and for acesulfamates as compared to their original hydrochloride salts. Aqueous solutions of novel epinephrine salts also revealed an increase in pH at 10% compared to analogous solutions of epinephrine hydrochloride. It is expected that more basic products will not have the stinging effect upon injection, unlike their counterparts with lower pH values.

## Figures and Tables

**Table 1:** Structures of the new products.

Compound Name and Structure	Compound Name and Structure
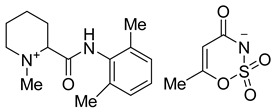 Mepivacaine acesulfamate **3a**	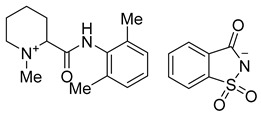 Mepivacaine saccharinate **4a**
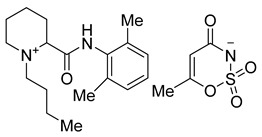 Bupivacaine acesulfamate **3b**	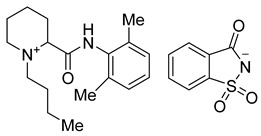 Bupivacaine saccharinate **4b**
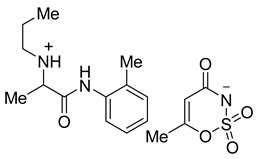 Prilocaine acesulfamate **3c**	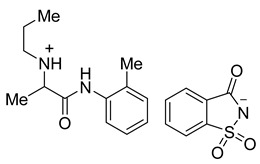 Prilocaine saccharinate **4c**
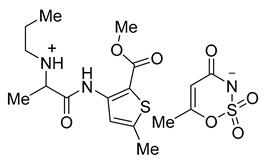 Articaine acesulfamate **3d**	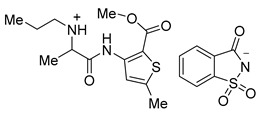 Articaine saccharinate **4d**
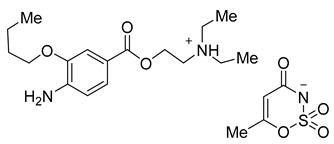 Oxybuprocaine acesulfamate **3e**	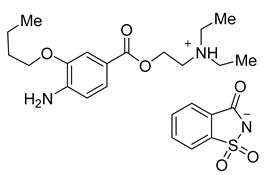 Oxybuprocaine saccharinate **4e**

## Data Availability

The data presented in this study are available in supplementary material.

## Supplementary Materials

The following are available online: Detailed analysis of compound structure for all synthesized compounds. Table S1: pH of aqueous solutions of all synthesized salts and original compounds. ^1^H, ^13^C NMR, and HRMS data on all synthesized compounds.

## Sample Availability

Samples of the compounds are not available from the authors.
