# Supplementary material for: Sweet-Tasting Ionic Conjugates of Local Anesthetics and Vasoconstrictors
Source: Molecules. 2021 Feb 12;26(4):983. doi: 10.3390/molecules26040983 (PMC7918242; doi:10.3390/molecules26040983)

Supporting Information

Title Sweet-Tasting Ionic Conjugates of Local Anesthetics and Vasoconstrictors

John K. Neubert[1], Alexander A. Oliferenko[2], Polina V. Oliferenko[2], Sergey V. Emets[2], David A. Ostrov[3], Gary I. Altschuler[4], Joe Calkins[5], Jay Wickersham[1], Robert Hromas[6], and Iryna O. Lebedyeva[5,*]

[1] Department of Orthodontics, College of Dentistry, University of Florida, Gainesville, FL, USA
[2] EigenChem Technologies Inc., Alachua, FL, USA
[3] Department of Pathology, Immunology and Laboratory Medicine, College of Medicine, University of Florida, Gainesville, FL, USA
[4] Altschuler Periodontic and Implant Center, Gainesville, FL, USA
[5] Department of Chemistry and Physics, Augusta University, Augusta, GA, USA
[6] Department of Medicine, College of Medicine, University of Florida & Shands, Gainesville, FL, USA
* Correspondence: ilebedyeva@augusta.edu.

*Data on compound structure for **3a-3e, 4a-4e** and **6a-6b**.*

The following abbreviations are used to describe spin multiplicity: s = singlet, d = doublet, t = triplet, q = quartet, m = multiplet, brs = broad singlet, dd = doublet of doublets.

Mepivacaine acesulfamate **3a**: White semisolid (97%, 0.396 g, 0.97 mmol); mp 84.0 – 86.0 ^oC; ^1H NMR (500 MHz, CD_3OD, δ): 7.21 – 7.09 (m, 3H), 5.53 (s, 1H), 4.17 (dd, J = 11.9, 3.2 Hz, 1H), 3.54 (d, J = 12.5 Hz, 1H), 3.18 (td, J = 12.4, 3.1 Hz, 1H), 2.93 (d, J = 1.1 Hz, 3H), 2.46 – 2.33 (m, 1H), 2.22 (d, J = 0.9 Hz, 6H), 2.02 (t, J = 1.0 Hz, 3H), 1.93 (s, 3H), 1.89 – 1.67 (m, 2H); ^{13}C NMR (125 MHz, CD_3OD, δ): 173.2, 168.8, 164.4, 137.2, 134.7, 129.9, 129.5, 102.8, 68.8, 56.7, 43.6, 31.0, 24.6, 22.9, 20.4, 19.1. HRMS (ESI) *m*/*z*: $[M + H]^+$ calcd for $C_{15}H_{22}N_2O$ 246.3535, found 247.1800; HRMS (ESI) *m*/*z*: $[M − H]^-$ calcd for $C_4H_4NO_4S$ 161.9867, found 161.9873. Anal. Calcd for: $C_{19}H_{26}N_3O_5S$: C, 55.87; H, 6.42; N, 10.29%. Found: C, 55.76; H, 6.38; N, 10.34%.*Bupivacaine acesulfamate* **3b**: Colorless oil (96%, 0.432 g, 0.96 mmol); ^1H NMR (500 MHz, CD_3OD, δ): 7.18 – 7.11 (m, 3H), 5.55 (s, 1H), 4.32 (d, J = 11.6 Hz, 1H), 3.69 (d, J = 12.2 Hz, 1H), 3.19 (dq, J = 16.5, 11.0, 8.5 Hz, 3H), 2.42 (d, J = 12.2 Hz, 1H), 2.24 (s, 6H), 2.04 (s, 3H), 1.99 – 1.70 (m, 7H), 1.41 (q, J = 7.4 Hz, 2H), 0.99 (t, J = 7.3 Hz, 3H); ^{13}C NMR (125 MHz, CD_3OD, δ): 173.2, 169.0, 164.4, 137.2, 134.7, 129.9, 129.9, 129.5, 102.9, 57.8, 53.7, 31.0, 27.3, 24.4, 21.5, 21.5, 20.4, 19.1, 14.4. HRMS (ESI) *m*/*z*: $[M + H]^+$ calcd for $C_{18}H_{28}N_2O$ 288.2196, found 289.2262; HRMS (ESI) *m*/*z*: $[M − H]^-$ calcd for $C_4H_4NO_4S$ 161.9867, found 161.9872. Anal. Calcd for: $C_{22}H_{32}N_3O_5S$: C, 58.65; H, 7.16; N, 9.33%. Found: C, 58.54; H, 7.09; N, 9.38%.

Prilocaine acesulfamate **3c**: White solid (98%, 0.375 g, 0.98 mmol); mp 145.2 – 145.8 ^oC; ^1H NMR (500 MHz, CD_3OD, δ): 7.38 – 7.34 (m, 1H), 7.25 – 7.15 (m, 3H), 5.52 (s, 1H), 4.20 (q, J = 0.6 Hz, 1H), 3.12 – 2.92 (m, 2H), 2.26 (s, 3H), 2.02 (s, 3H), 1.71 (dd, J = 23.5, 7.3 Hz, 2H), 1.66 (d, J = 0.6 Hz, 3H), 1.02 (t, J = 7.4 Hz, 3H); ^{13}C NMR (125 MHz, CD_3OD, δ): 173.0, 169.6, 164.2,

136.1, 134.6, 132.0, 132.0, 128.3, 127.7, 127.1, 102.5, 57.9, 49.39, 21.1, 18.4, 17.2, 11.5. HRMS (ESI) *m*/*z*: $[M + H]^+$ calcd for $C_{13}H_{20}N_2O$ 220.1576, found 221.1645; HRMS (ESI) *m*/*z*: $[M - H]^-$ calcd for $C_4H_4NO_4S$ 161.9867, found 161.9875. Anal. Calcd for: $C_{17}H_{24}N_3O_5S$: C, 53.39; H, 6.33; N, 10.99%. Found: C, 53.28; H, 6.30; N, 11.29%.

Articaine acesulfamate **3d**: White solid (98%, 0.438 g, 0.98 mmol); mp 166.2 – 169.8 °C; ^{1}H NMR (500 MHz, CD_3OD, δ): 7.41 – 7.40 (m, 1H), 3.83 – 3.82 (m 4H), 3.08 – 3.04 (m, 2H), 2.13 (t, *J* = 1.4 Hz, 3H), 2.04 – 2.03 (m, 4H), 1.78 – 1.70 (m, 5H), 1.06 – 1.01 (m, 3H); ^{13}C NMR (125 MHz, CD_3OD, δ): 173.3, 169.8, 164.5, 163.6, 140.2, 138.5, 128.75, 125.4, 102.8, 58.1, 53.0, 49.78, 21.3, 20.4, 17.4, 14.6, 11.8. HRMS (ESI) *m*/*z*: $[M + H]^+$ calcd for $C_{13}H_{20}N_2O_3S$ 284.1195, found 285.1261; HRMS (ESI) *m*/*z*: $[M - H]^-$ calcd for $C_4H_4NO_4S$ 161.9867, found 161.9875. Anal. Calcd for: $C_{17}H_{24}N_3O_7S_2$: C, 45.73; H, 5.42; N, 9.41%. Found: C, 45.67; H, 5.38; N, 9.50%.

Oxybuprocaine acesulfame **3e**: Yellowish oil (98%, 0.462 g, 0.98 mmol); ^{1}H NMR (500 MHz, CD_3OD, δ): 7.43 (d, *J* = 43.7 Hz, 2H), 6.68 (s, 1H), 5.44 (s, 1H), 4.56 (br s, 2H), 4.0 (br s, 2H), 3.56 (s, 2H), 1.97 (d, *J* = 12.1 Hz, 2H), 1.77 (br s, 2H), 1.5 (br s, 2H), 1.33 (br s, 6H), 0.98 (t, *J* = 10 Hz, 3H); ^{13}C NMR (125 MHz, CD_3OD, δ): 173.0, 168.2, 164.1, 146.9, 145.3, 126.0, 118.0, 114.1, 113.5, 102.5, 69.6, 60.0, 52.4, 32.7, 20.6, 20.1, 14.5, 9.5. HRMS (ESI) *m*/*z*: $[M + H]^+$ calcd for $C_{17}H_{29}N_2O_3$ 309.2173, found 309.2172; HRMS (ESI) *m*/*z*: $[M - H]^-$ calcd for $C_4H_4NO_4S$ 161.9867, found 161.9870. Anal. Calcd for: $C_{21}H_{33}N_3O_7S$: C, 53.49; H, 7.05; N, 8.91%. Found: C, 53.40; H, 7.01; N, 8.98%.

Mepivacaine saccharinate **4a**: Colorless oil (95%, 0.407 g, 0.95 mmol); ^{1}H NMR (500 MHz, CD_3OD, δ): 7.82 – 7.76 (m, 2H), 7.75 – 7.65 (m, 2H), 7.18 – 7.09 (m, 3H), 4.20 (dd, *J* = 11.8, 3.3 Hz, 1H), 3.53 (d, *J* = 12.4 Hz, 1H), 3.18 (td, *J* = 12.5, 3.1 Hz, 1H), 2.94 (s, 3H), 2.39 (dd, *J* = 14.0, 3.5 Hz, 1H), 2.39 (d, *J* = 3.4 Hz, 1H), 2.27 (s, 6H), 1.95 (d, *J* = 12.6 Hz, 2H), 1.90 – 1.60 (m, 2H);

^{13}C NMR (125 MHz, CD_3OD, δ): 172.2, 168.5, 145.7, 136.9, 135.1, 134.4, 134.1, 133.7, 129.5, 129.2, 124.66, 121.2, 68.4, 56.4, 43.3, 30.6, 24.2, 22.5, 18.8. HRMS (ESI) *m*/*z*: $[M + H]^+$ calcd for $C_{15}H_{22}N_2O$ 246.1727, found 247.1799; HRMS (ESI) *m*/*z*: $[M - H]^-$ calcd for $C_7H_4NO_3S$ 181.9917, found 181.9924. Anal. Calcd for: $C_{22}H_{26}N_3O_4S$: C, 61.66; H, 6.12; N, 9.81%. Found: C, 61.65; H, 6.09; N, 9.84%.

Bupivacaine saccharinate **4b***:* White solid (97%, 0.456 g, 0.97 mmol); mp 162.4 – 164.8 °C. ^1H NMR (500 MHz, DMSO-d_6, δ): 10.17 (s, 1H), 9.70 (s, 1H), 7.64 – 7.57 (m, 1H), 7.58 (s, 1H), 7.13 (m, 3H), 4.07 (s, 1H), 3.52 (d, *J* = 12.4 Hz, 1H), 3.11 – 3.01 (m, 4H), 2.15 (s, 6H), 1.92 – 1.52 (m, 7H), 1.31 (q, *J* = 7.4 Hz, 2H), 0.90 (t, *J* = 7.3 Hz, 3H); ^{13}C NMR (125 MHz, CD_3OD, δ): 172.4, 168.7, 145.8, 136.8, 136.8, 135.2, 134.4, 134.07, 133.7, 129.6, 129.2, 124.7, 121.2, 57.4, 53.3, 30.7, 27.0, 24.0, 21.2, 18.7, 14.1. HRMS (ESI) *m*/*z*: $[M + H]^+$ calcd for $C_{18}H_{28}N_2O$ 288.2196, found 289.2266; HRMS (ESI) *m*/*z*: $[M - H]^-$ calcd for $C_7H_4NO_3S$ 181.9917, found 181.9924. Anal. Calcd for: $C_{25}H_{32}N_3O_4S$: C, 63.81; H, 6.85; N, 8.93%. Found: C, 63.77; H, 6.82; N, 8.99%.

Prilocaine saccharinate **4c***:* White solid (96%, 0.386 g, 0.96 mmol); mp 122.0 – 123.4 °C; ^1H NMR (500 MHz, CD_3OD, δ): 7.81 – 7.69 (m, 3H), 7.41 – 7.38 (dd, *J* = 7.3, 2.0 Hz, 1H), 7.30 – 7.20 (m, 4H), 4.22 (q, *J* = 7.0 Hz, 1H), 3.11 – 3.05 (m, 2H), 2.29 (s, 3H), 1.78 (m, 5H), 1.06 (t, *J* = 7.4 Hz, 3H); ^{13}C NMR (125 MHz, CD_3OD, δ): 172.7, 169.9, 146.1, 136.4, 135.5, 134.9, 134.4, 134.0, 132.4, 128.6, 128.1, 127.4, 125.0, 121.6, 58.3, 49.7, 21.5, 18.7, 17.6, 11.9. HRMS (ESI) *m*/*z*: $[M + H]^+$ calcd for $C_{13}H_{20}N_2O$ 220.1576, found 221.1646; HRMS (ESI) *m*/*z*: $[M - H]^-$ calcd for $C_7H_4NO_3S$ 181.9917, found 181.9923. Anal. Calcd for: $C_{20}H_{24}N_3O_4S$: C, 59.68; H, 6.01; N, 10.44%. Found: C, 59.64; H, 5.97; N, 10.49%.

Articaine saccharinate **4d***:* Yellow oil (99%, 0.462 g, 0.99 mmol); ^1H NMR (500 MHz, CD_3OD, δ): 8.79 – 8.76 (m, 2H), 8.69 – 8.66 (m, 2H), 8.39 (t, *J* = 1.1 Hz, 1H), 5.31 (d, *J* = 6.0 Hz, 1H),

4.81 – 4.80 (m, 3H), 4.10 – 4.05 (m, 2H), 3.11 (dt, *J* = 2.1, 1.0 Hz, 3H), 2.73 (d, *J* = 7.0 Hz, 5H), 2.01 (t, *J* = 7.4 Hz, 3H); ^{13}C NMR (125 MHz, CD_3OD, *δ*): 172.7, 169.8, 163.5, 145.9, 140.1, 138.4, 138.4, 134.3, 133.9, 128.8, 124.9, 124.9, 121.5, 58.1, 53.0, 49.8, 21.3, 17.5, 14.6, 11.8. HRMS (ESI) *m*/*z*: $[M + H]^+$ calcd for $C_{13}H_{20}N_2O_3S$ 284.1195, found 285.1261; HRMS (ESI) *m*/*z*: $[M - H]^-$ calcd for $C_7H_4NO_3S$ 181.9917, found 181.9925. Anal. Calcd for: $C_{20}H_{24}N_3O_6S_2$: C, 51.49; H, 5.19; N, 9.01%. Found: C, 51.41; H, 5.12; N, 9.11%.

Oxybuprocaine saccharinate **4e**: Yellow oil (97%, 0.462 g, 0.98 mmol); ^{1}H NMR (500 MHz, CD_3OD, *δ*): 7.76-7.59 (m, 4H), 7.49-7.34 (m, 2H), 6.65 (d, *J* = 31.7 Hz, 1H), 4.57 (m, 2H), 3.99 (m, 2H), 2.59 (m, 2H), 3.32 (m, 4H), 1.80 (br s, 2H), 1.52 (br s, 2H), 1.35 (t, *J* = 15.0 Hz, 6H), 1.01 (m, 3H); ^{13}C NMR (125 MHz, CD_3OD, *δ*): 172.5, 168.2, 146.9, 145.8, 145.3, 135.2, 133.9, 133.5, 126.0, 124.7, 121.2, 118.0, 114.0, 113.4, 69.5, 60.0, 52.4, 32.7, 20.6, 14.5, 9.5. HRMS (ESI) *m*/*z*: $[M + H]^+$ calcd for $C_{17}H_{29}N_2O_3$ 309.2173, found 309.2171; HRMS (ESI) *m*/*z*: $[M - H]^-$ calcd for $C_4H_4NO_4S$ 181.9917, found 181.9920. Anal. Calcd for: $C_{24}H_{33}N_3O_6S$: C, 58.64; H, 6.77; N, 8.55%. Found: C, 58.49; H, 6.70; N, 8.65%.

Epinephrine acesulfamate **6a**: Colorless oil (99%, 0.343, 0.99 mmol); ^{1}H NMR (500 MHz, CD_3OD, *δ*): 6.88 (s, 1H), 6.79 – 6.72 (m, 2H), 5.53 (s, 1H), 4.83 – 4.79 (m, 1H), 3.18 – 3.04 (m, 2H), 2.74 (s, 3H), 2.05-2.04 (m 3H); ^{13}C NMR (125 MHz, CD_3OD, *δ*): 172.0, 163.2, 145.7, 145.6, 132.8, 117.7, 115.6, 113.4, 100.4, 69.0, 55.9, 33.0, 19.0. HRMS (ESI) *m*/*z*: $[M + H]^+$ calcd for $C_9H_{14}NO_3$ 184.0968, found 184.0964; HRMS (ESI) *m*/*z*: $[M - H]^-$ calcd for $C_4H_4NO_4S$ 161.9867, found 161.9866. Anal. Calcd for: $C_{13}H_{18}N_2O_7S$: C, 45.08; H, 5.24; N, 8.09%. Found: C, 45.00; H, 5.19; N, 8.16.

Epinephrine saccharinate **6b**: Yellow solid (96%, 0.352 g, 0.96 mmol); mp 82.0 – 83.2 ^{o}C; ^{1}H NMR (500 MHz, CD_3OD, *δ*): 7.72 – 7.63 (m, 2H), 6.93 (d, *J* = 2.0 Hz, 1H), 6.82 – 6.72 (m, 2H),

4.88 (dd, J = 9.4, 3.9 Hz, 3H), 2.77 (s, 3H), 2.02 (d, J = 0.6 Hz, 3H); ^{13}C NMR (125 MHz, CD_3OD, δ): 171.3, 145.4, 145.3, 144.2, 133.7, 132.9, 132.6, 132.5, 123.5, 120.0, 117.7, 115.5, 113.3, 68.8, 55.7, 32.9. HRMS (ESI) m/z: $[M + H]^+$ calcd for $C_9H_{14}NO_3$ 184.0968, found 184.0966; HRMS (ESI) m/z: $[M - H]^-$ calcd for $C_7H_4NO_3S$ 181.9917, found 181.9918. Anal. Calcd for: $C_{16}H_{18}N_2O_6S$: C, 52.45; H, 4.95; N, 7.65%. Found: C, 52.39; H, 4.89; N, 7.74.

Table S1. *pH of synthesized salts and original compounds.*

Potassium acesulfame **2a** 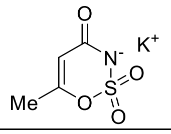	Weight-by-volume conc.	pH
	10%	5.78
	5%	6.33
	2%	5.98
Sodium saccharin hydrate **2b** 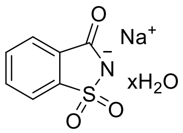	Weight-by-volume conc.	pH
	10%	5.63
	5%	6.09
	2%	6.19
Mepivacaine hydrochloride 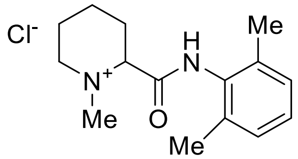	Weight-by-volume conc.	pH
	10%	3.73
	5%	3.77
	2%	4.53
Mepivacaine acesulfamate **3a** 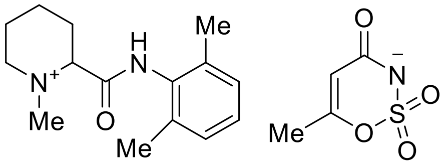	Weight-by-volume conc.	pH
	10%	4.00
	5%	4.87

	2%	4.91
Mepivacaine saccharinate **4a** 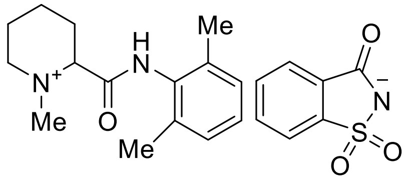	Weight-by-volume conc.	pH
	10%	3.95
	5%	4.06
	2%	4.23
Bupivacaine hydrochloride 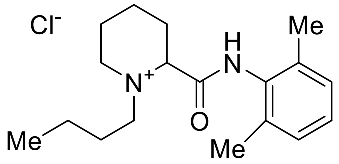 (low solubility in water)	Weight-by-volume conc.	pH
	10%	4.23
	5%	4.81
	2%	5.22
Bupivacaine acesulfamate **3b** 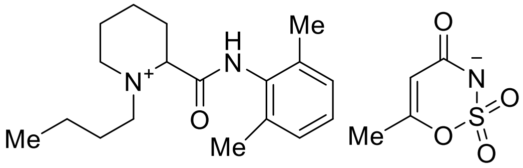 (low solubility in water)	Weight-by-volume conc.	pH
	10%	4.25
	5%	5.13
	2%	5.38
Bupivacaine saccharinate **4b** 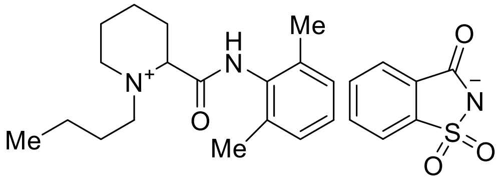 (low solubility in water)	Weight-by-volume conc.	pH
	10%	3.84
	5%	3.66
	2%	5.19
Prilocaine hydrochloride	Weight-by-volume conc.	pH
	10%	2.10

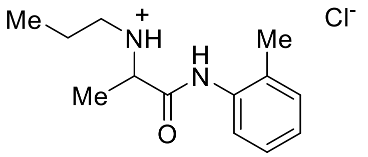	5%	3.42
	2%	4.60
Prilocaine acesulfamate **3c** 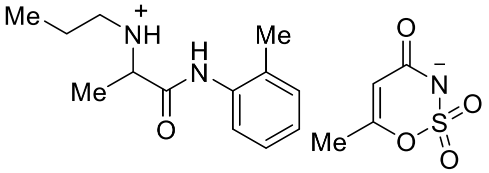	Weight-by-volume conc.	pH
	10%	4.71
	5%	5.00
	2%	5.16
Prilocaine saccharinate **4c** 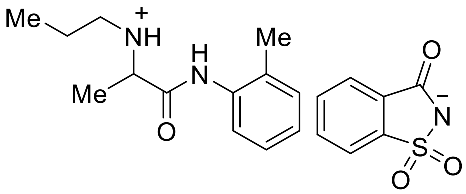	Weight-by-volume conc..	pH
	10%	4.47
	5%	4.96
	2%	5.04
Articaine hydrochloride 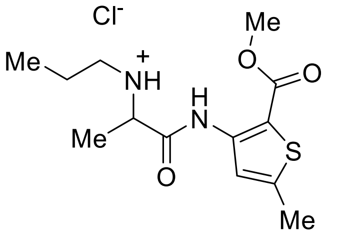 (low solubility in water)	Weight-by-volume conc.	pH
	10%	4.22
	5%	4.62
	2%	4.78
Articaine acesulfamate **3d** 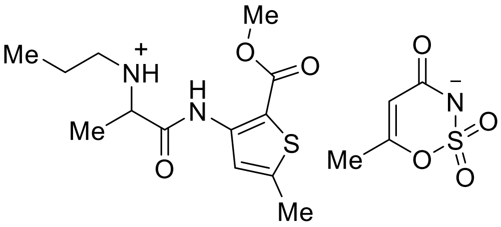	Weight-by-volume conc.	pH
	10%	3.50
	5%	3.79
	2%	3.95
	Weight-by-volume conc.	pH
	10%	3.83
Articaine saccharinate **4d**	5%	3.95

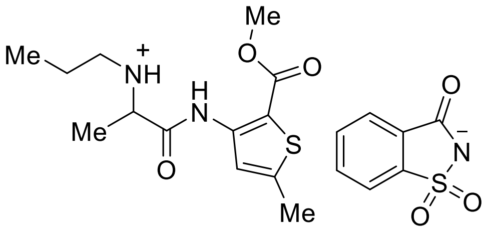	2%	4.15
Oxybuprocaine hydrochloride 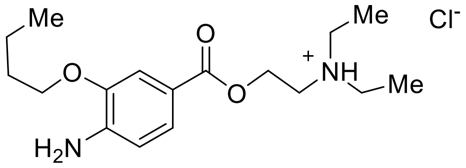	Weight-by-volume conc.	pH
	10%	4.42
	5%	5.33
	2%	5.53
Oxybuprocaine acesulfamate **3e** 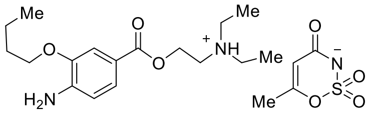	Weight-by-volume conc.	pH
	10%	4.68
	5%	5.22
	2%	5.56
Oxybuprocaine saccharinate **4e** 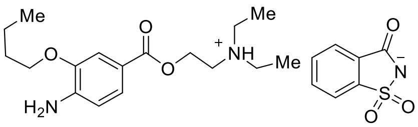	Weight-by-volume conc.	pH
	10%	4.70
	5%	5.21
	2%	5.71
Epinephrine hydrochloride **5** 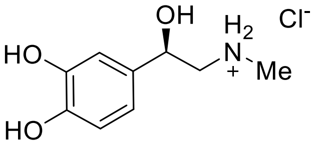	Weight-by-volume conc.	pH
	10%	2.72
	5%	3.91
	2%	4.84
Epinephrine acesulfamate **6a**	Weight-by-volume conc.	pH
	10%	4.01

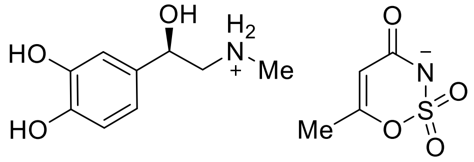	5%	4.14
	2%	4.34
Epinephrine saccharinate **6b**	Weight-by-volume conc.	pH
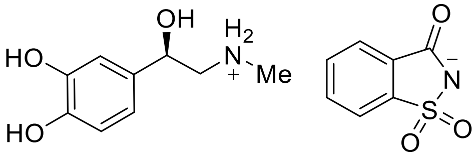	10%	3.57
	5%	4.03
	2%	4.41

^{1}H spectrum for mepivacaine acesulfamate 3a

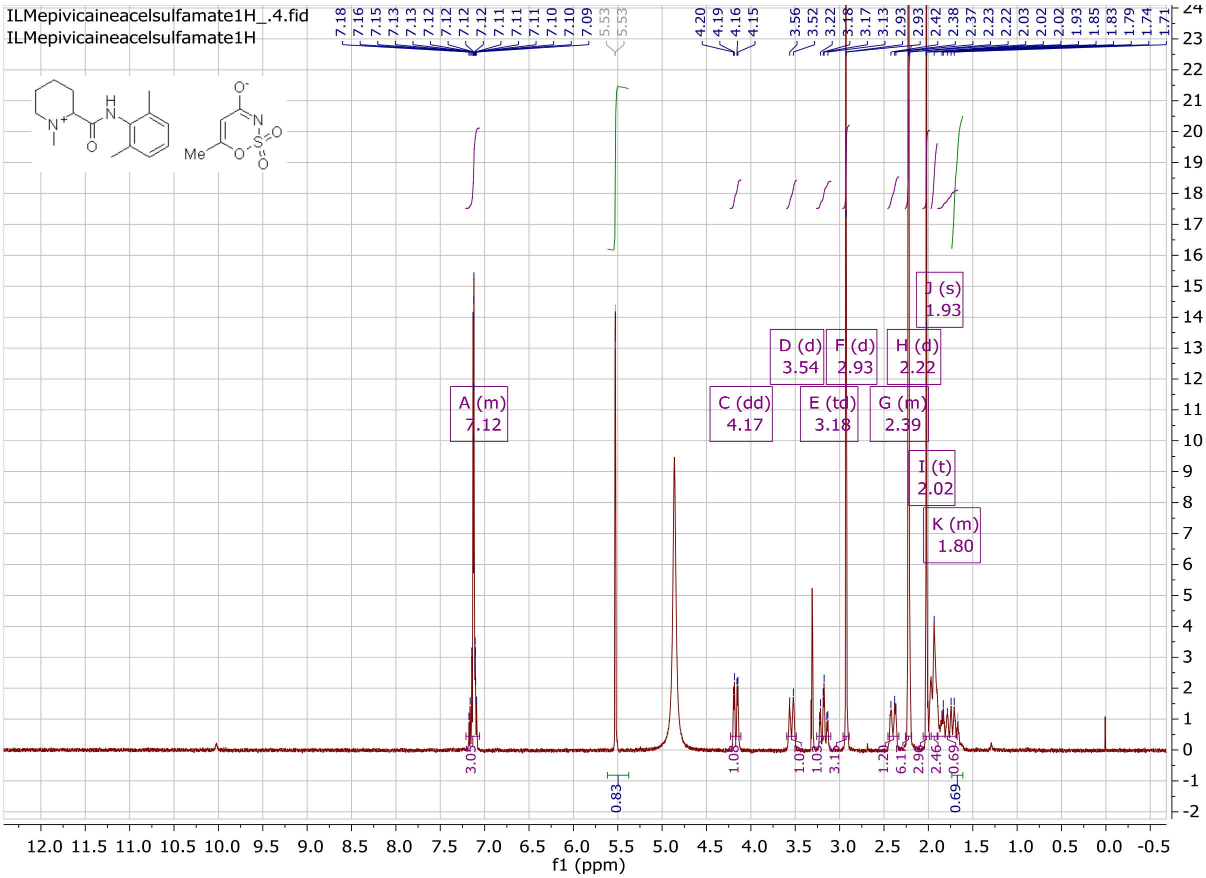

^{13}C spectrum for mepivacaine acesulfamate 3a

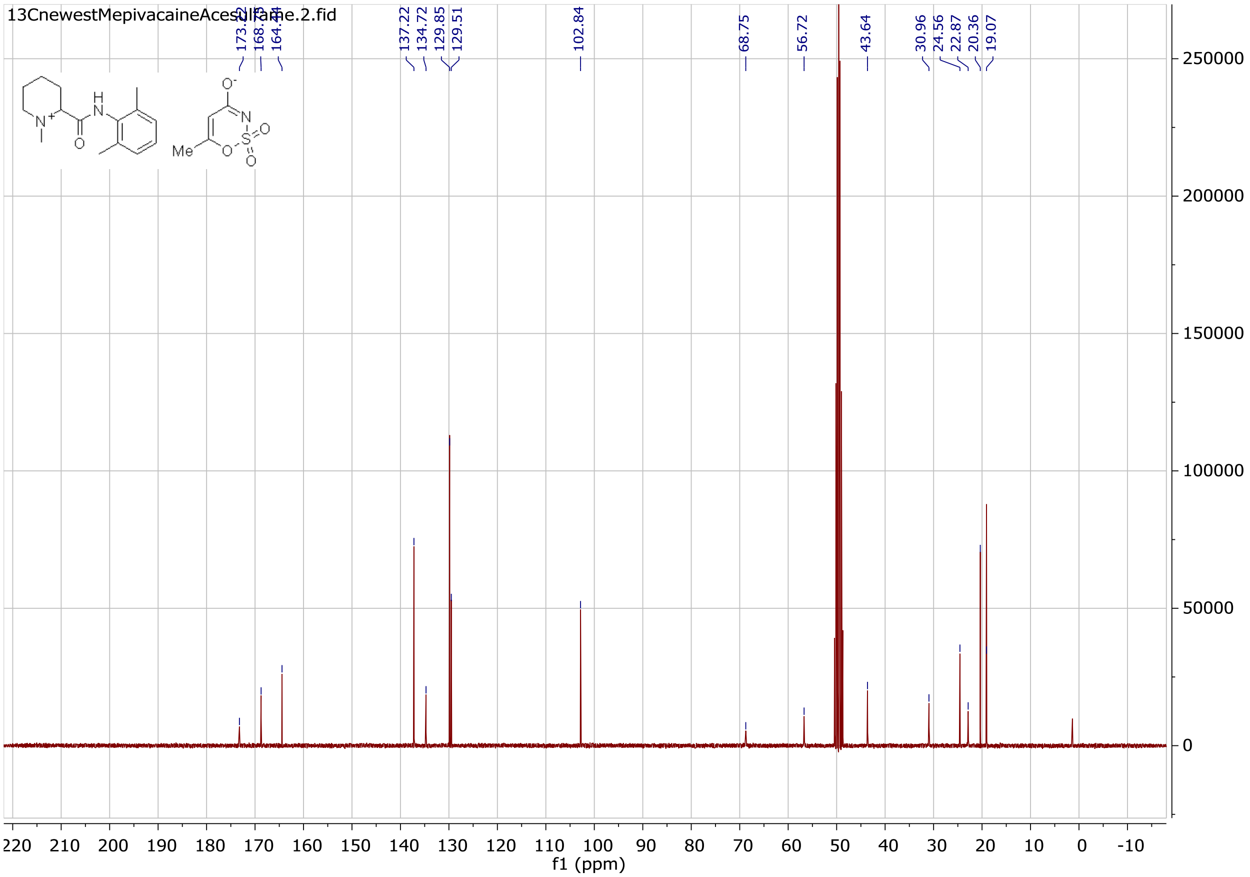

HRMS data for mepivacaine acesulfamate 3a

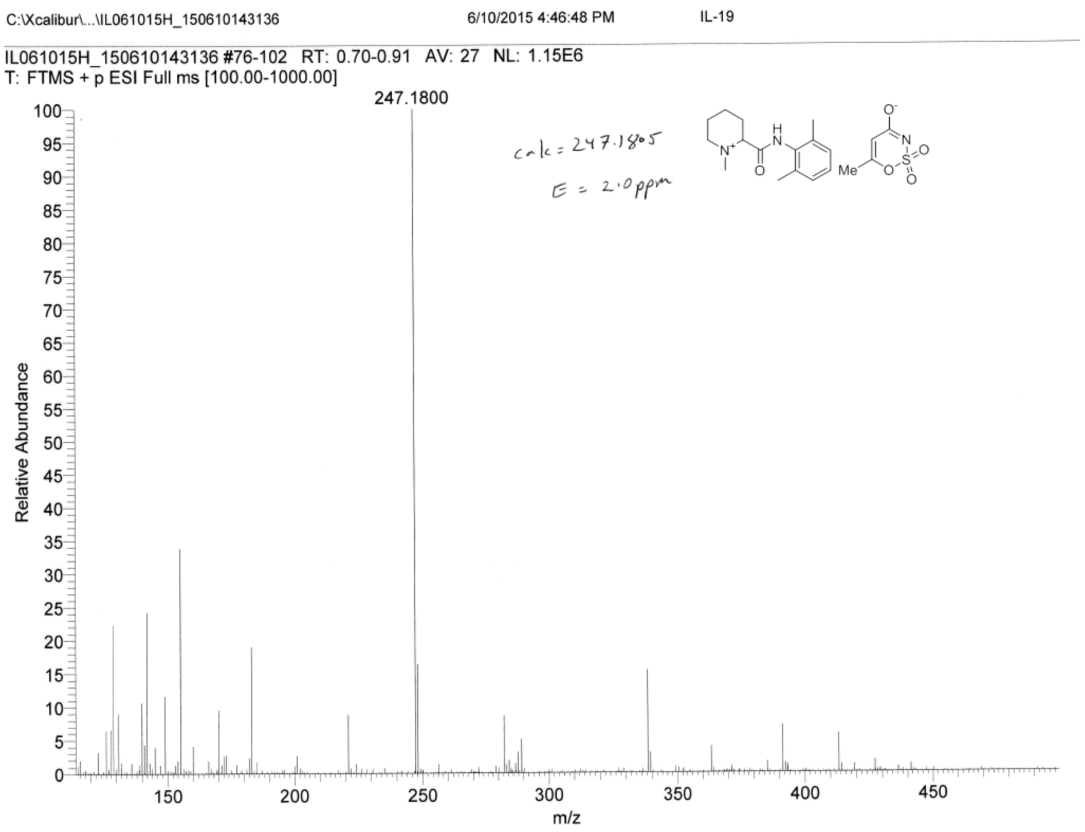

HRMS data for mepivacaine acesulfamate 3a

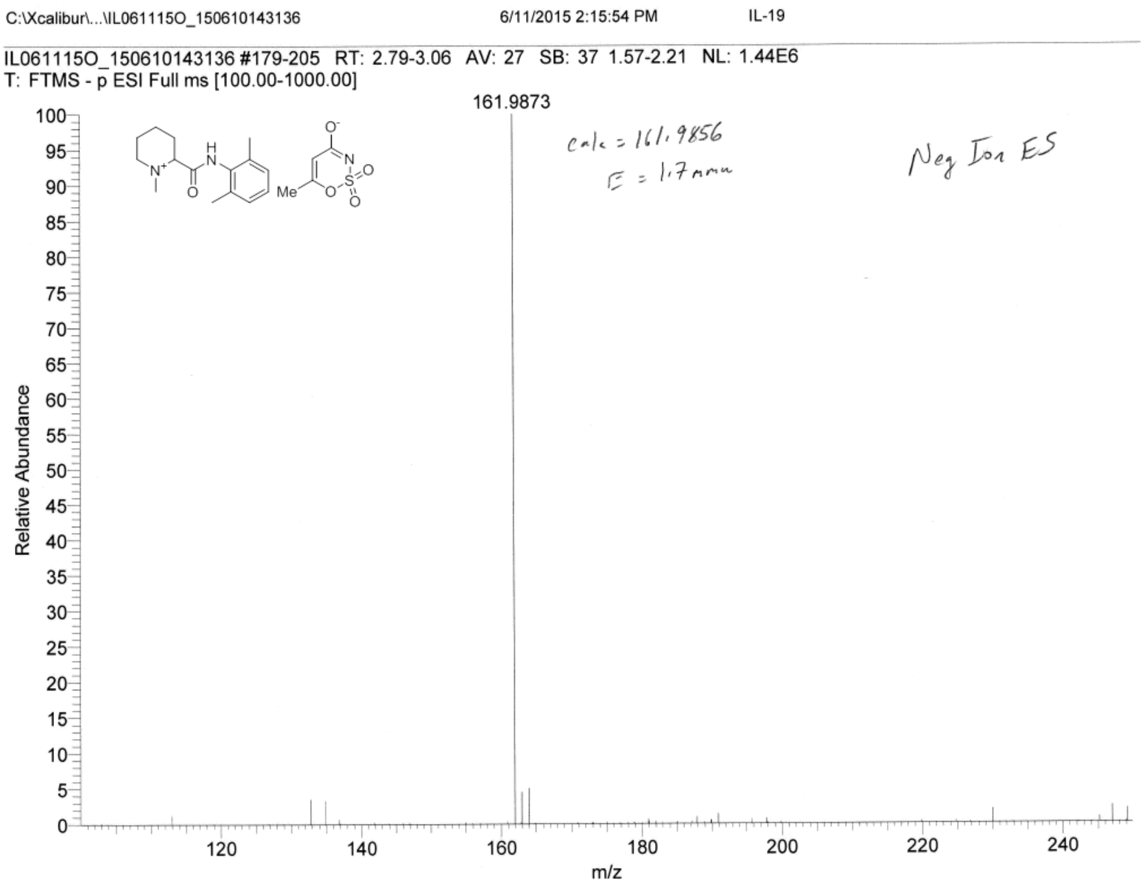

^{1}H spectrum for bupivacaine acesulfamate 3b

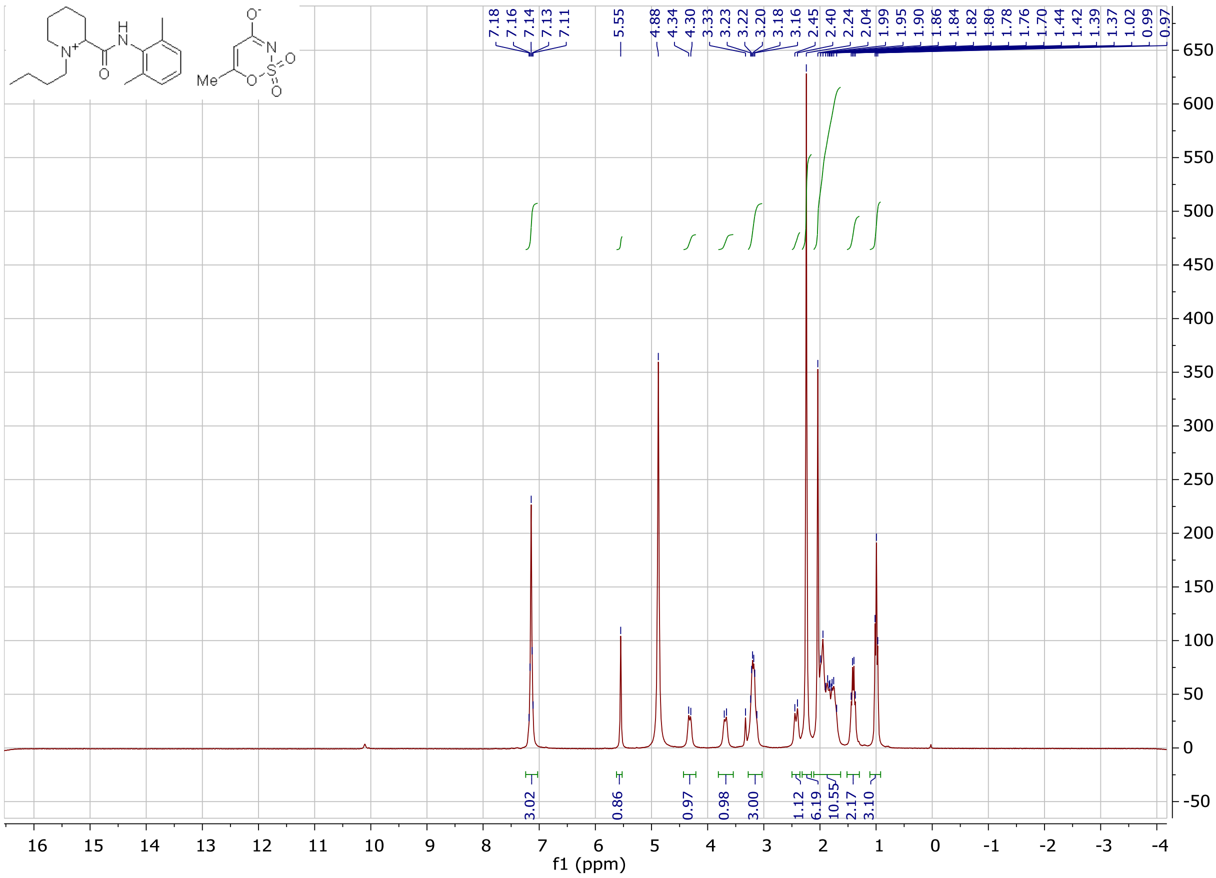

^{13}C spectrum for bupivacaine acesulfamate 3b

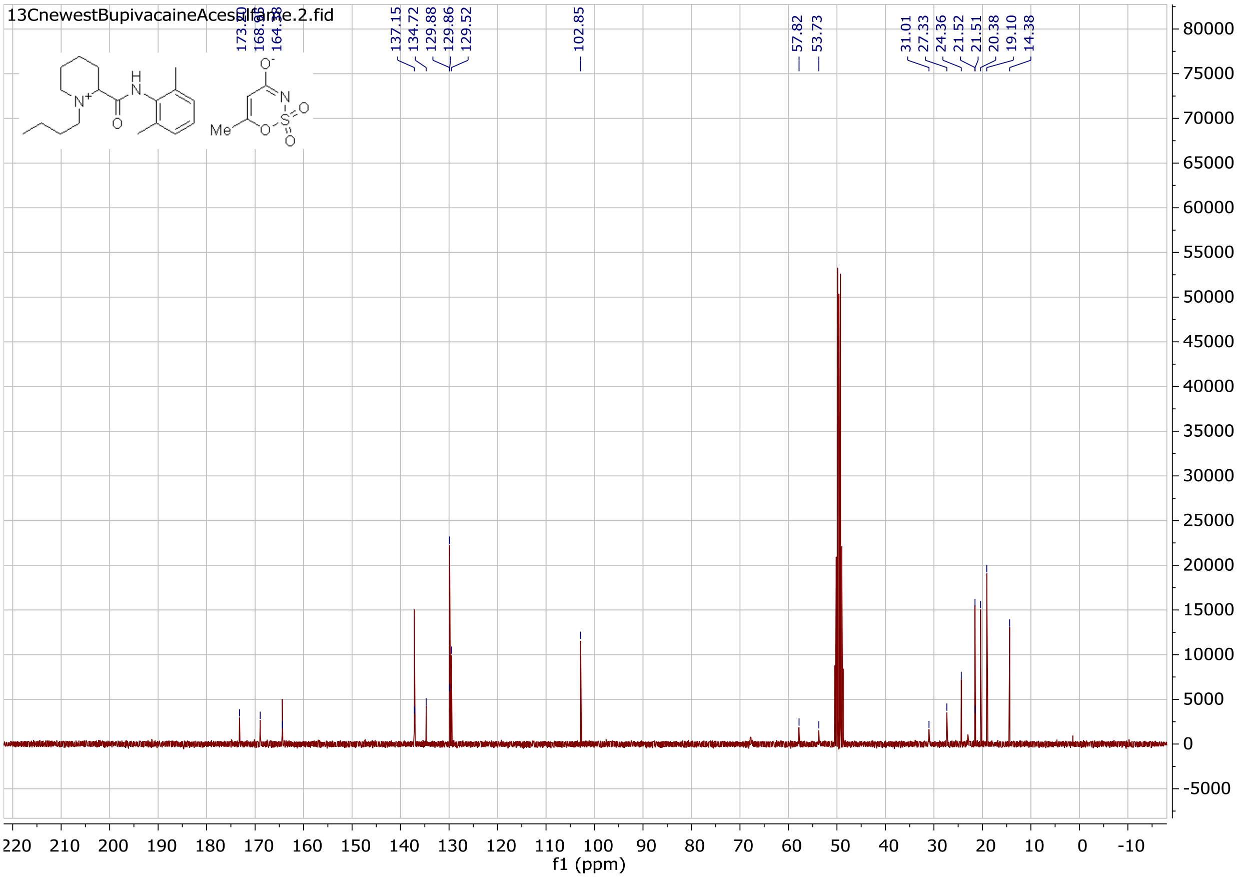

HRMS data for bupivacaine acesulfamate 3b

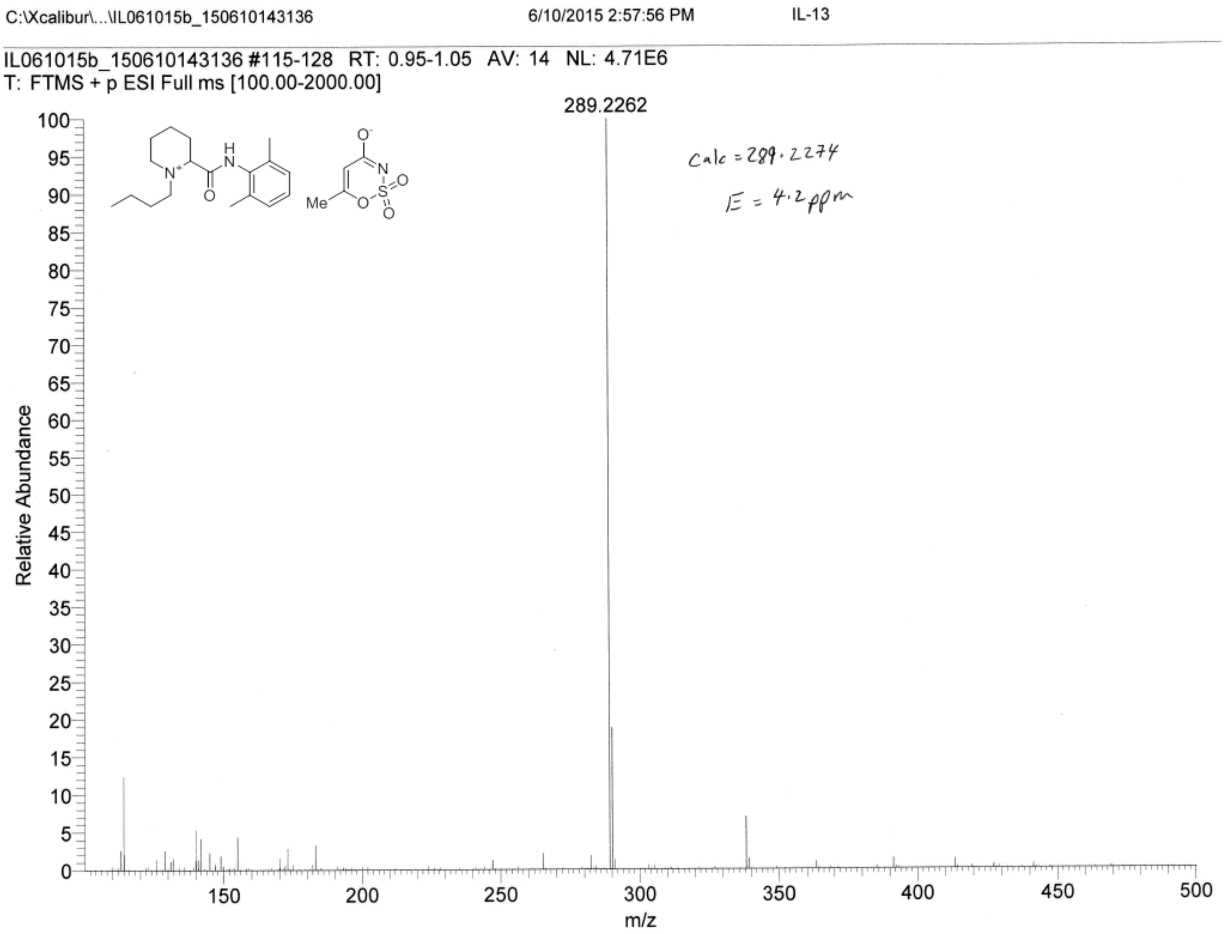

HRMS data for bupivacaine acesulfamate 3b

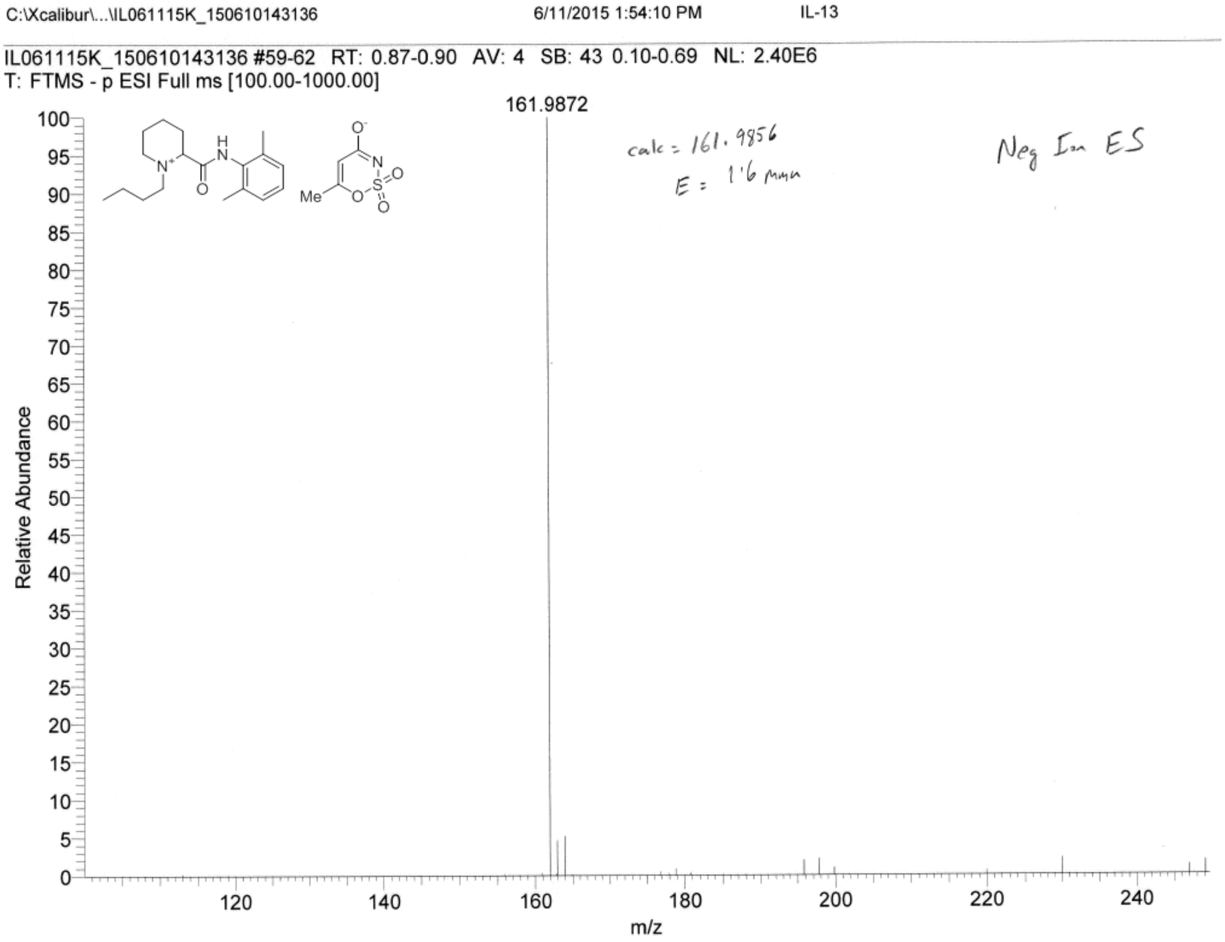

^{1}H spectrum for prilocaine acesulfamate 3c

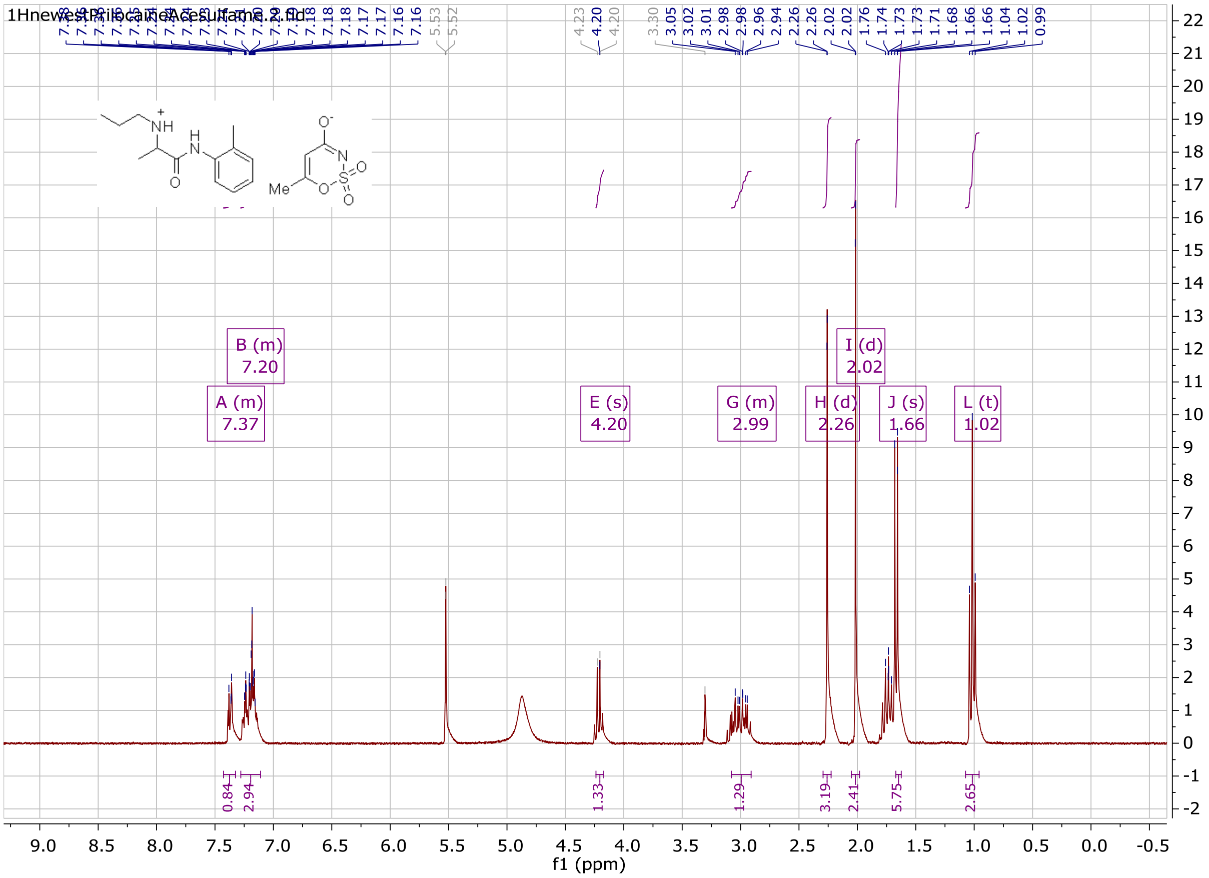

^{13}C spectrum for prilocaine acesulfamate 3c

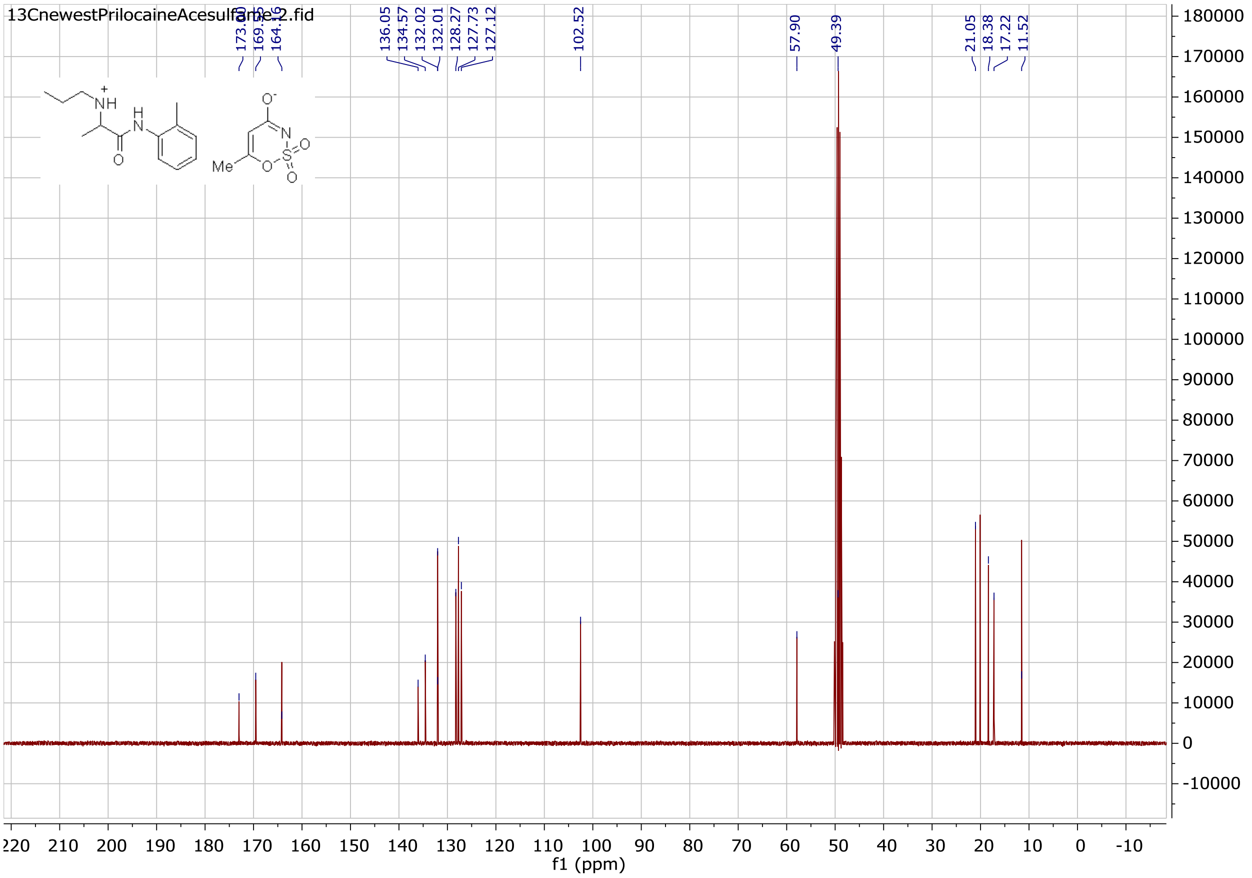

HRMS data for prilocaine acesulfamate 3c

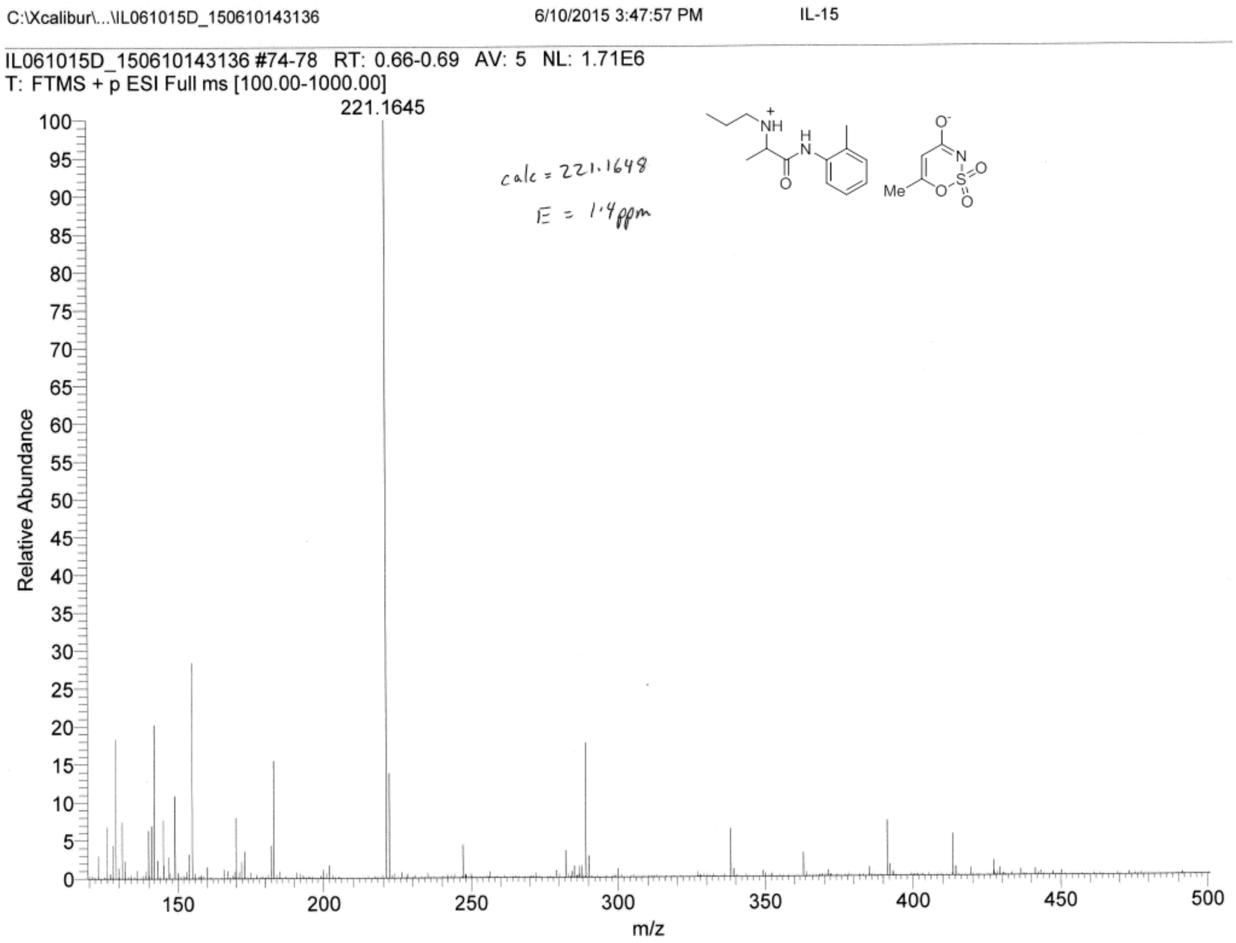

HRMS data for prilocaine acesulfamate 3c

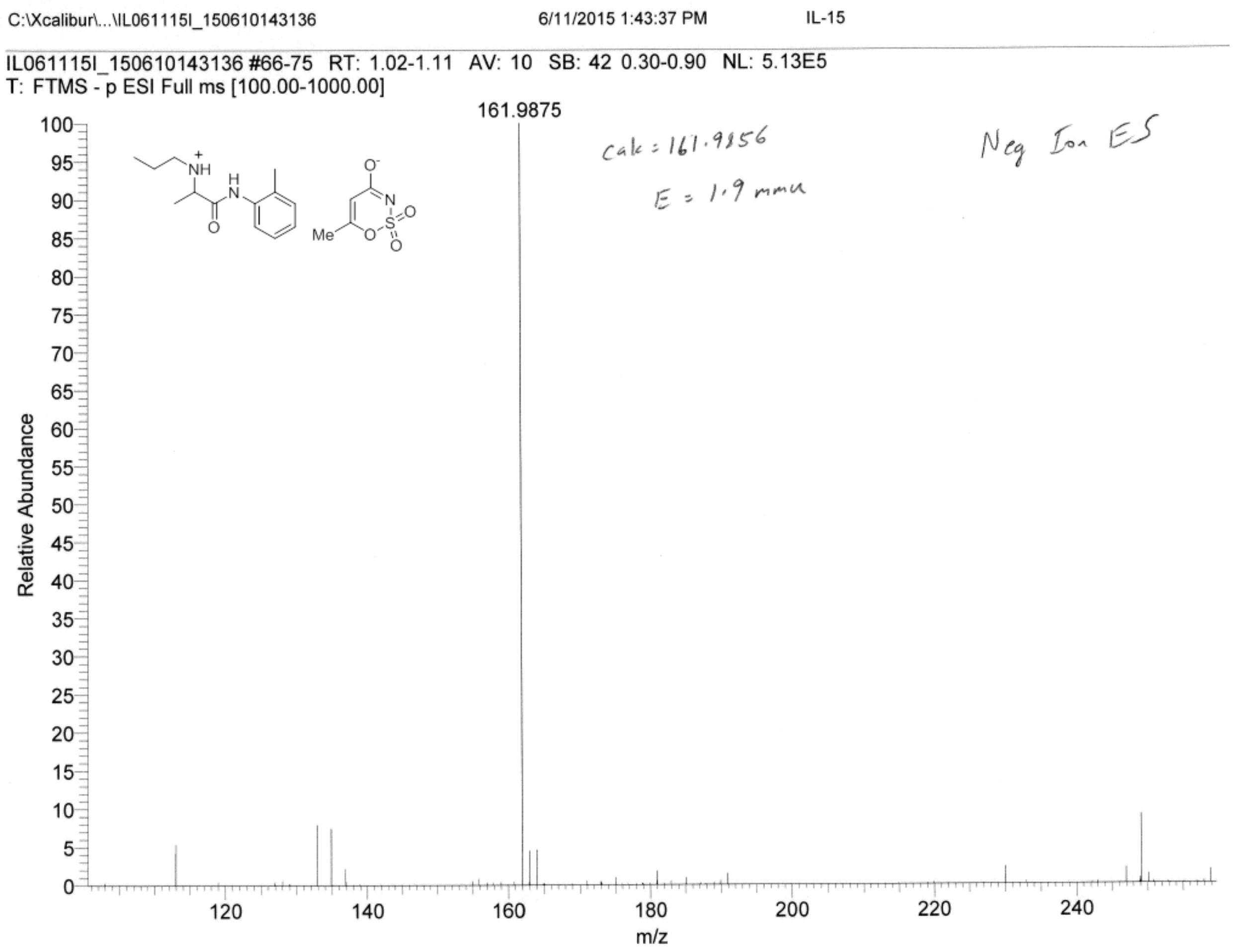

^{1}H spectrum for articaine acesulfamate 3d

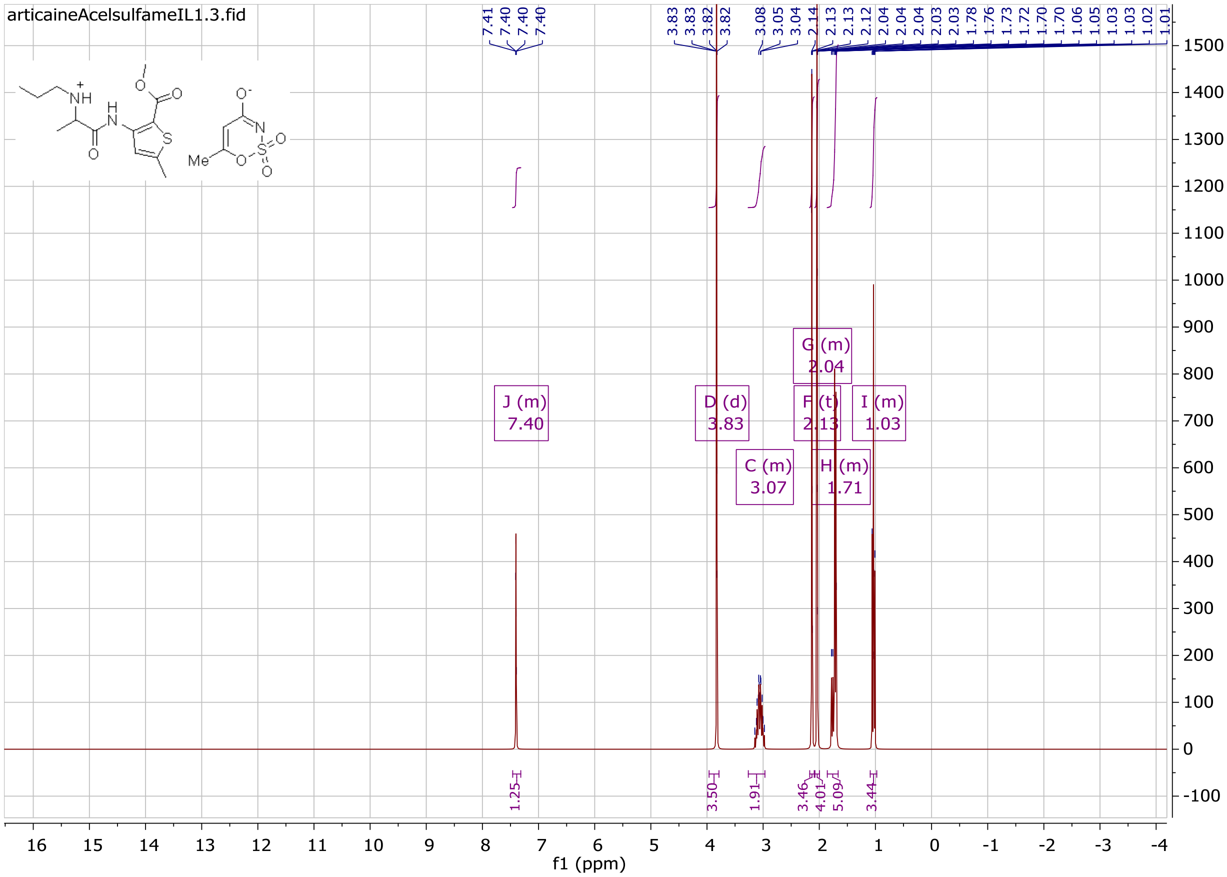

^{13}C spectrum for articaine acesulfamate 3d

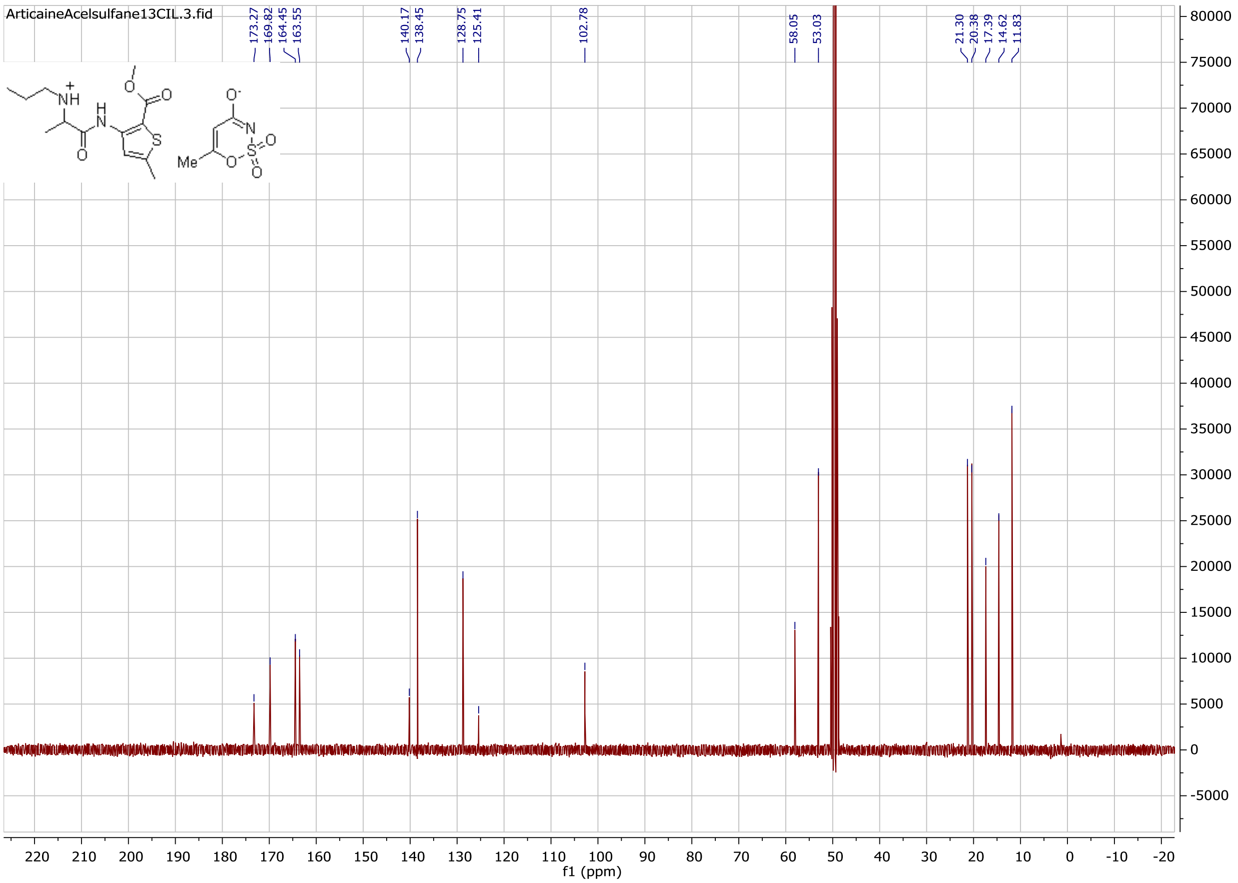

HRMS data for articaine acesulfamate 3d

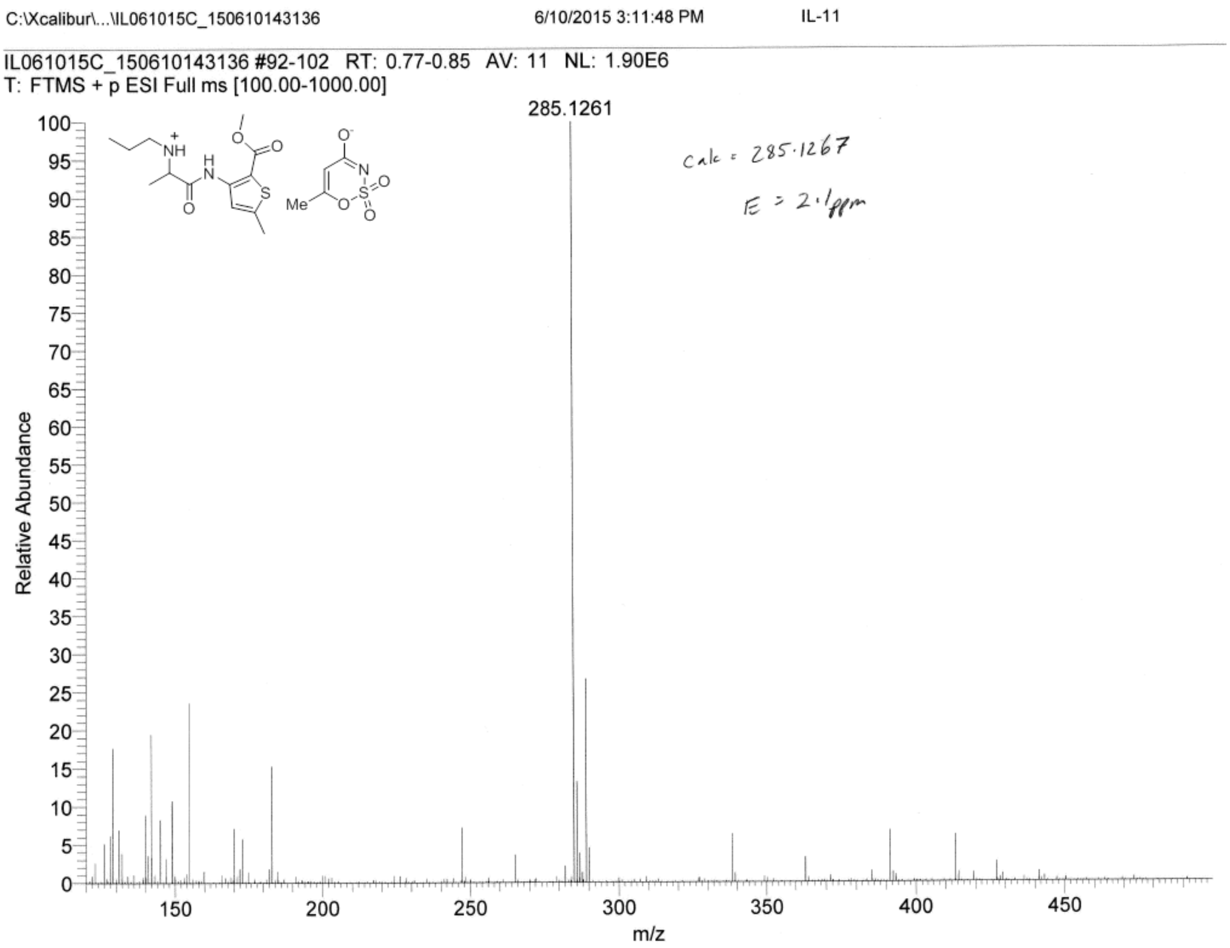

HRMS data for articaine acesulfamate 3d

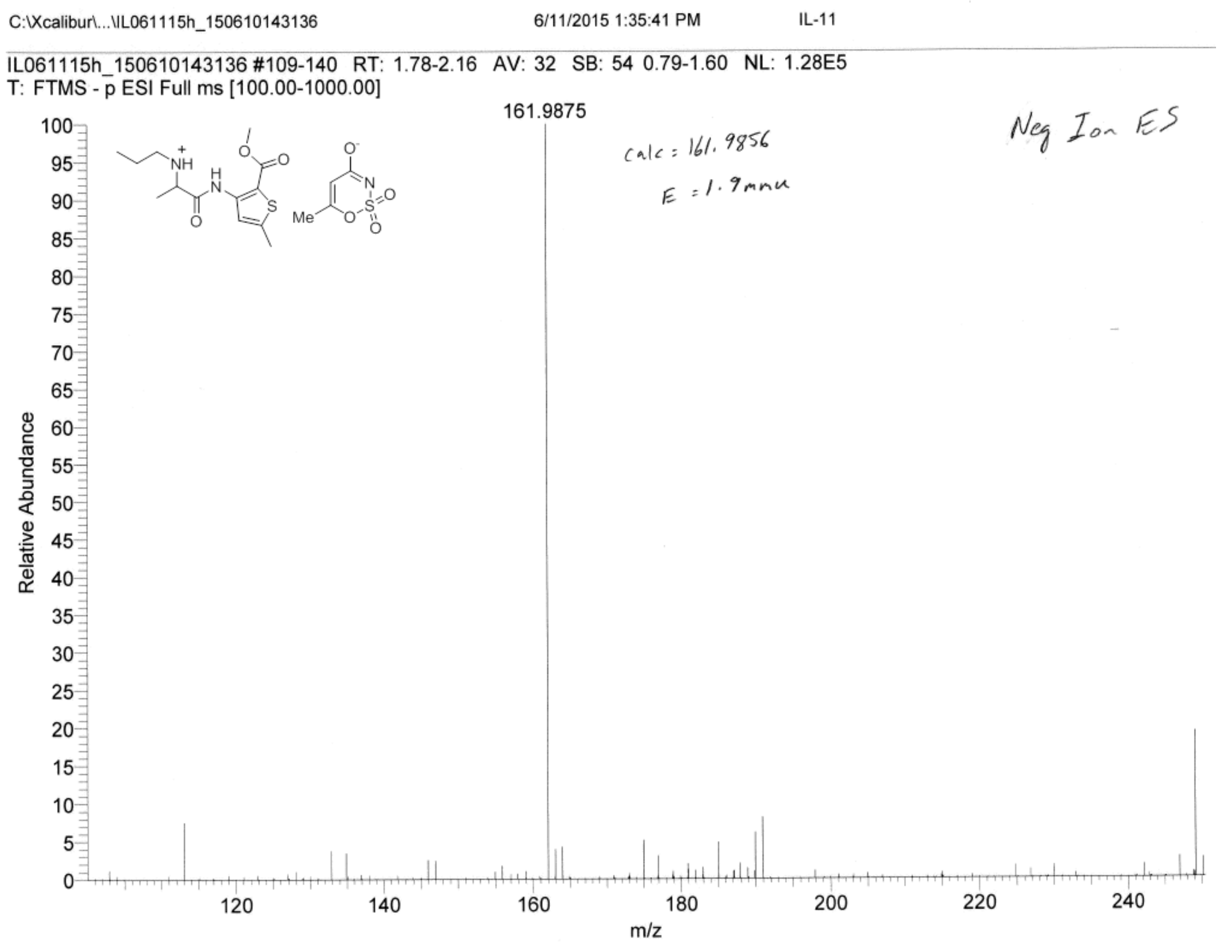

^{1}H spectrum for oxybuprocaine acesulfame 3e

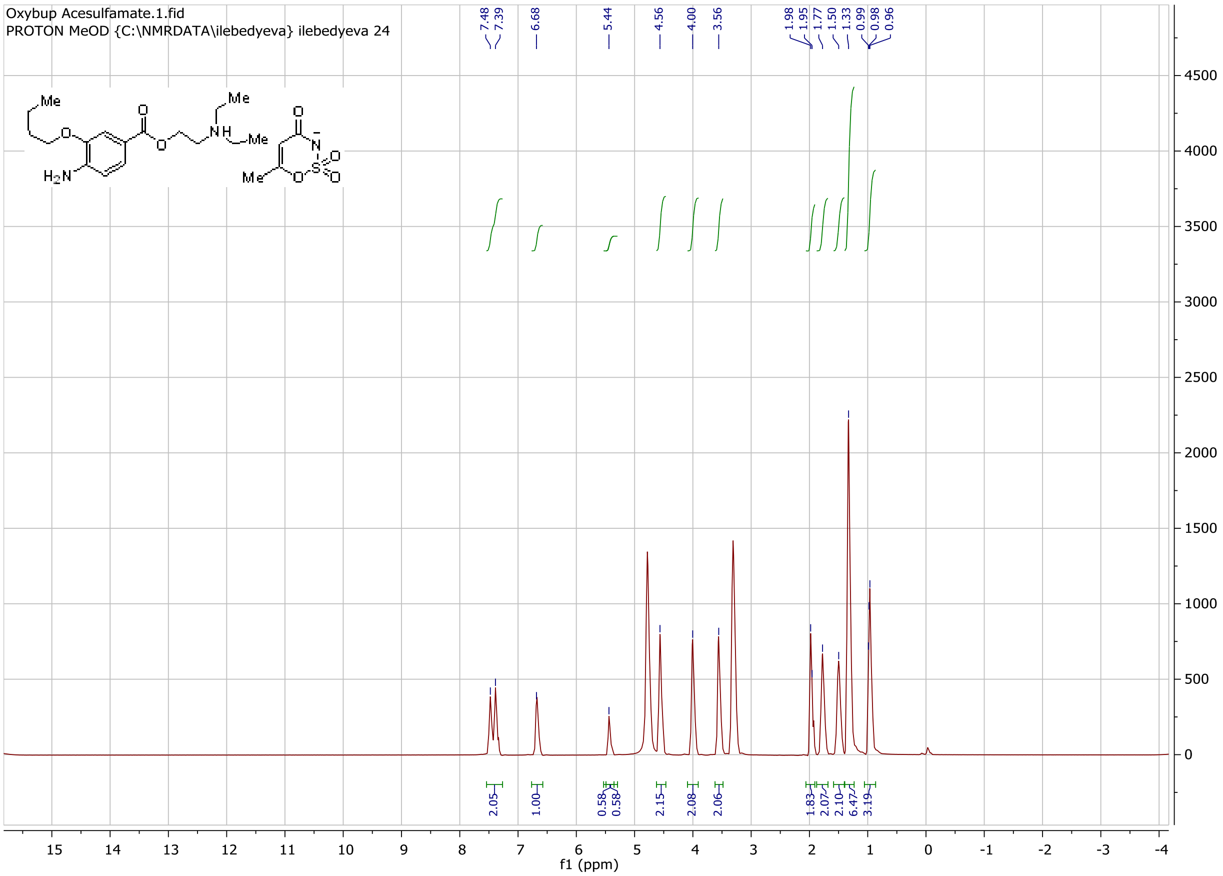

^{13}C spectrum for oxybuprocaine acesulfame 3e

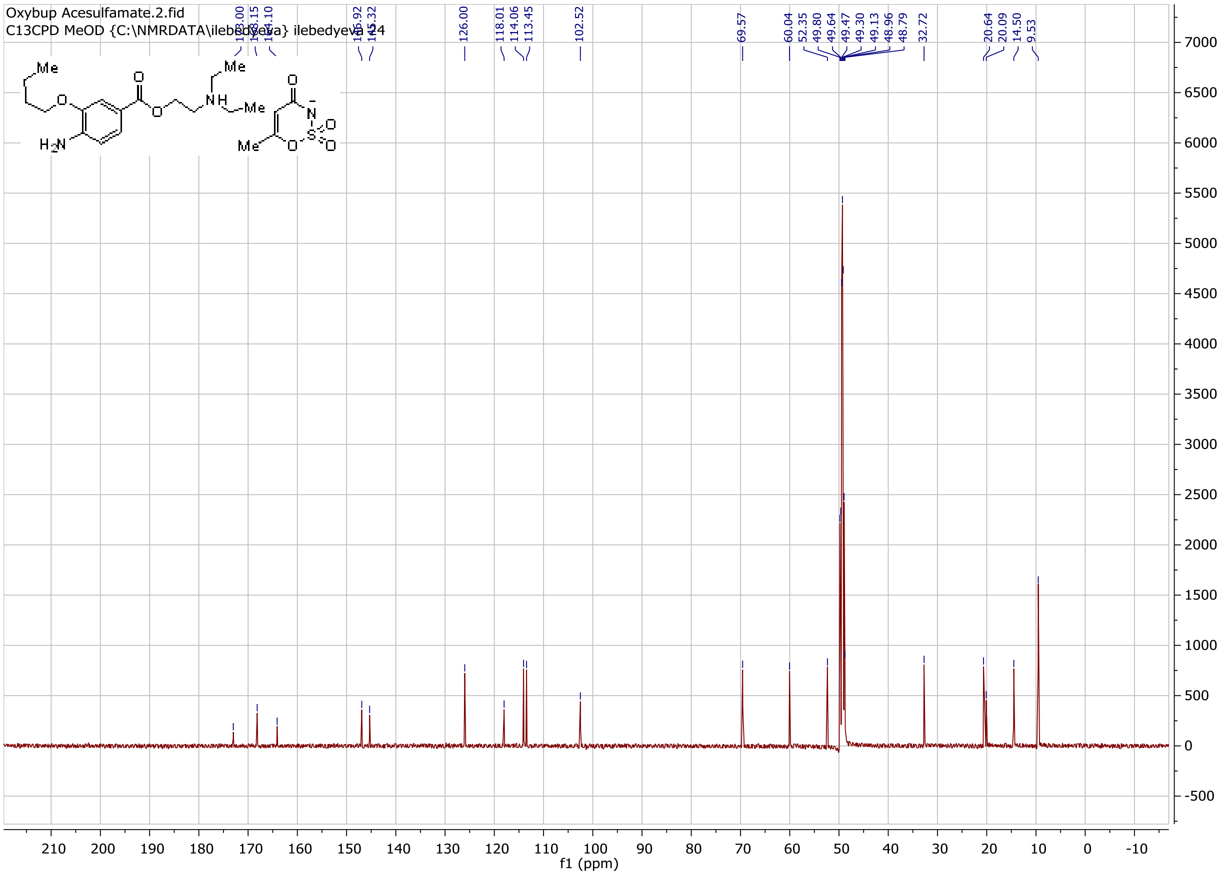

HRMS data for oxybuprocaine acesulfame 3e

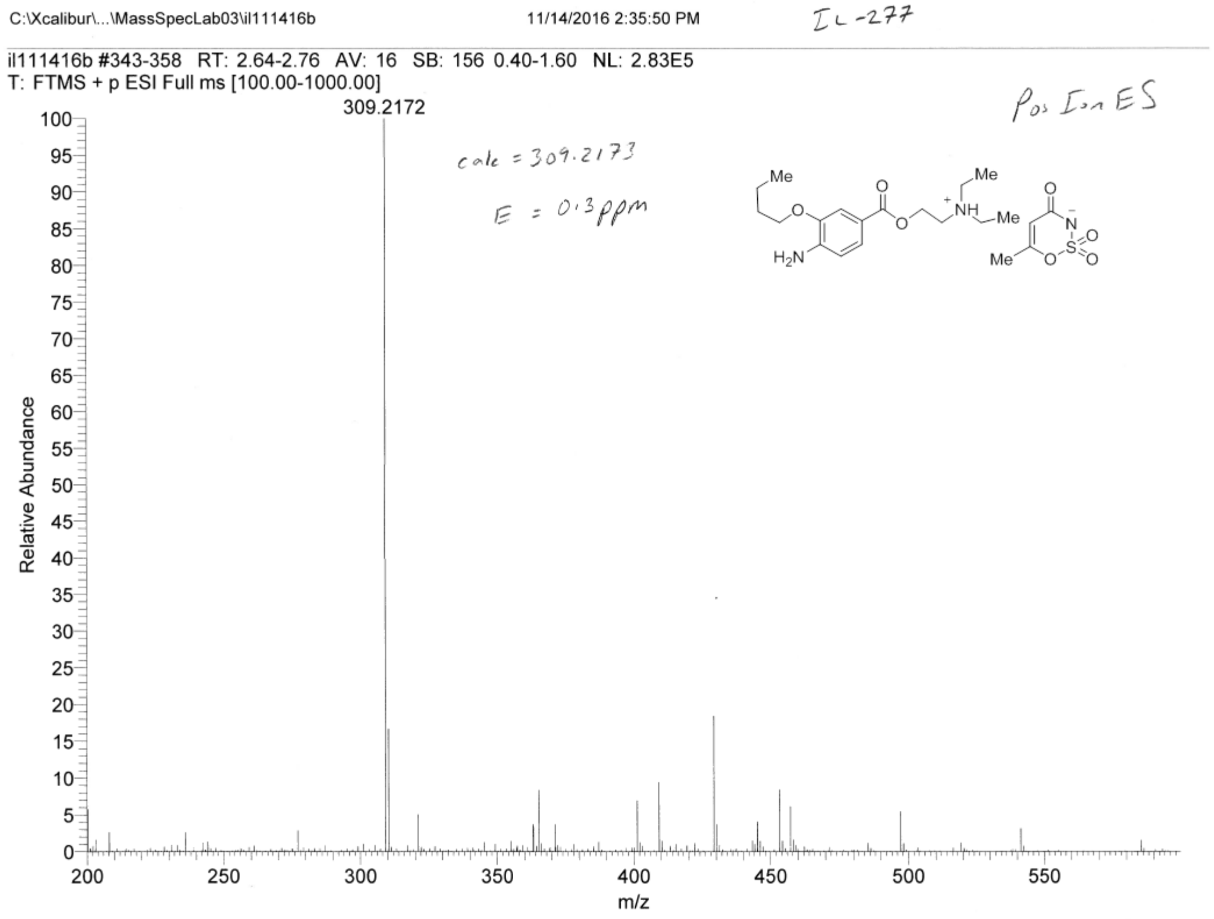

HRMS data for oxybuprocaine acesulfame 3e

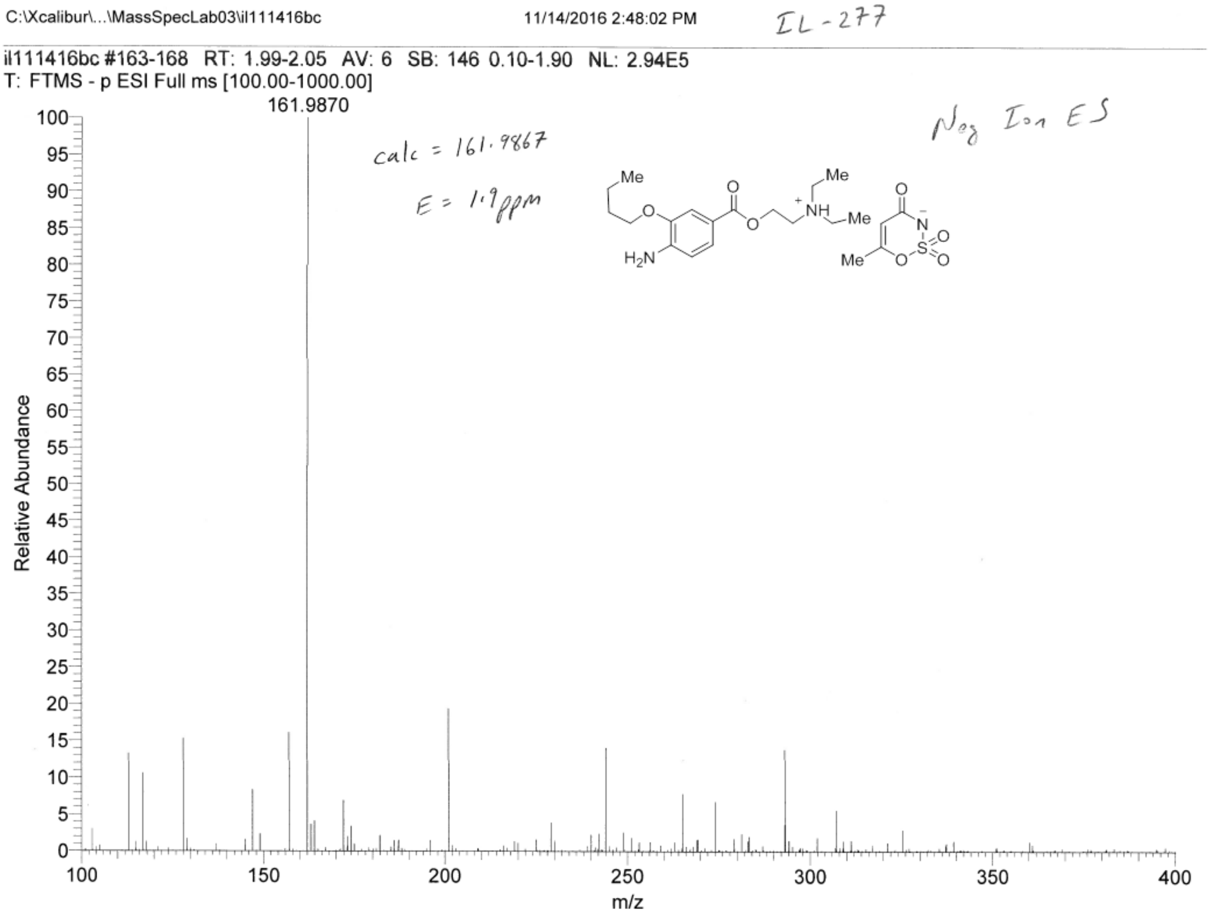

^{1}H spectrum for mepivacaine saccharinate 4a

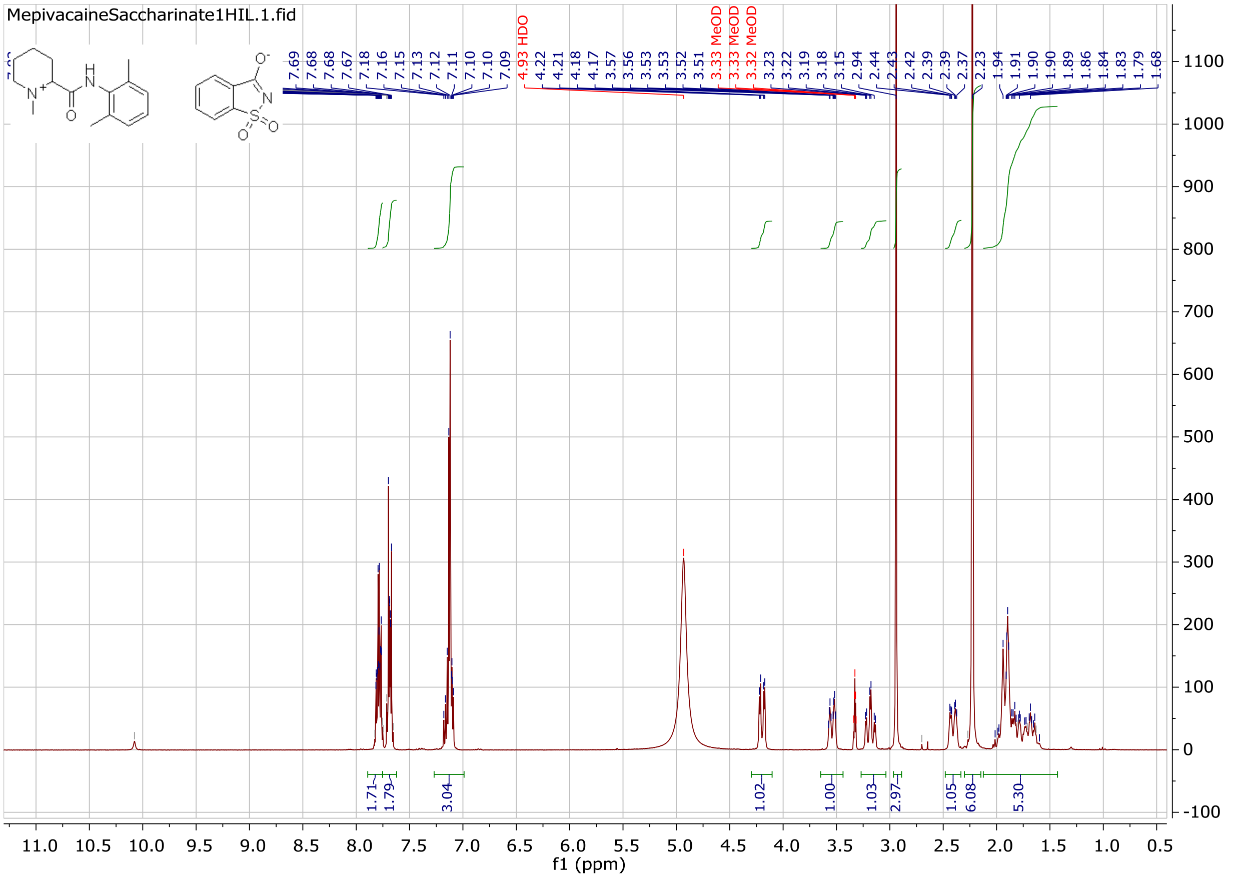

^{13}C spectrum for mepivacaine saccharinate 4a

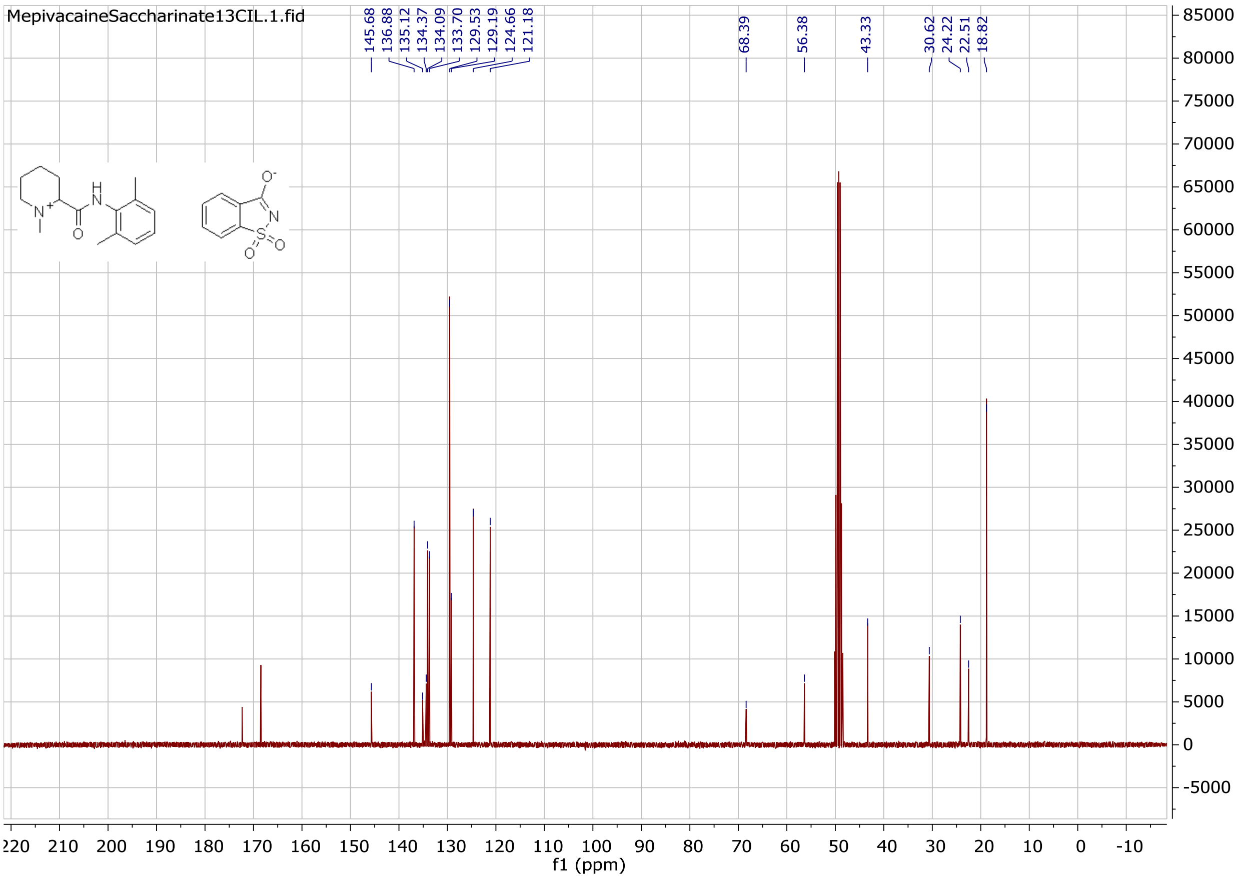

HRMS data for mepivacaine saccharinate 4a

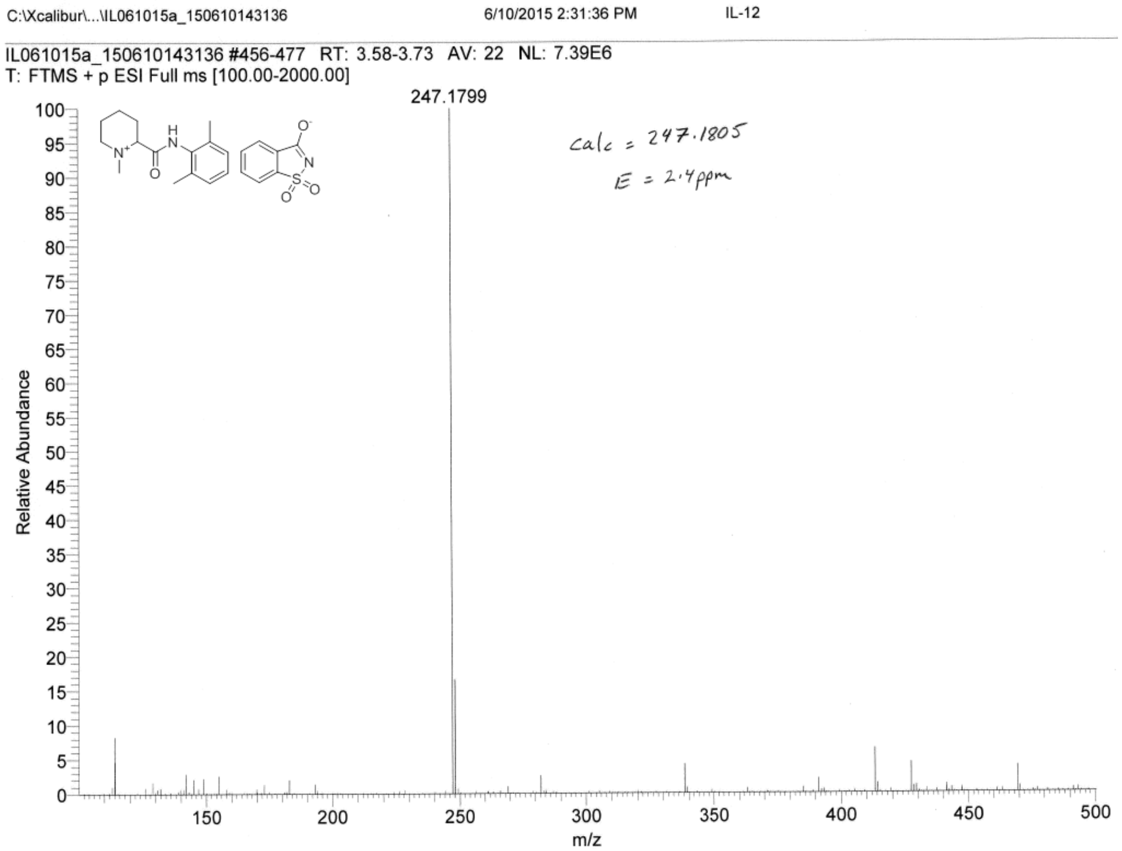

HRMS data for mepivacaine saccharinate 4a

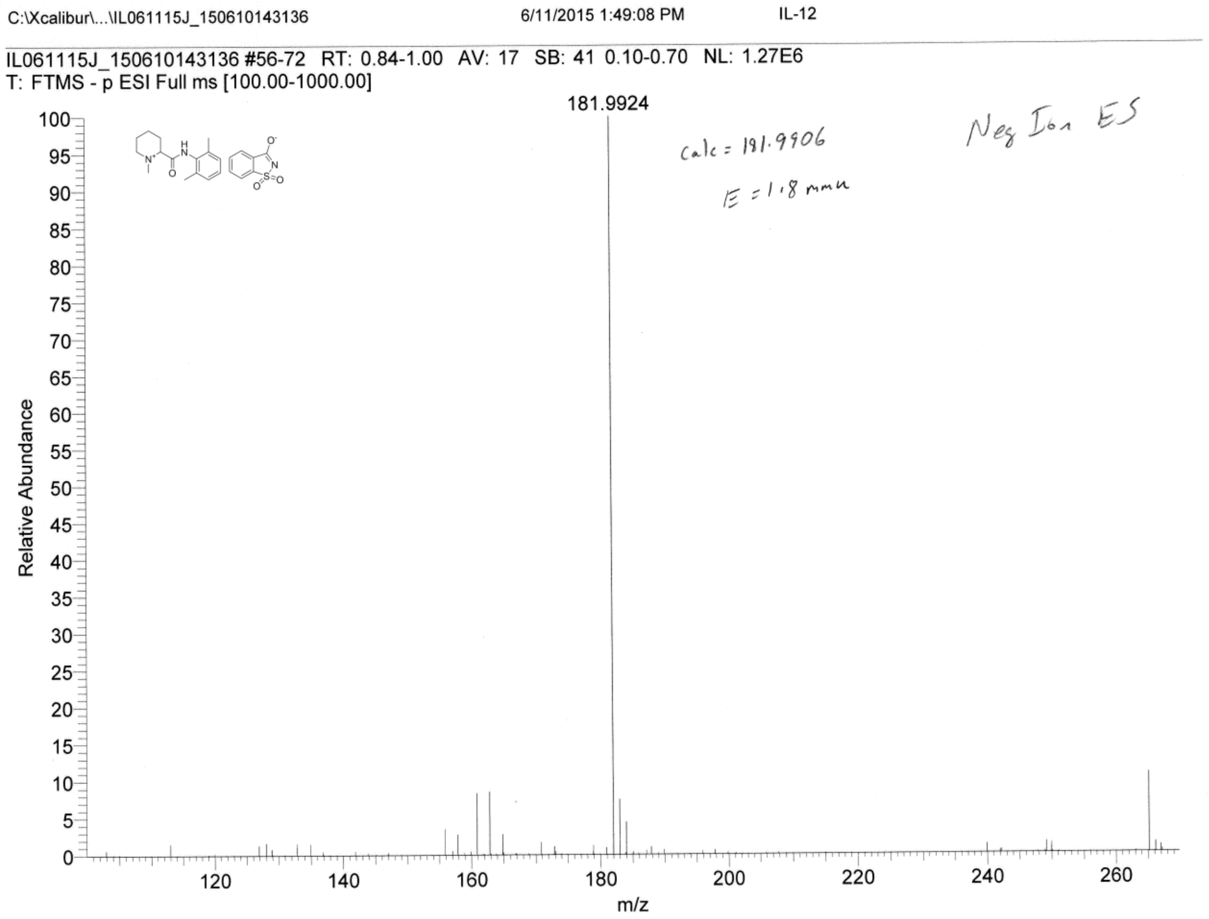

^{1}H spectrum for bupivacaine saccharinate 4b

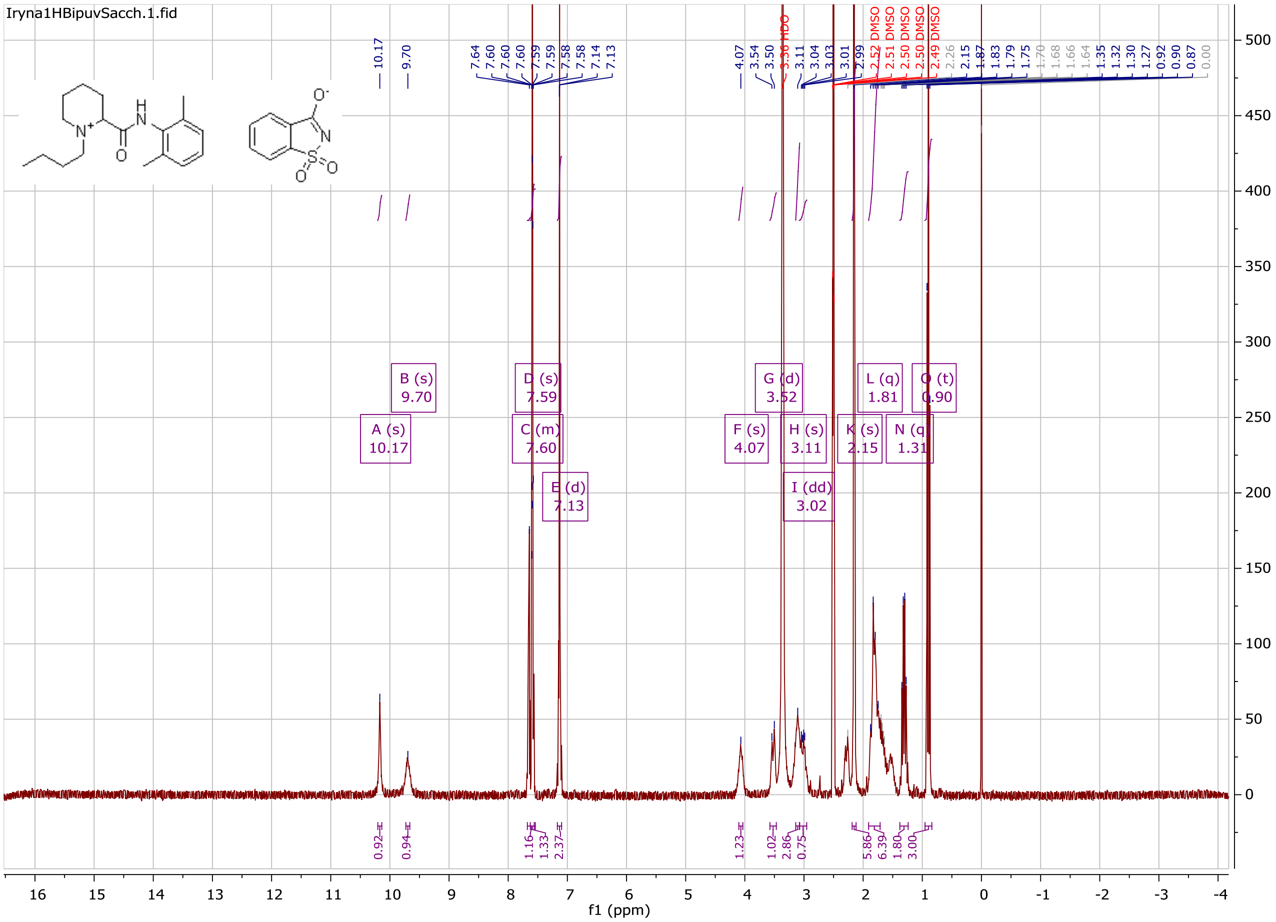

^{13}C spectrum for bupivacaine saccharinate 4b

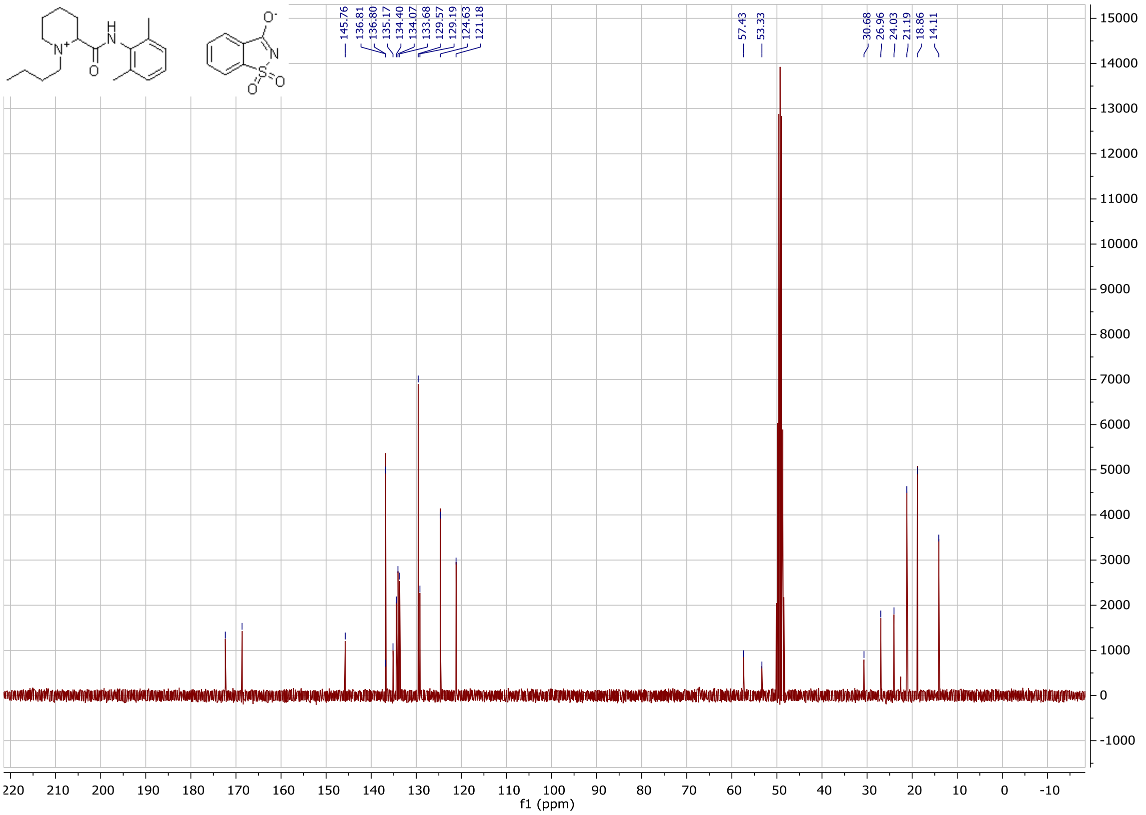

HRMS data for bupivacaine saccharinate 4b

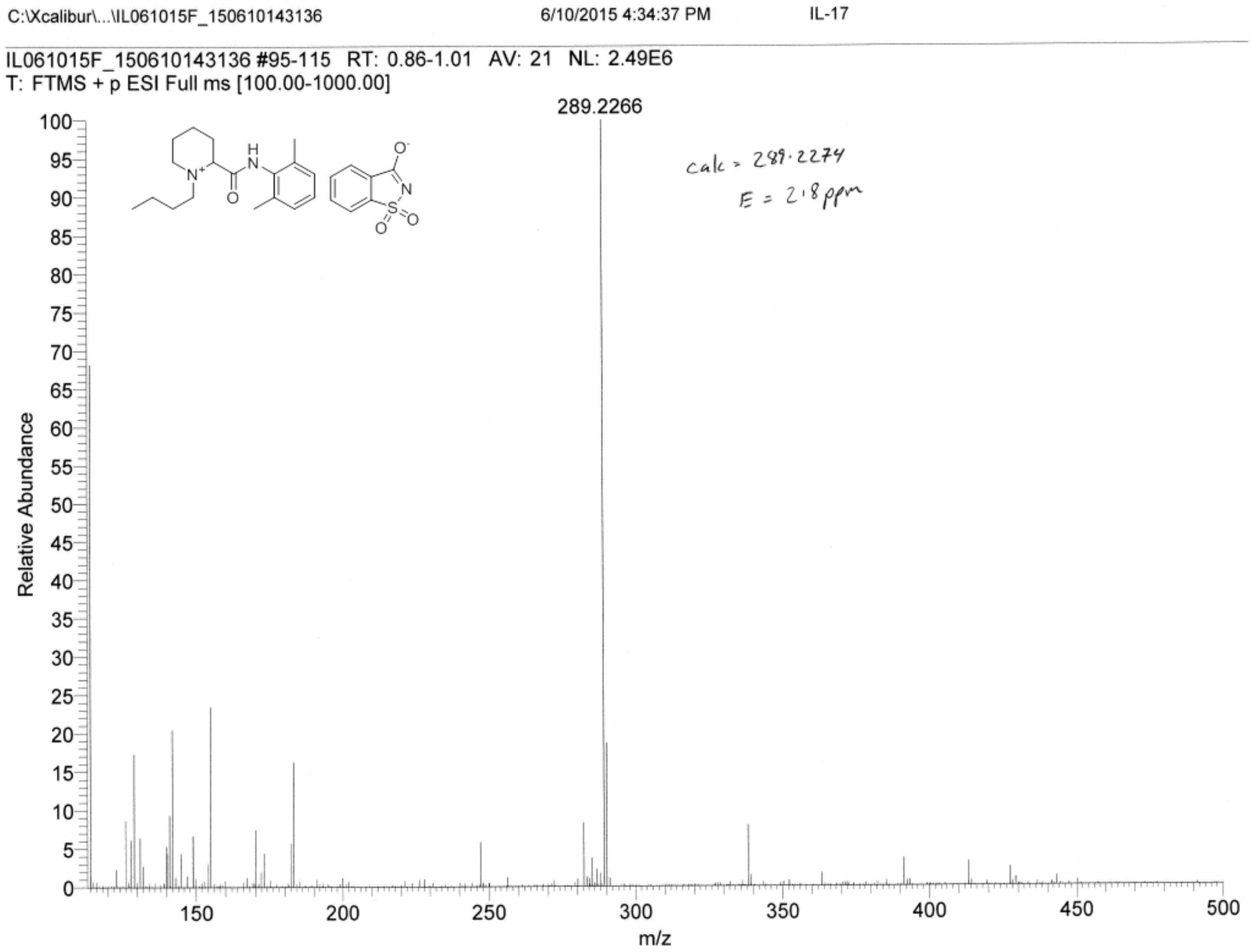

HRMS data for bupivacaine saccharinate 4b

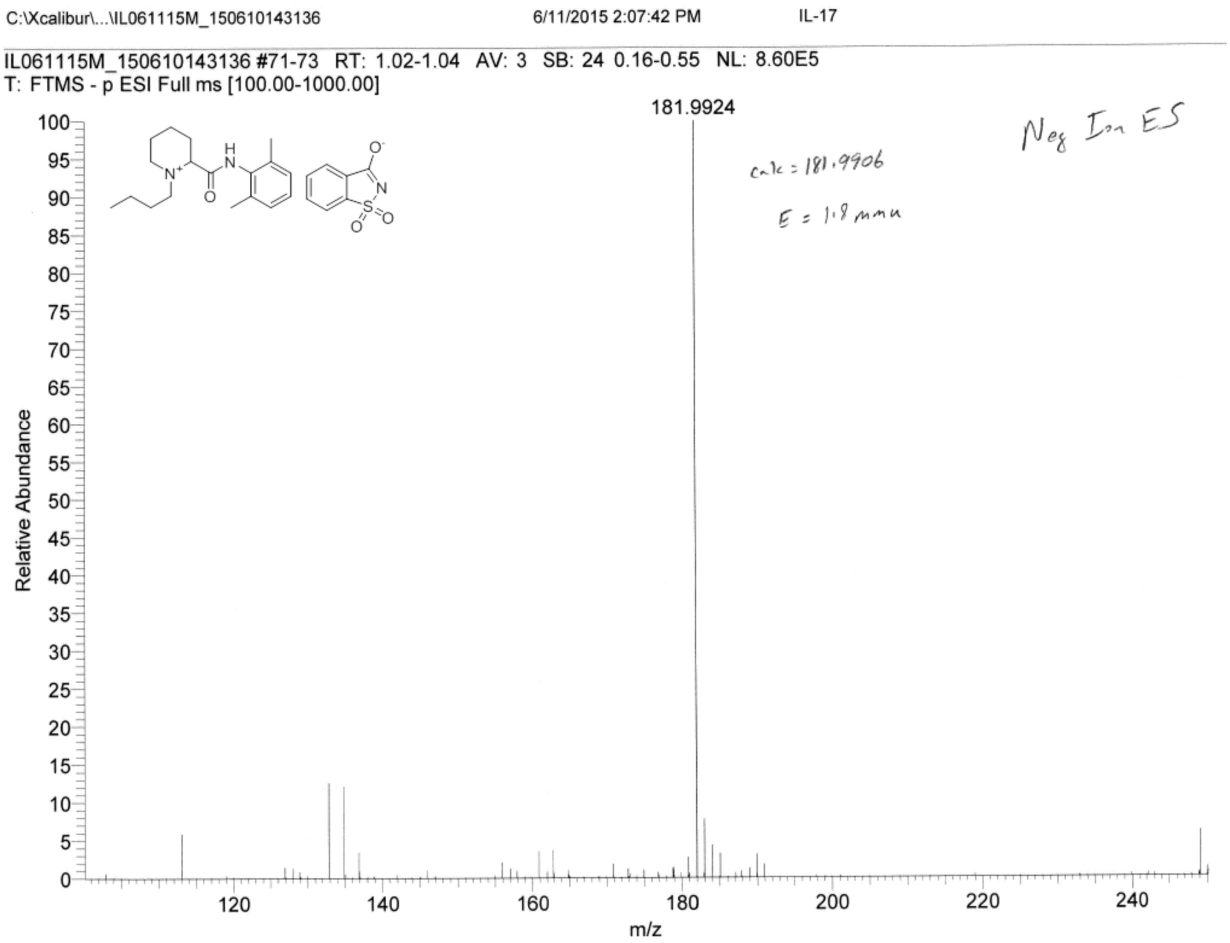

^{1}H spectrum for prilocaine saccharinate 4c

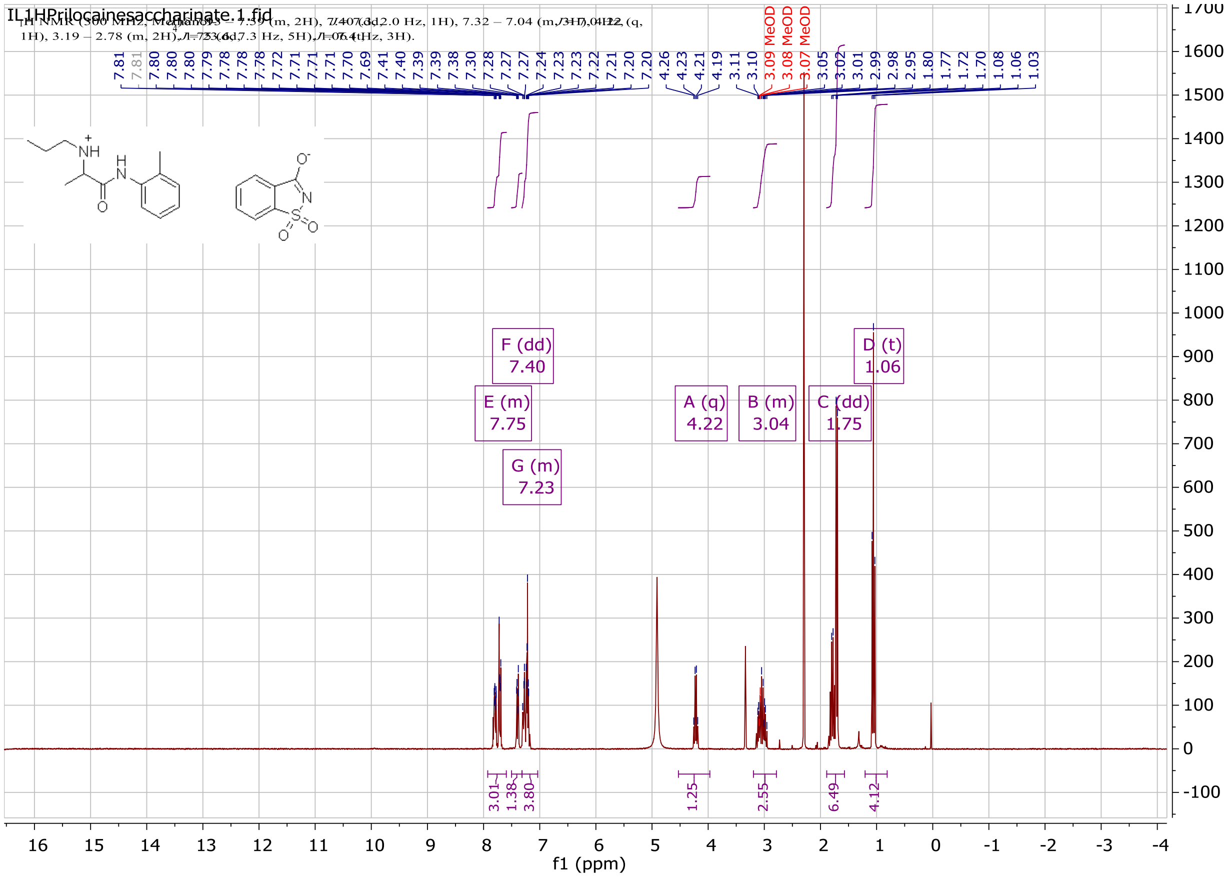

^{13}C spectrum for prilocaine saccharinate 4c

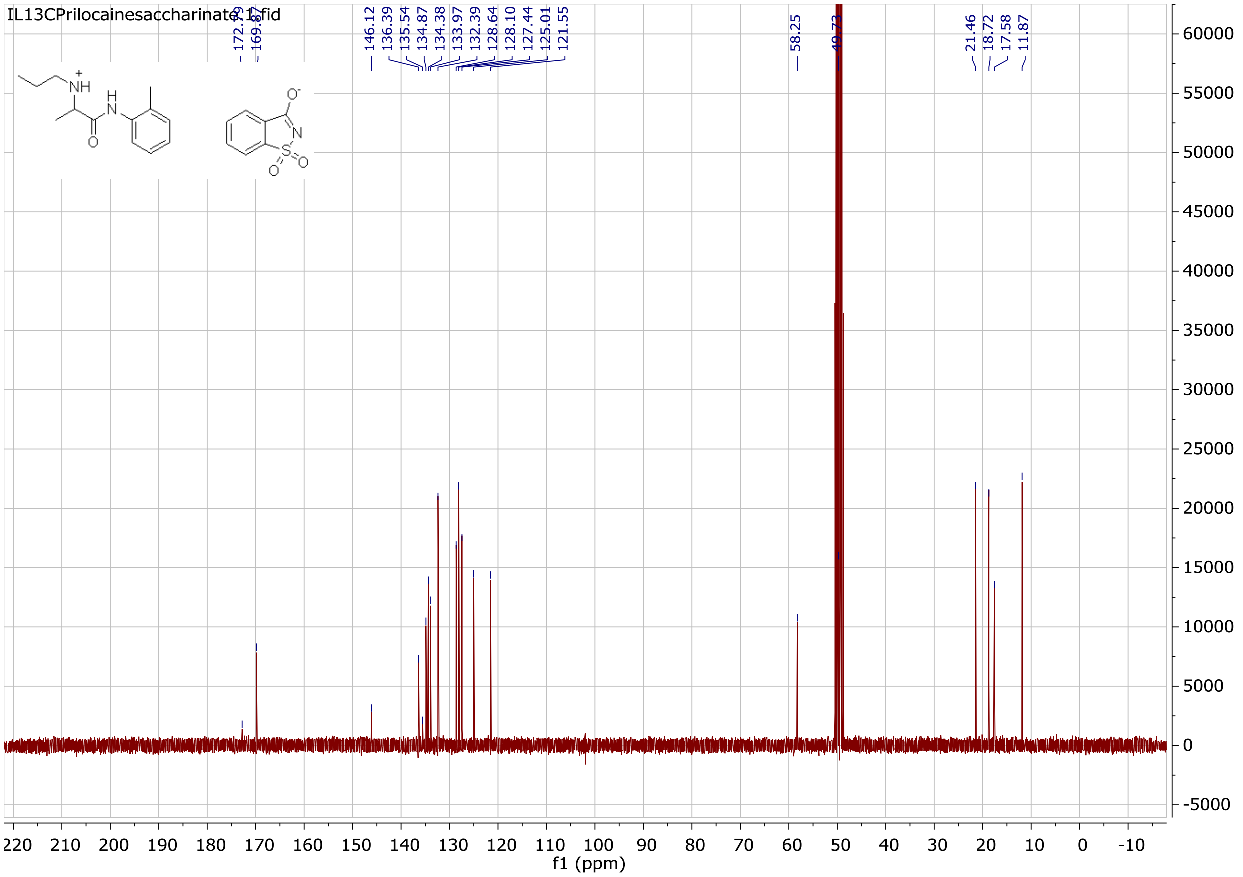

HRMS data for prilocaine saccharinate 4c

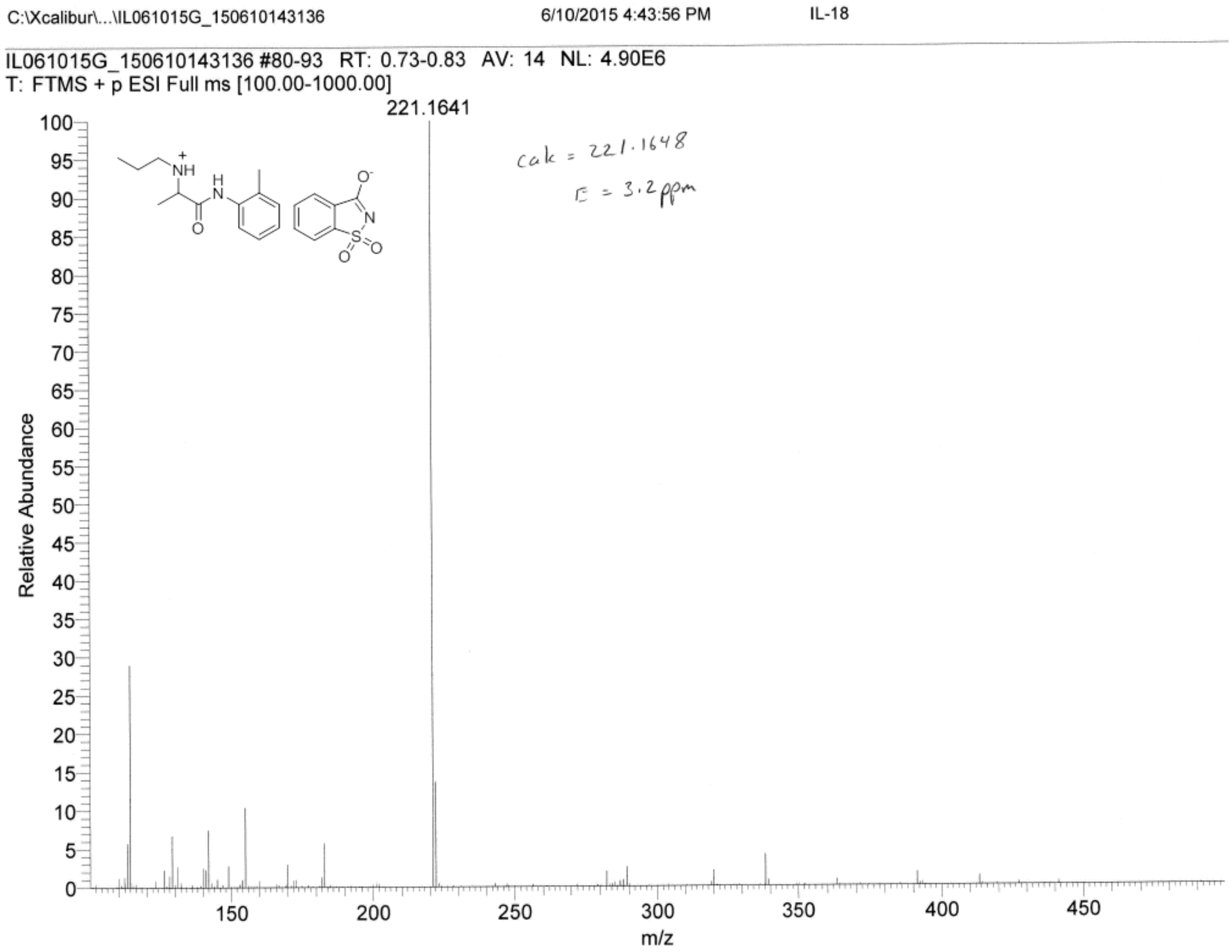

HRMS data for prilocaine saccharinate 4c

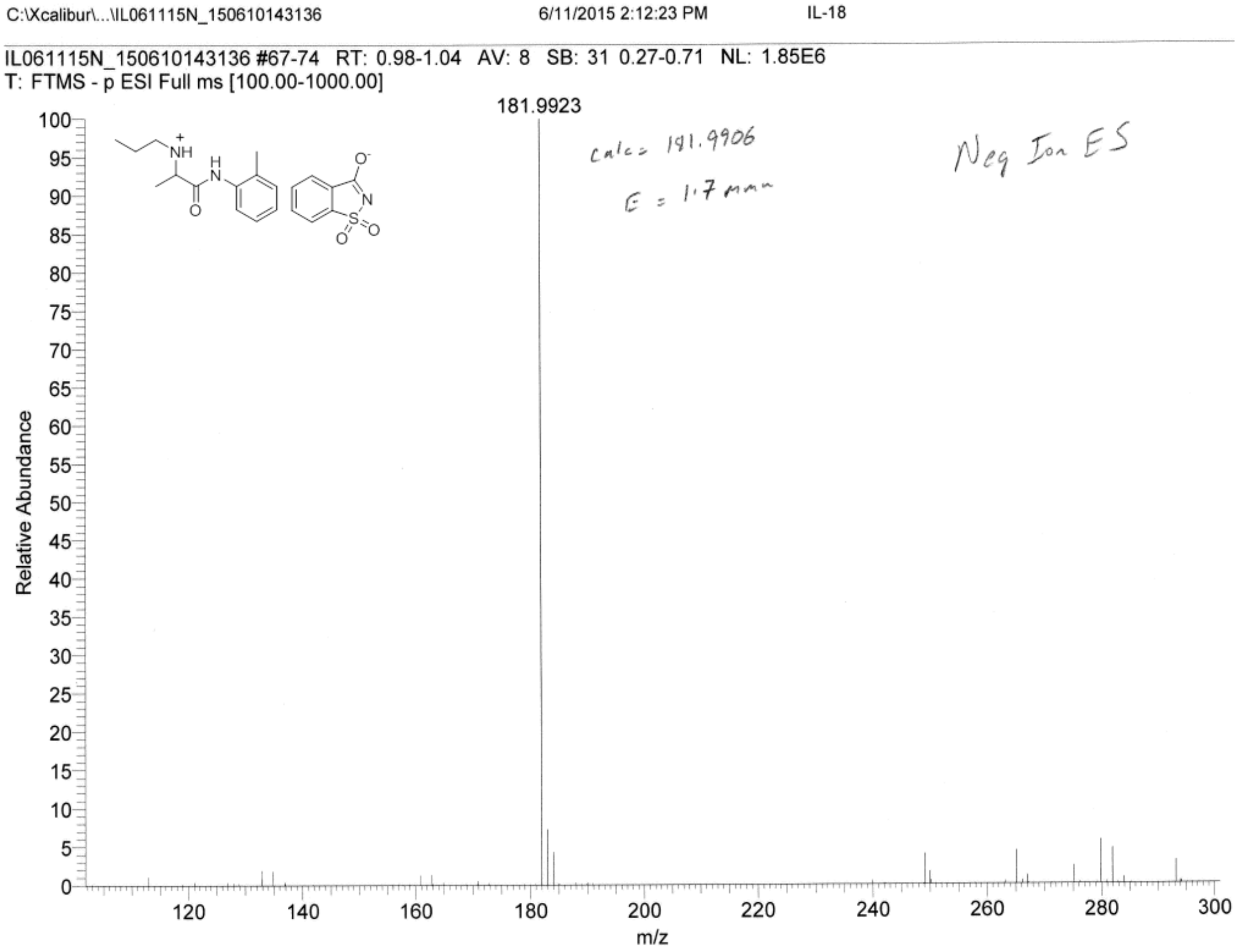

^1H spectrum for articaine saccharinate 4d

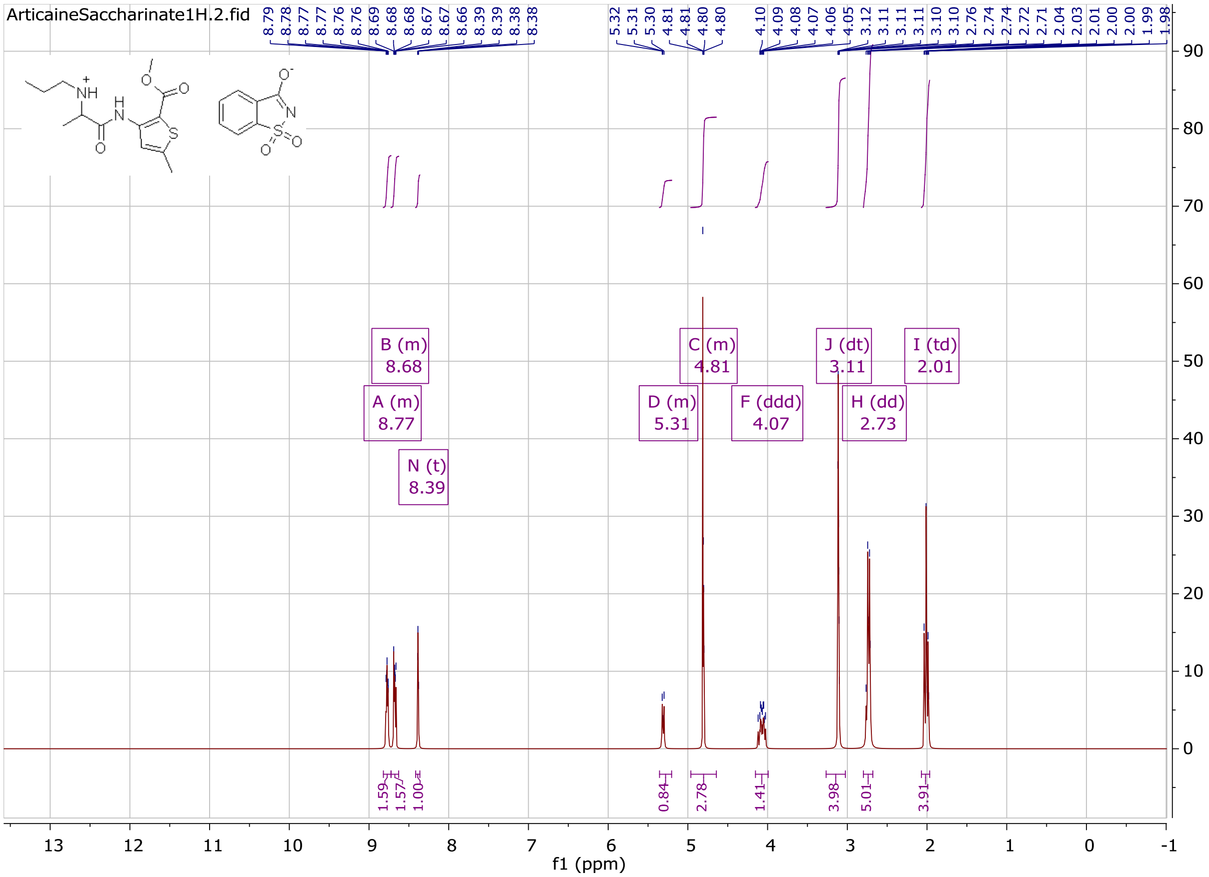

^{13}C spectrum for articaine saccharinate 4d

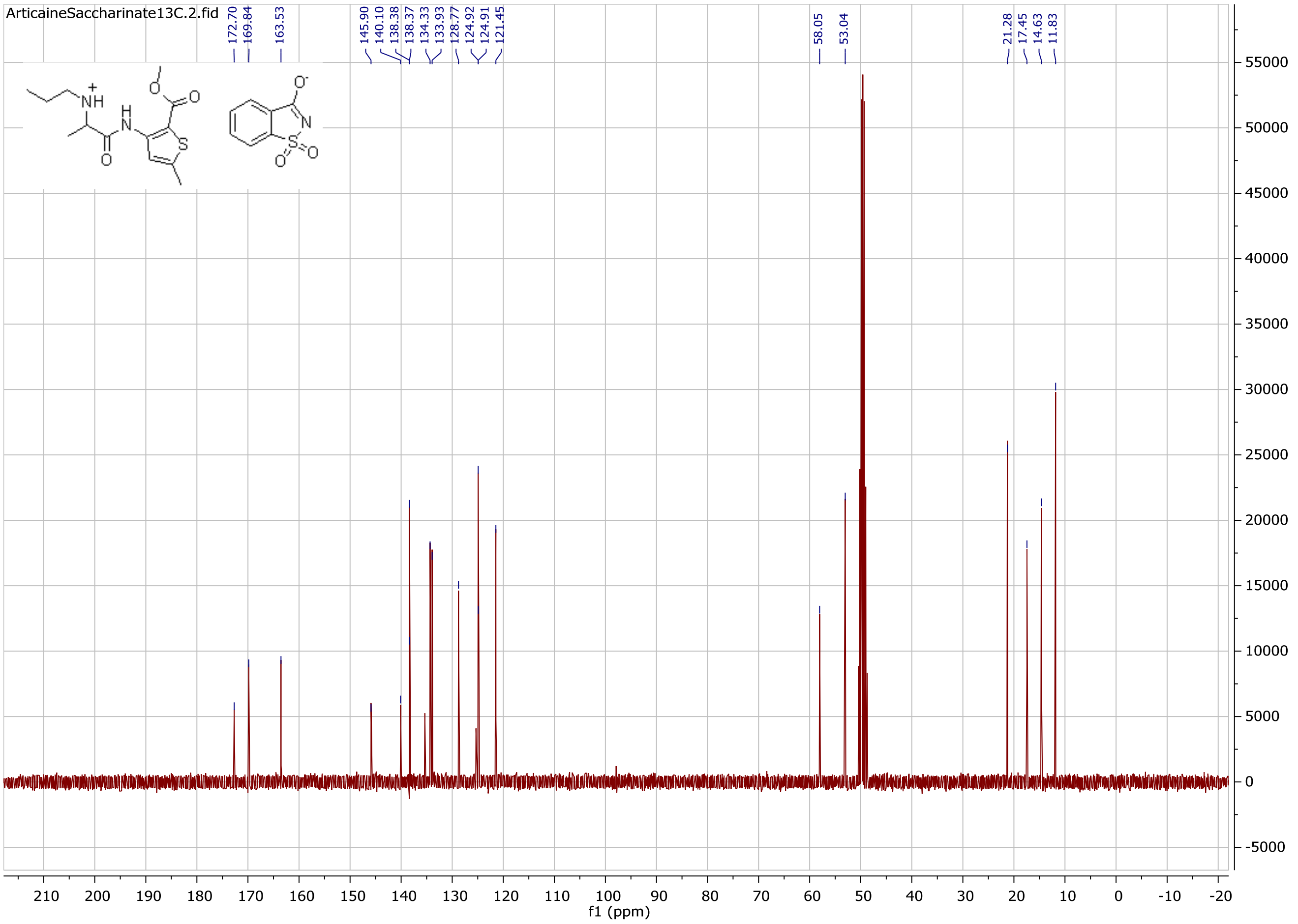

HRMS data for articaine saccharinate 4d

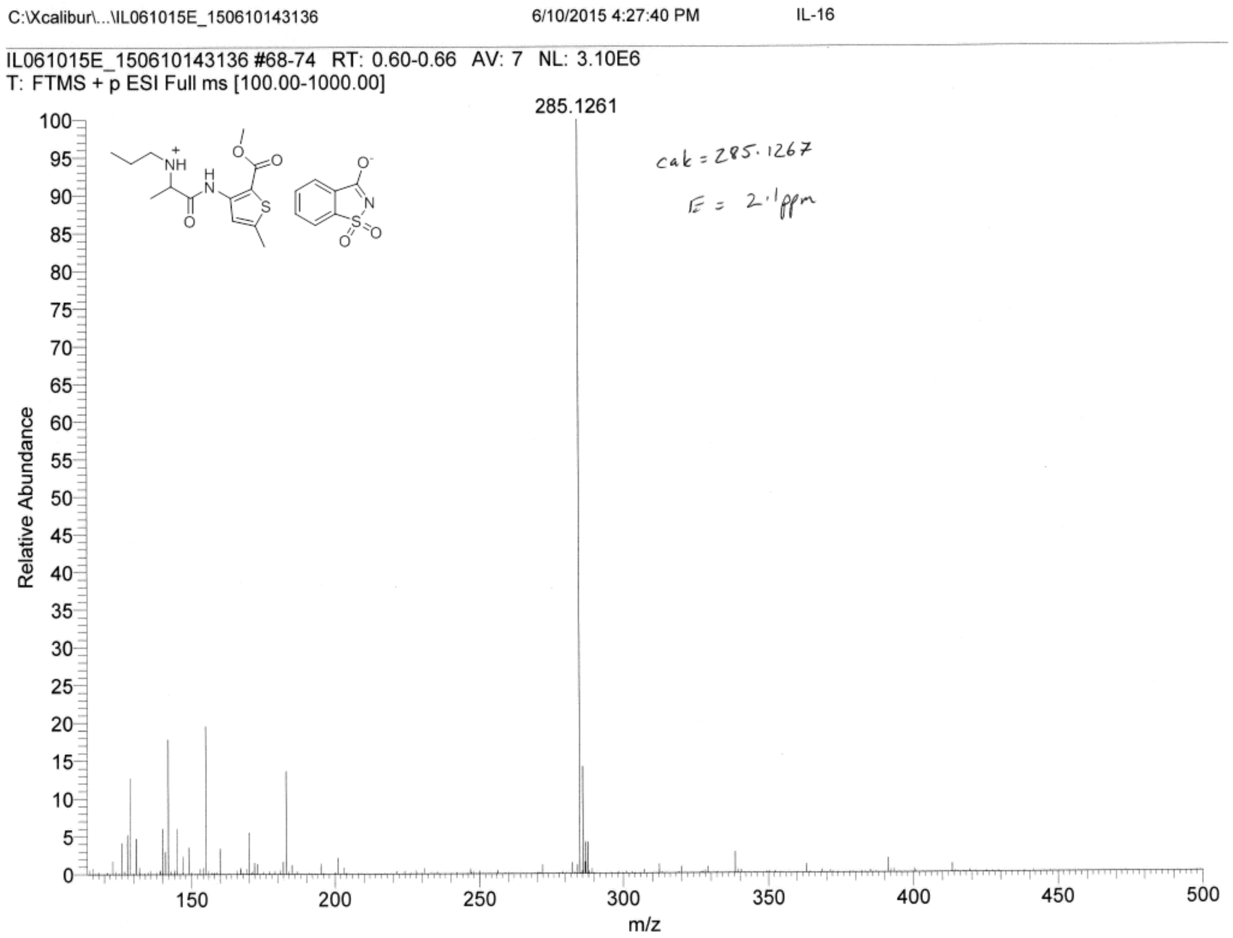

HRMS data for articaine saccharinate 4d

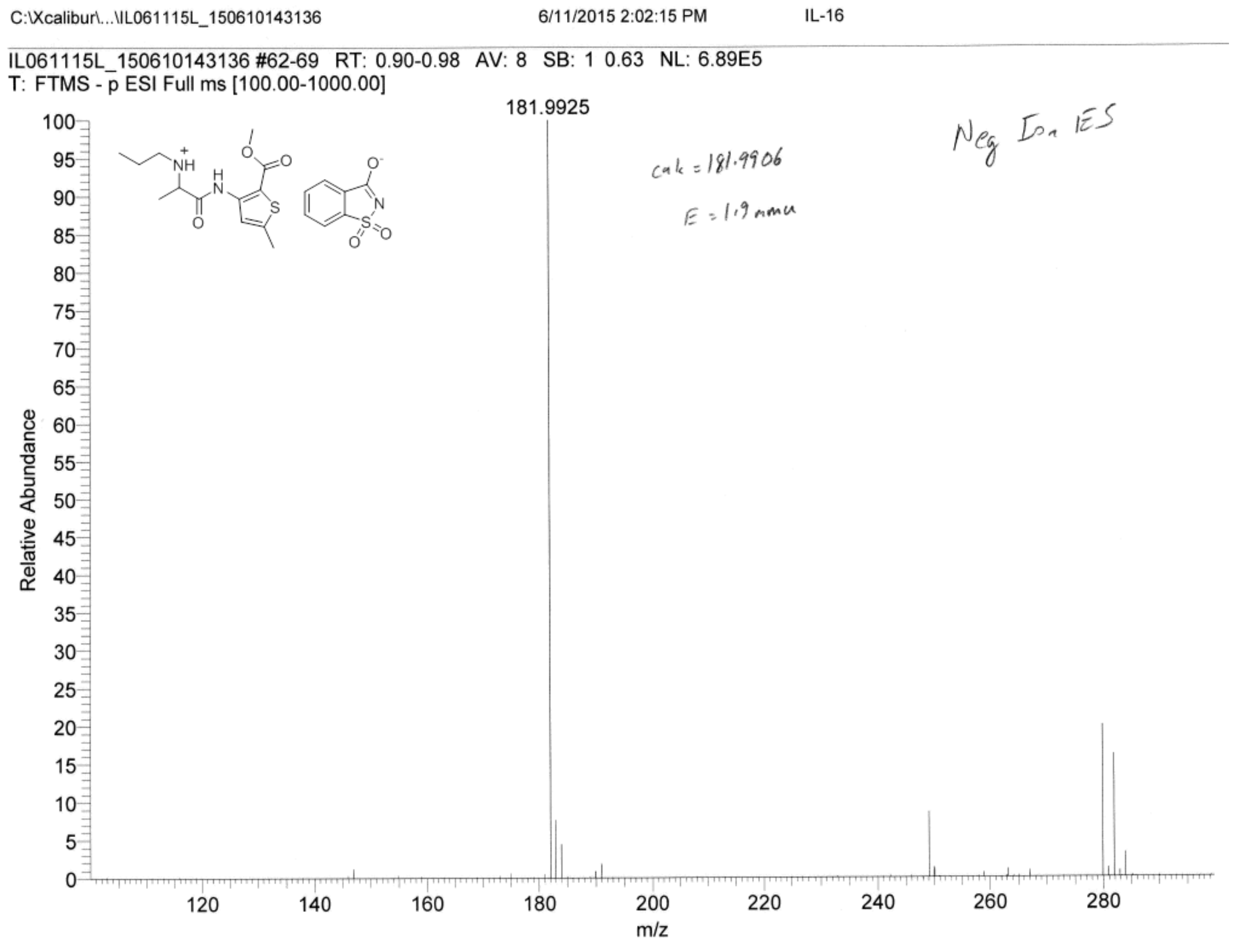

^{1}H spectrum for oxybuprocaine saccharinate 4e

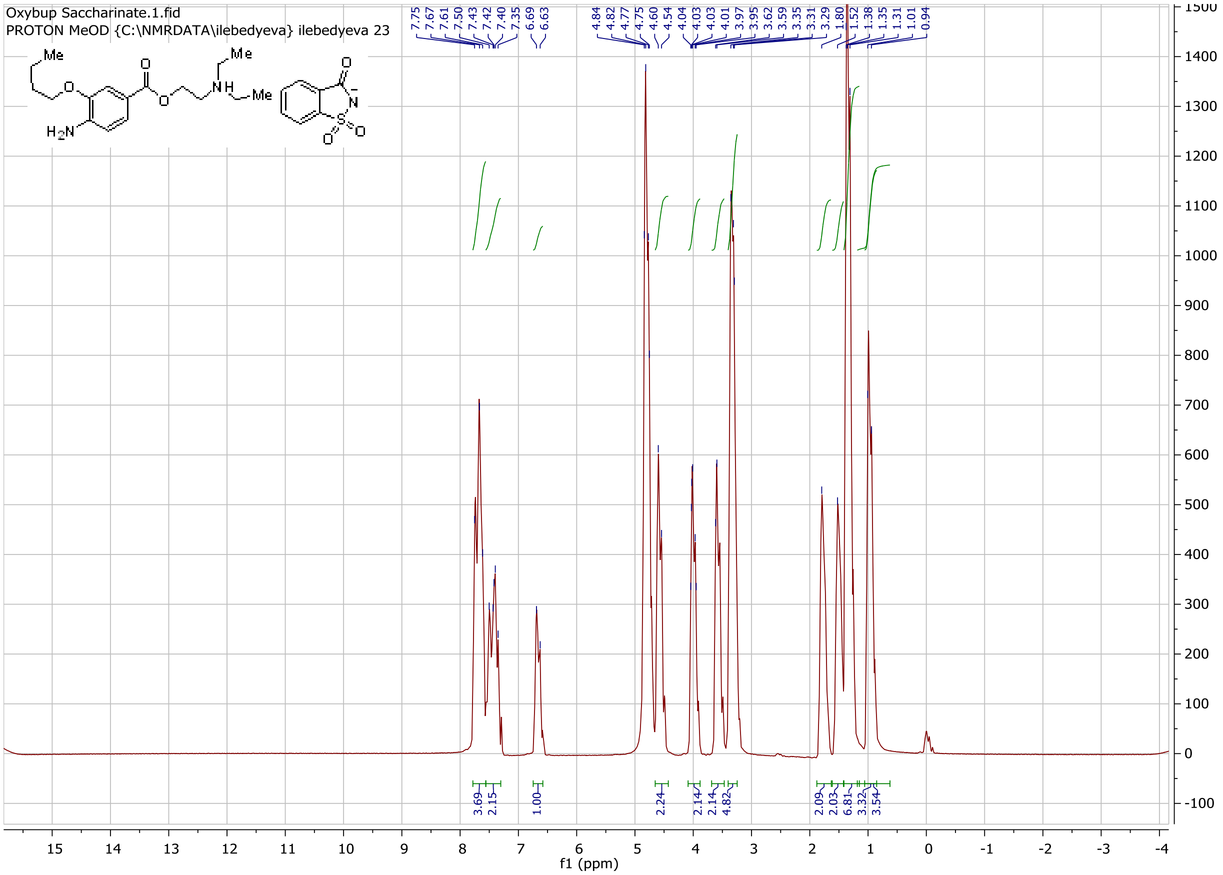

^{13}C spectrum for oxybuprocaine saccharinate 4e

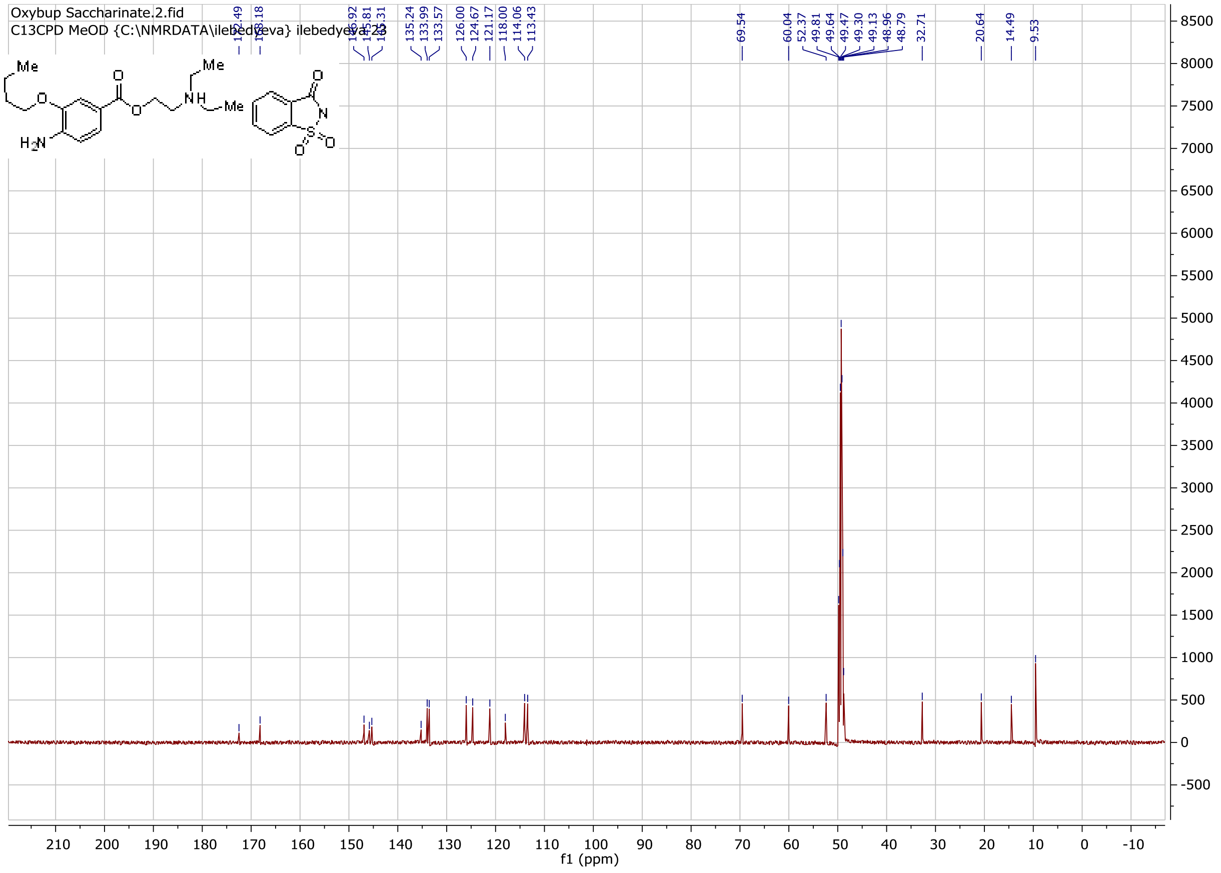

HRMS data for oxybuprocaine saccharinate 4e

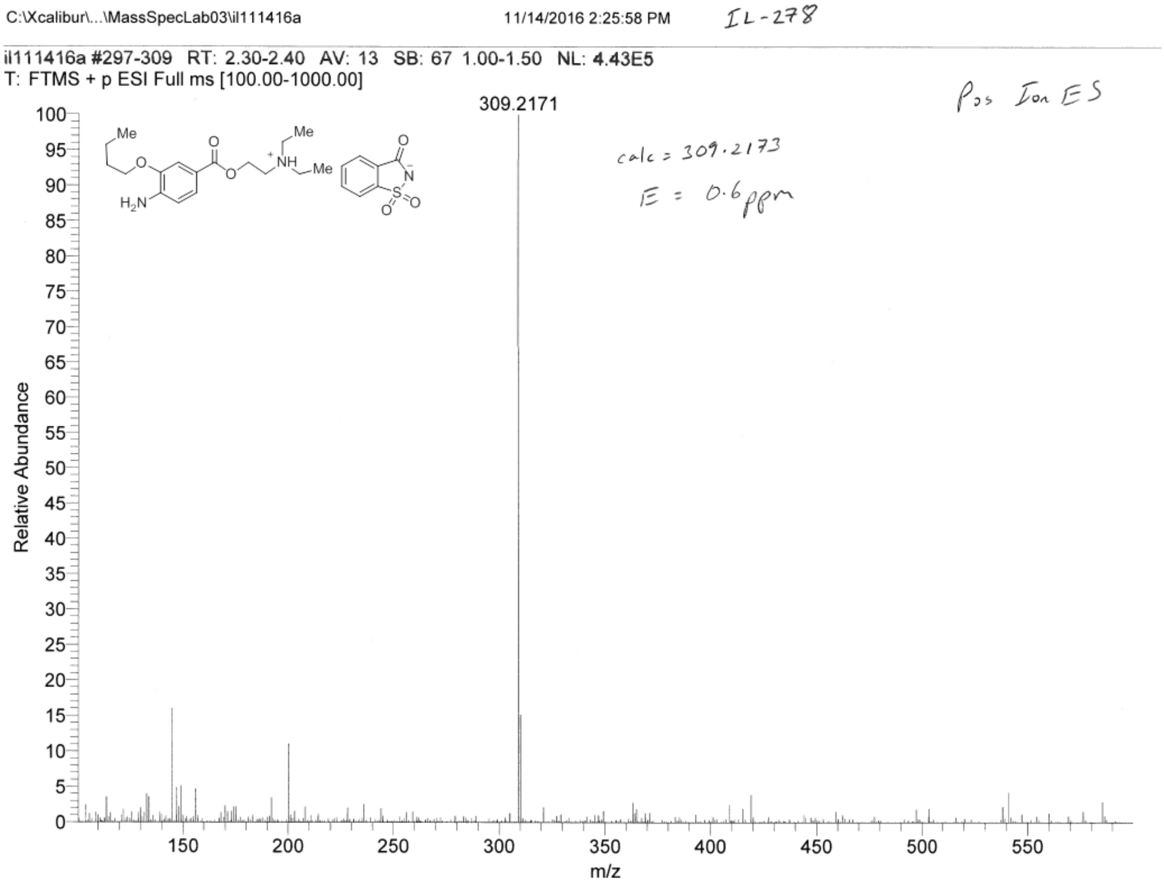

HRMS data for oxybuprocaine saccharinate 4e

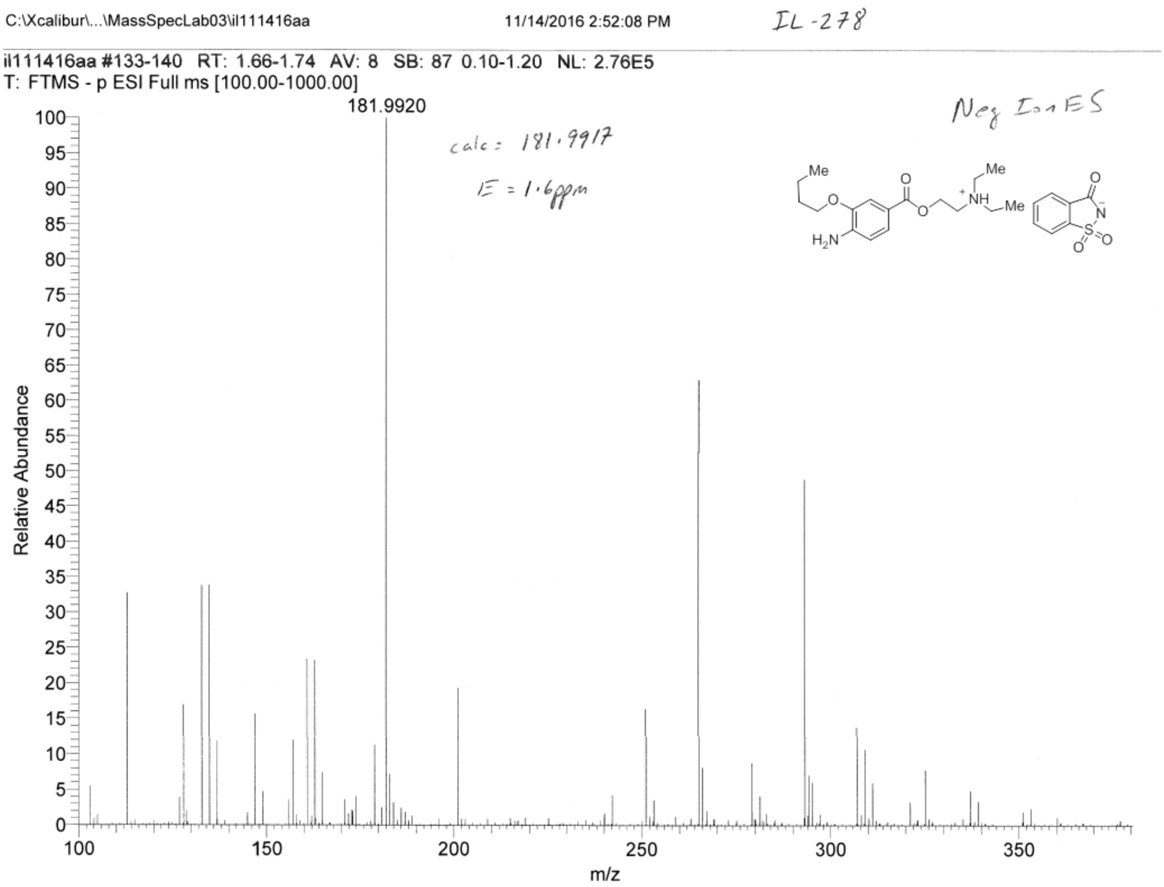

^{1}H spectrum for epinephrine acesulfamate 6a

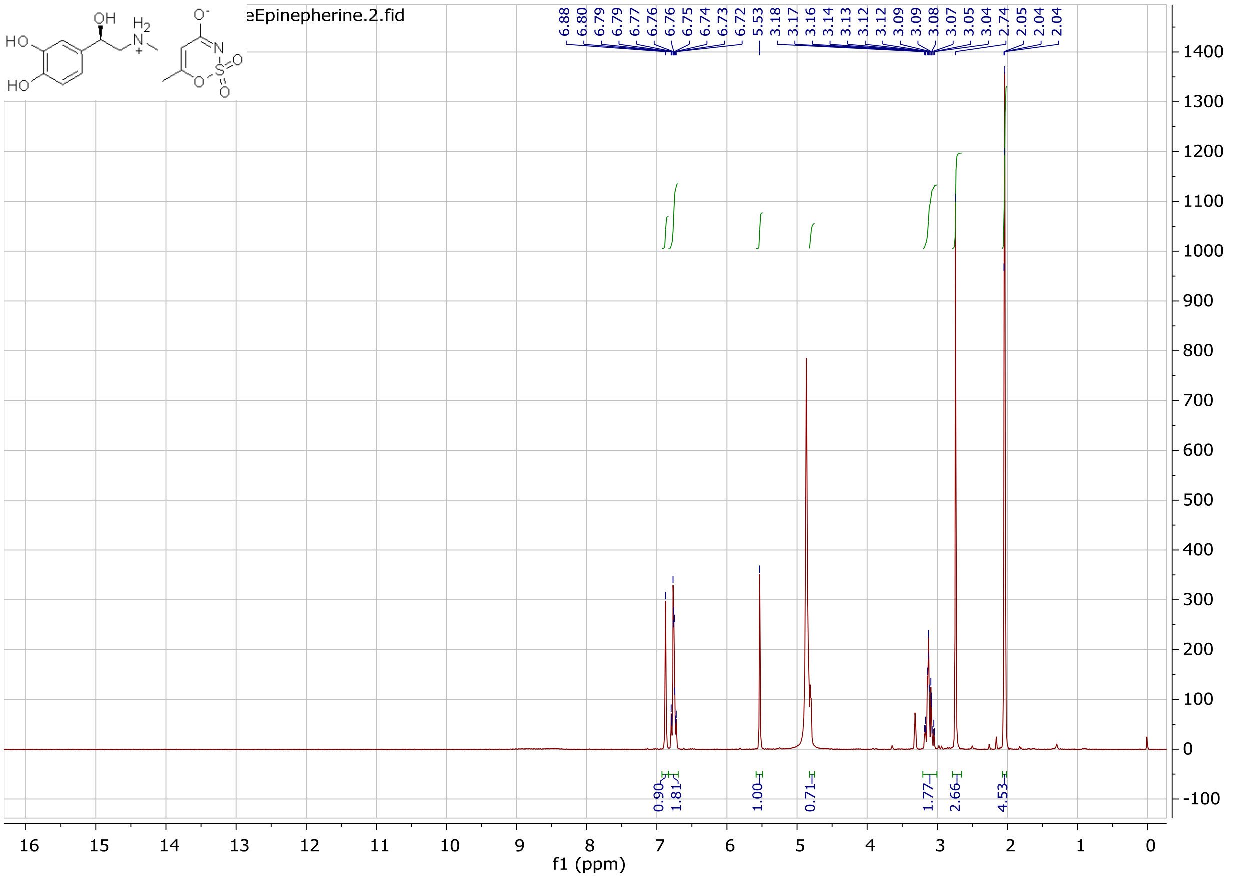

^{13}C spectrum for epinephrine acesulfamate 6a

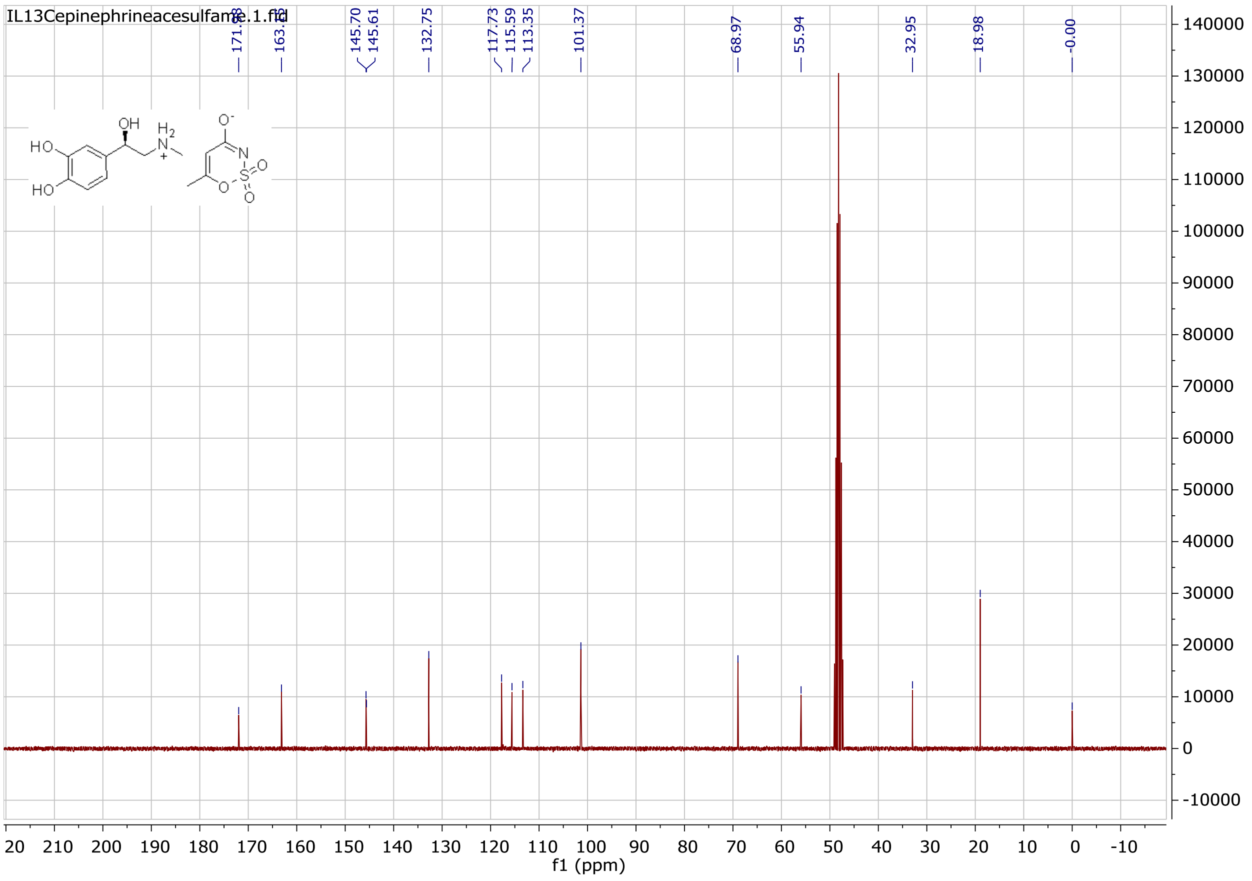

HRMS data for epinephrine acesulfamate 6a

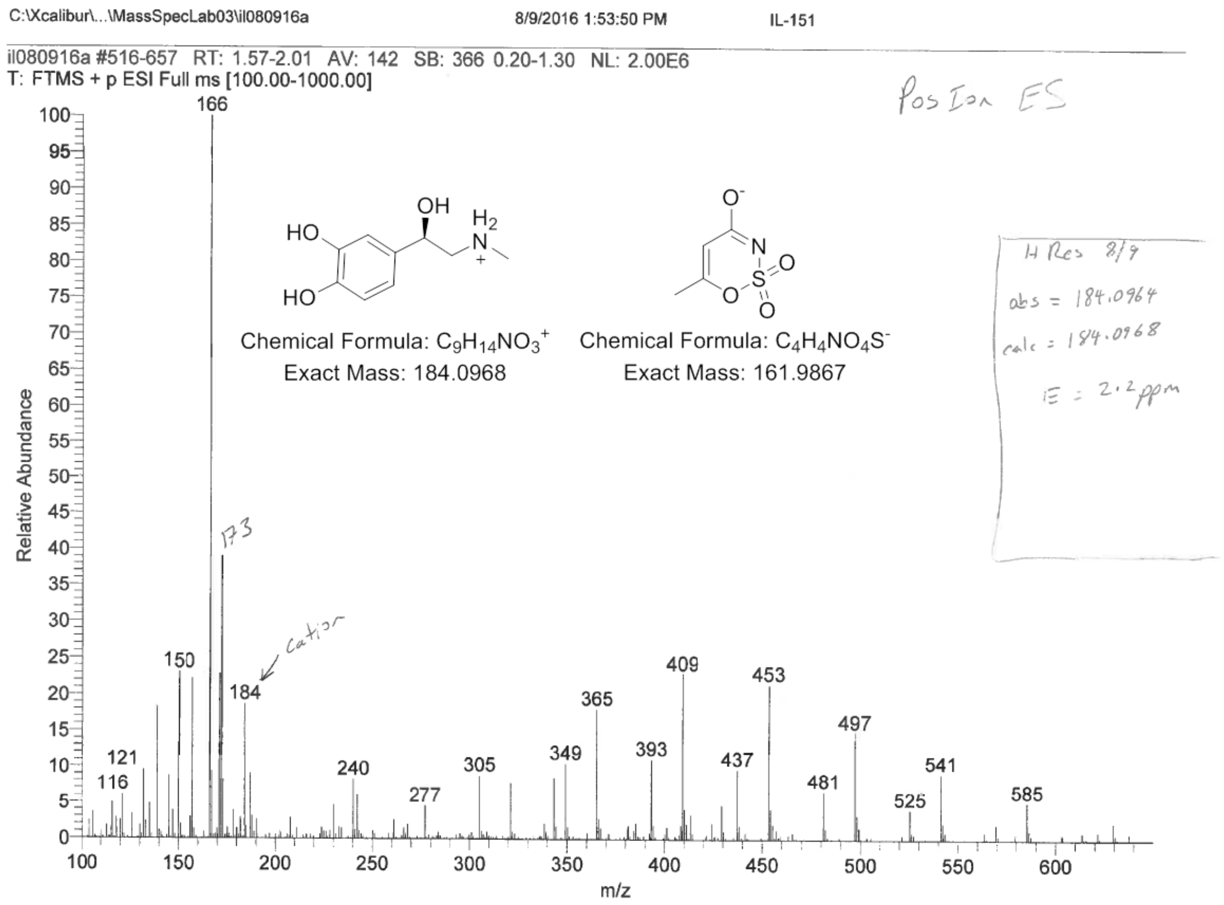

HRMS data for epinephrine acesulfamate 6a

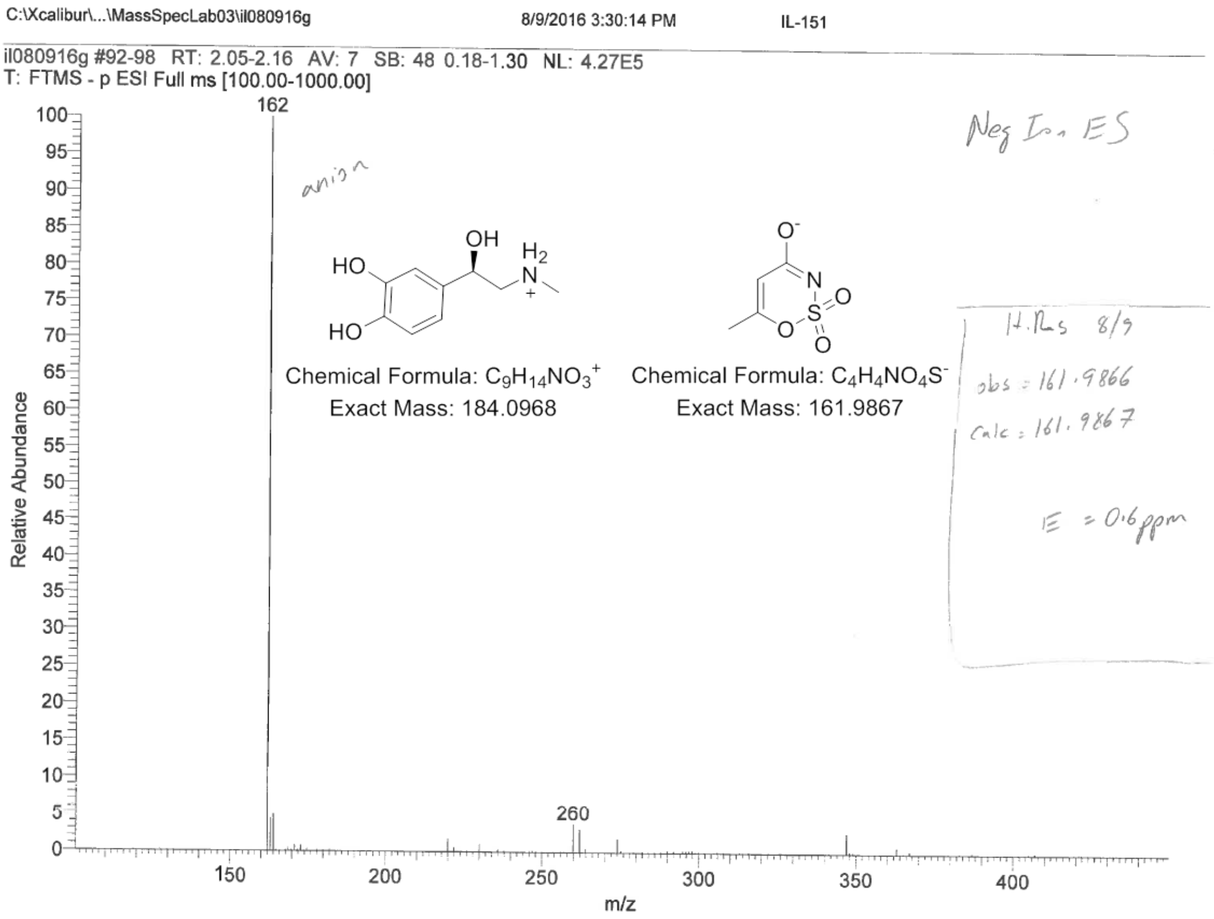

^{1}H spectrum for epinephrine saccharinate 6b

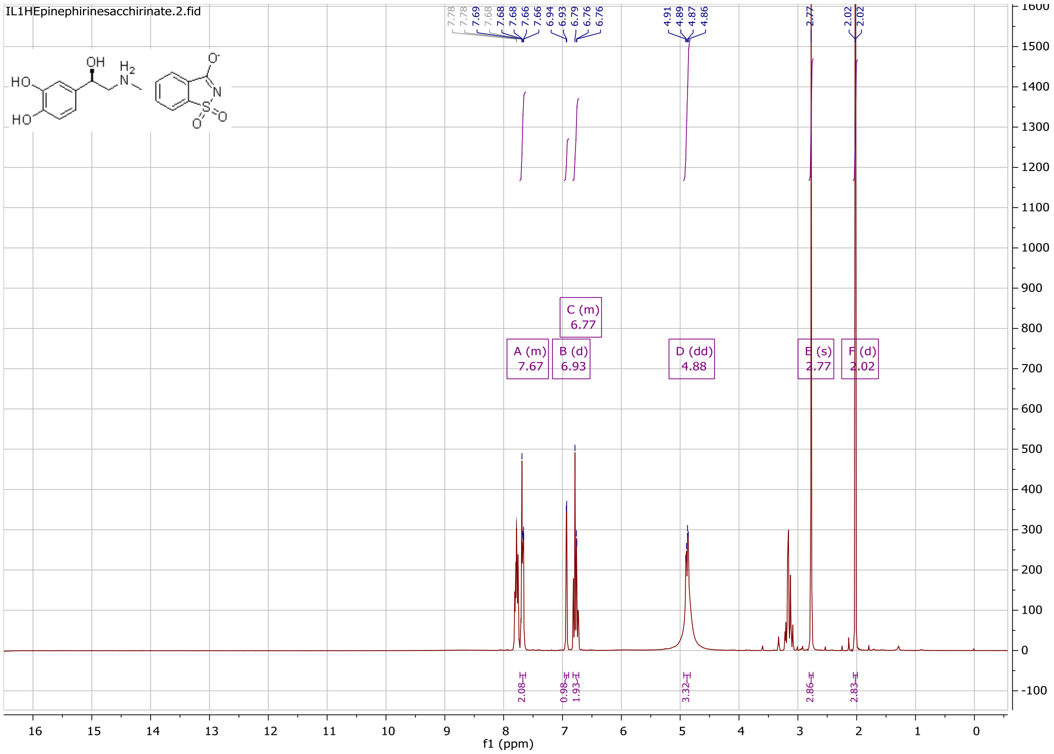

^{13}C spectrum for epinephrine saccharinate 6b

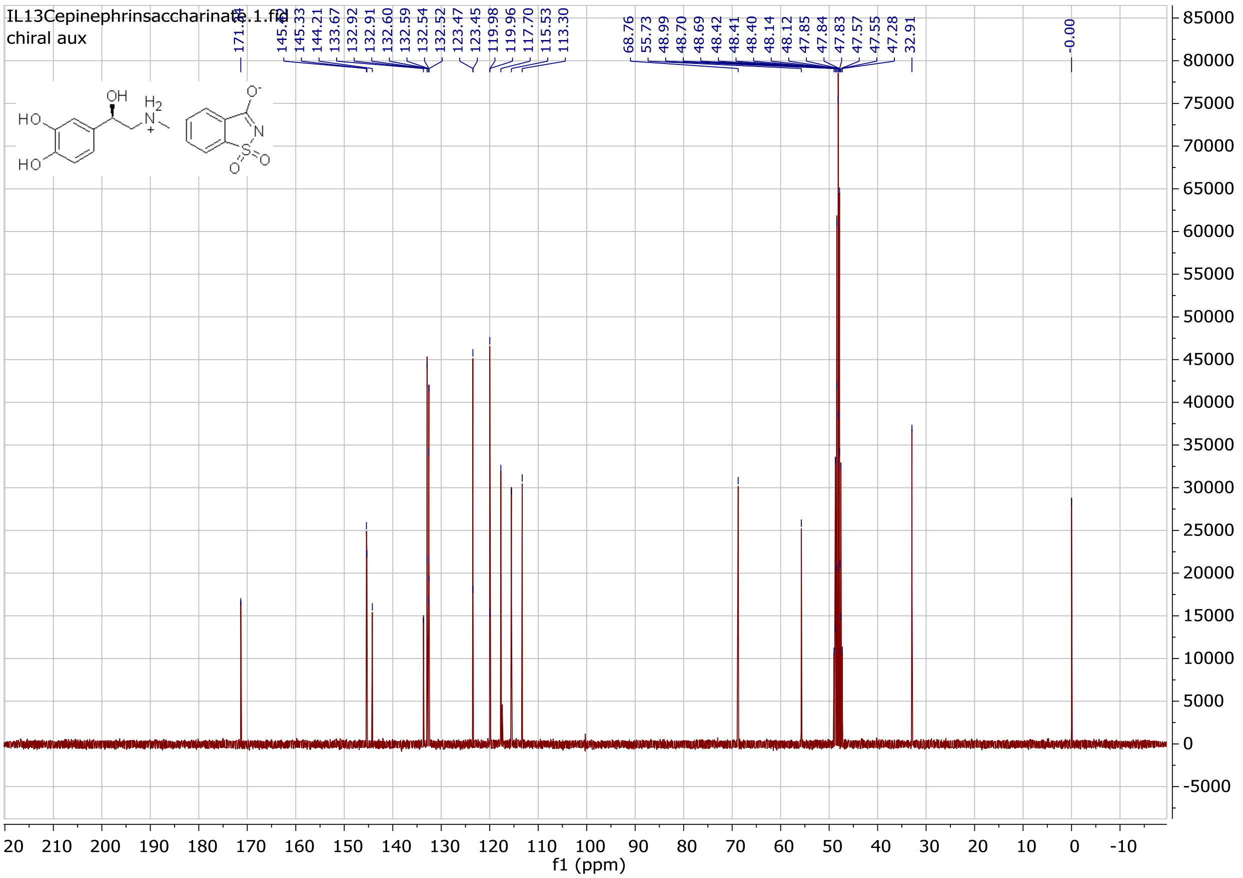

HRMS data for epinephrine saccharinate 6b

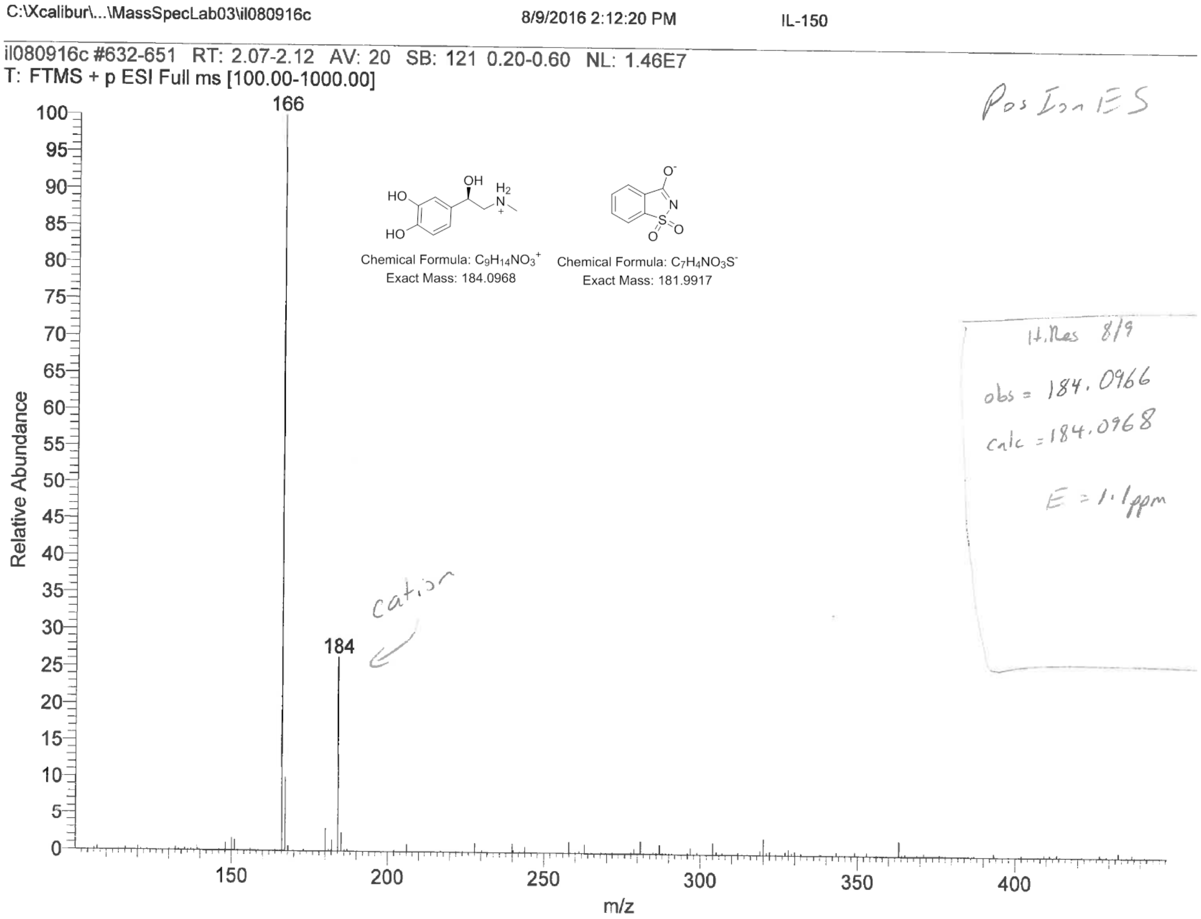

HRMS data for epinephrine saccharinate 6b

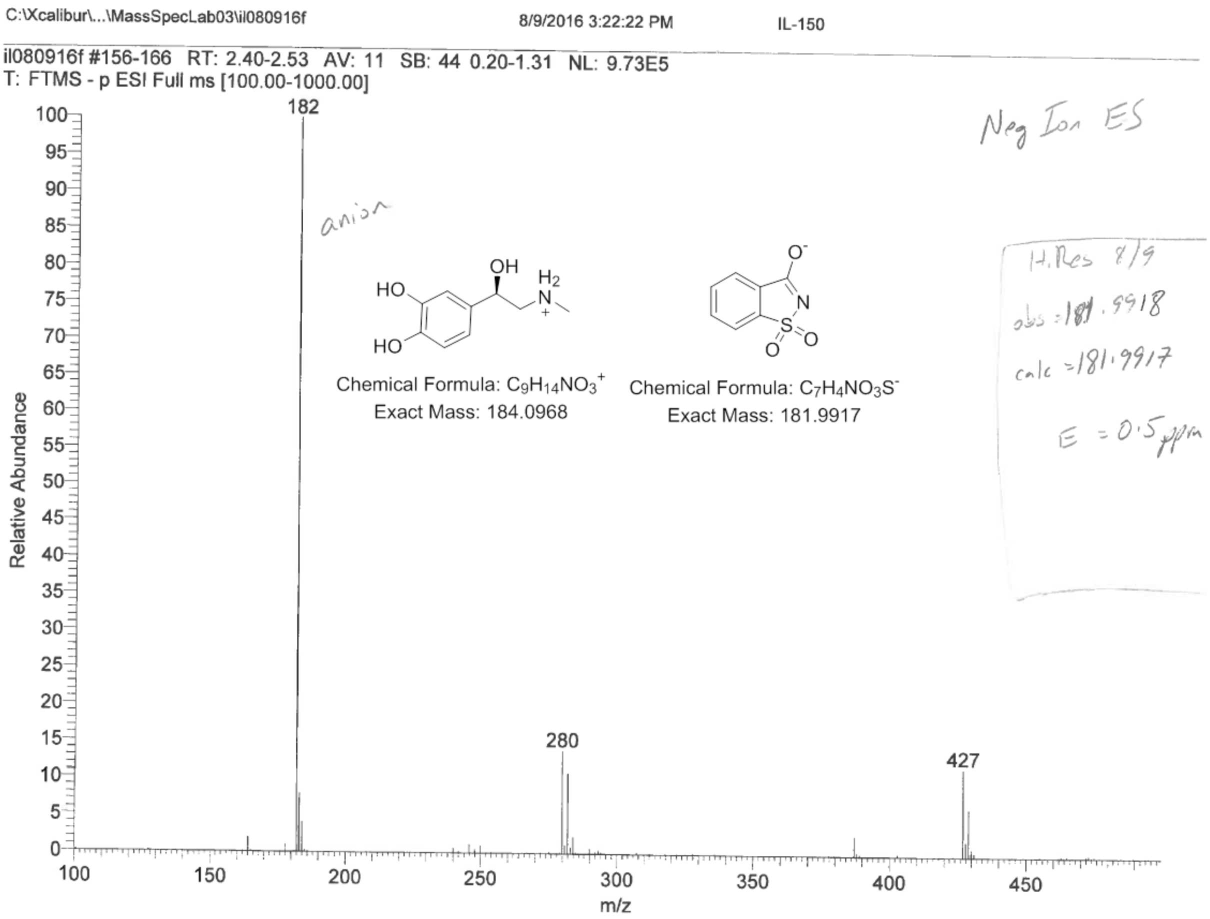

Supplement: Supplementary file 1 [file molecules-26-00983-s001.pdf]
